# Characterization of the adaptive immune response of donors receiving live anthrax vaccine

**DOI:** 10.1371/journal.pone.0260202

**Published:** 2021-12-20

**Authors:** Victoria V. Firstova, Anastasia S. Shakhova, Alena K. Riabko, Marina V. Silkina, Natalia A. Zeninskaya, Yana O. Romanenko, Maksim A. Marin, Methun M. Rogozin, Alena S. Kartseva, Ivan A. Dyatlov, Igor G. Shemyakin

**Affiliations:** Laboratory of Molecular Biology, Federal Budget Institution of Science «State Research Center for Applied Microbiology and Biotechnology» of Federal Service of Consumer Right Surveillance & Human Welfare, Ministry of Health & Welfare, Obolensk, Moscow Region, Russian Federation; Banaras Hindu University Institute of Medical Sciences, INDIA

## Abstract

Live anthrax vaccine containing spores from attenuated strains STI-1 of *Bacillus anthracis* is used in Russia and former CIS (Commonwealth of Independent States) to prevent anthrax. In this paper we studied the duration of circulation of antibodies specific to spore antigens, the protective antigen (PA), the lethal factor (LF) and their domains (D) in donors’ blood at different times after their immunization with live anthrax vaccine. The relationship between the toxin neutralization activity level and the level of antibodies to PA, LF and their domains was tested. The effect of age, gender and number of vaccinations on the level of adaptive post-vaccination immune response has been studied. It was shown that antibodies against PA-D1 circulate in the blood of donors for 1 year or more after immunization with live anthrax vaccine. Antibodies against all domains of LF and PA-D4 were detected in 11 months after vaccination. Antibodies against the spores were detected in 8 months after vaccination. A moderate positive correlation was found between the titers of antibodies to PA, LF, or their domains, and the TNA of the samples of blood serum from the donors.

## Introduction

Vaccination against *Bacillus anthracis* is considered one of the most effective preventive measures against anthrax. Only two types of anthrax vaccines are used in the world—live attenuated vaccines and subunit vaccines. Live anthrax vaccine contains live spores of the vaccine strain *Bacillus anthracis* STI that produces the anthrax toxin. Subunit cell-free vaccines BioThrax™ (i.e., Anthrax Vaccine Adsorbed (AVA)) and NuThrax™ manufactured by Emergent BioSolutions Inc., USA (that is at the 3rd stage of clinical trials) as well as Anthrax Vaccine Precipitated (AVP) manufactured by Porton Biopharma Ltd, UK, are all in the form of a sterile filtrate adsorbed on a carrier of the anthrax toxin obtained from a culture of vaccine anthrax strains. The above-mentioned vaccines do not contain live microorganisms.

In Russia, live anthrax vaccine (LAV) is used to prevent anthrax. Numerous experiments with animals have shown that the presence of toxin-neutralizing antibodies to the components of the lethal toxin (LT) of the anthrax pathogen—the protective antigen (PA) and the lethal factor (LF),—is an important factor in providing protection against anthrax infection. Passive administration of toxin-neutralizing antibodies to animals or to in vitro cultures (macrophage-like cell lines J774.1A and RAW264.7) has demonstrated a significant efficacy in neutralizing anthrax toxins by antibodies [1–5].

Both LT proteins—PA and LF—are characterized by a domain structure. PA (83 kDa) contains four domains, and each of them plays a unique role in the function of the toxin. Domain 1 (PA-D1, aa 1–258) contains the furin recognition site RKKR that is cleaved to release the N-terminal fragment with a mass of 20 kDa PA20 (PA-D1a, aa 1–167) that form the remaining PA63 (63 kDa). Upon release from PA20 it acquires the ability for oligomerization. After the removal of PA20, the remaining part of the PA-D1 domain (PA-D1b domain) forms a binding site to the effector subunits of LF and/or EF (edema factor). Domains PA-D2 (aa 259–487) and PA-D3 (aa 488–595) participate in oligomerization of PA63 with the formation of hepta- or octamers. PA-D3 also seems to play a role in an effector binding. PA-D2 is responsible for the formation of a pore through which the effector molecules move from the endosome into the cytosol. PA D3 and PA-D4 (aa 596–735) are involved in the binding of PA63 with a mass of 63 kDa with cell receptors [6].

LF is a protein with a molecular weight of 90 kDa containing 776 amino acid residues that span four different domains. N-terminal domain (LF-D1) facilitates the protein binding with PA prior to membrane translocation. This region has a homology of the sequence with N-terminal domain EF. That is not surprising given the fact that EF also binds to the same region of PA. The other EF and LF regions mediate the catalytic activity of these enzymes. In the case of LF domains 2, 3 and 4 (LF-D2, LF-D3 and LF-D4) form a 40 Å long groove that holds the N-terminal tail (16 amino acid residues) of mitogen-activated protein kinase kinases (MEKs), that are specific substrates for LF-D4. All MEKs, except MEK-5 (1–4, 6–7), are subject to cleavage; this cleavage and a subsequent deactivation of MAPK signaling pathways lead to a critical disruption of different cellular functions [7].

Thus, each of PA and LF domains performs a certain function. Blocking any of PA or LF domains can lead to inhibition of LT toxicity. TNA test is useful in predicting vaccine efficacy, since it measures the neutralizing activity of sera against the cytotoxic effect of LT. Various scientific studies show conflicting results in regard to the presence of a correlation between the level of antibodies to PA in the serum of vaccinated donors and capacity of this serum to neutralize the toxin [8–11].

Antibodies to anthrax spores also play a role in providing protective anti-anthrax immunity; as the antibodies opsonise the spores, this process contributes to the rapid elimination of the spores from the organism [12, 13].

LAV, AVA and AVP are effective but can cause side effects and require annual booster vaccinations to maintain a high level of specific immunity. In this regard, a range of new vaccines directed towards creation of a less reactogenic anthrax vaccine with the formation of a longer specific immunity continues. PA, LF, and spore antigen are promising components for the vaccines under development. To reduce reactogenicity and increase the stability of the vaccine, not full-length proteins are considered as its components, but individual domains. Understanding how to generate a long lasting protective immunity is a long-sought goal.

This study was designed to determine: 1) the domain specificity of the longest circulating antibodies to PA and LF in the blood of donors immunized with live anthrax vaccine; 2) relationships between the level of antibodies to PA or different PA domains and toxin neutralizing capacity of the serum of vaccinated donors; 3) the duration of vaccine acquired antibody responses to spore antigens of *B*. *anthracis*; 4) the impact of repeated vaccination over 10–20 previous years on the current level of specific immune response.

## Results

### Analysis of a donor group

We examined the samples of blood serum from 67 donors who had previously been vaccinated with LAV and the samples of blood serum from 21 healthy donors who had never been vaccinated with LAV. The number of vaccinations received, the average time since the last vaccination and the gender are presented in Table 1.

**Table 1:** Groups of donors with reference to gender, number of vaccinations and the time since the last LAV immunization.

Time since last vaccination	Gender		Number of vaccinations		Total number of donors in groups
	male	female	1–2	over 10	
**1–3 months**	9	7	6	10	16
**4–8 months**	9	10	7	12	19
**9–11 months**	8	7	7	8	15
**More than 1 year**	8	9	8	9	17
**Non-vaccinated**	10	11	-	-	21

Vaccinated donors were divided into 4 groups depending on the time since the last vaccination. In all the groups there was approximately the same distribution in the number of men and women, and the number of vaccinations they received.

### Levels of specific antibodies to PA, LF and to surface spore antigens in the samples of blood serum from vaccinated donors

**Fig 1:**
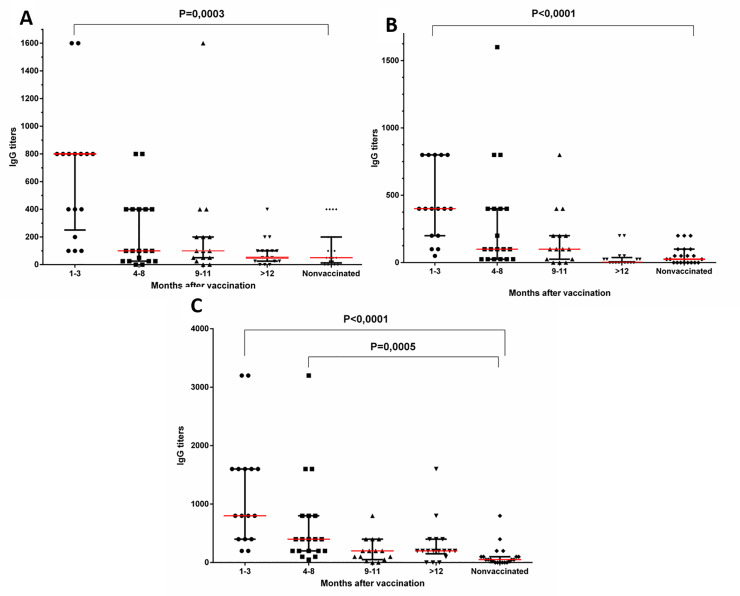
Levels of specific IgG to full-length PA antigens of *B*. *anthracis* (A), LF of *B anthracis* (B), spores of *B*. *anthracis* (C) in the samples of blood serum from the donors. The data are presented by a median titer with an interquartile range as a characteristic of the distribution of values in the groups. The distribution was analysed using the Shapiro-Wilk test. The data were analysed using the Kruskal-Wallis test with multiple Dunn’s comparisons in a One-Way ANOVA. See data in S1–S3 Datasets.

The detected levels of PA-specific IgGs in the samples of blood serum from each group of the donors varied widely (Fig 1). In the control group, the blood serum from some donors showed LAV vaccine-unrelated preexisting antibodies to PA, LF and spore antigens of *B*. *anthracis*. Therefore, the median titers of the control group and of the groups of vaccinated donors were compared (Fig 1). A statistically significant presence of the antibody response to PA and LF (Fig 1A and 1B) was detected only in the group of donors whose blood serum was collected in 1–3 months after vaccination (P <0.0001). The number of samples of the blood serum seropositive to PA and LF decreased in 4–8 months or more after immunization with LAV.

LAV contains live spores of *B*. *anthracis* STI strain. The administration of these spores as part of the vaccine stimulates the synthesis of antibodies also to surface antigens of *B*. *anthracis* spores (Fig 1C). Our studies showed that antibodies to antigens of *B*. *anthracis* spores were also detected in the blood of the donors in 4–8 months after vaccination.

### Antibody levels to PA and LF domains of *B*. *anthracis* in the samples of blood serum from the donors at different times after their vaccination

**Fig 2:**
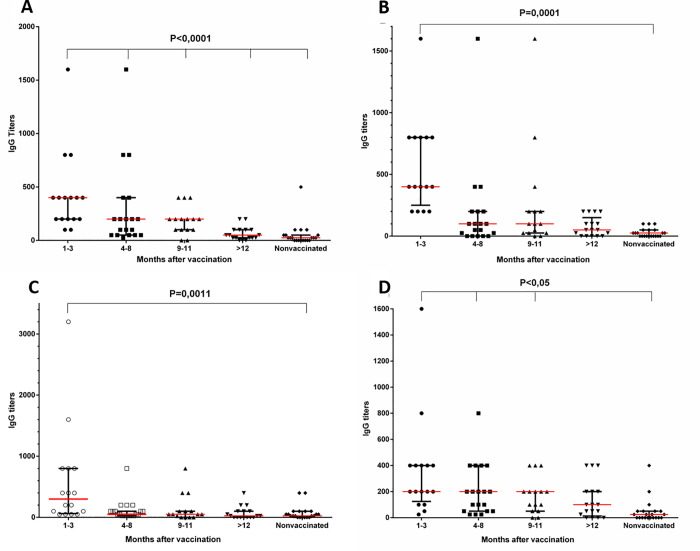
Levels of specific IgG to I (A), II (B), III (C), IV (D) PA domains of *B*. *anthracis* in the samples of blood serum from the donors. The data are presented by a median titer with an interquartile range as a characteristic of the distribution of values in the groups. The distribution was analysed using the Shapiro-Wilk test. The data were analysed using the Kruskal-Wallis test with multiple Dunn’s comparisons in a One-Way ANOVA. See data in S4–S7 Datasets.

As the results of the studies showed, antibodies to PA-D1 were detected in the samples of blood serum from vaccinated donors at all periods of the studies (P <0.0001, Fig 2A). Antibodies to PA-D4 circulated in the blood of the majority of the donors during 9–11 months after vaccination (P <0.0001, Fig 2D). Antibodies to PA-D2 and PA-D3 domains were detected no later than in 1–3 months after immunization with LAV (P <0.0001, Fig 2C).

It should be noted that despite the absence of statistically significant differences between the samples of blood from vaccinated donors and the samples of the control group donors in the level of IgG titers to PA-D2 and PA-D3 during 9–11 months after vaccination, some individuals from the groups of vaccinated donors had sufficiently high titers of IgG to PA-D2 and PA-D3 (1:800–1:1600).

**Fig 3:**
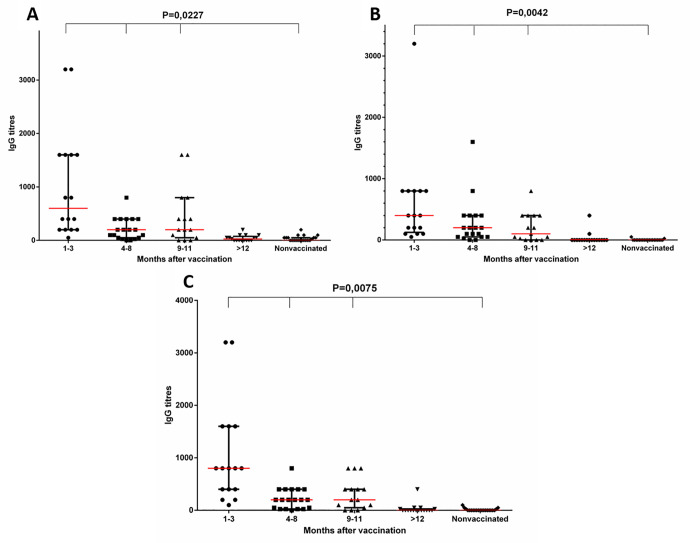
Levels of specific IgG to I (A), II—III (B), IV (C) LF domains of *B*. *anthracis* in the samples of blood serum from the donors. The data are presented by a median titer with an interquartile range as a characteristic of the distribution of values in the groups. The distribution was analysed using the Shapiro-Wilk test. The data were analysed using the Kruskal-Wallis test with multiple Dunn’s comparisons in a One-Way ANOVA. See data in S8–S10 Datasets.

Specific antibodies to all LF domains were detected in the studied samples of blood serum from most donors during 9–11 months after vaccination (Fig 3).

### Toxin-neutralizing activity of the samples of blood serum obtained from the donors at different times after their immunization against anthrax

**Fig 4:**
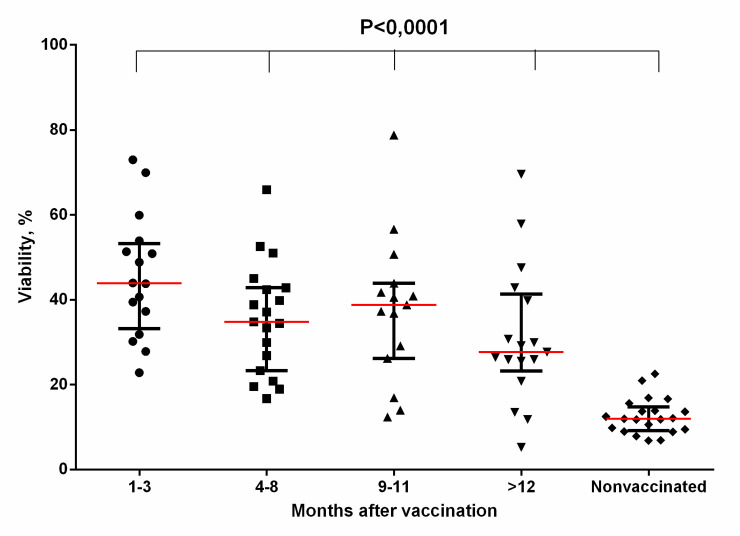
Toxin-neutralizing activity of the samples of blood serum (dilution 1:25) from the donors vaccinated against anthrax. The data were analysed using the Kruskal-Wallis test with multiple Dunn comparisons in a One-Way ANOVA. See data in S11 Dataset.

Fig 4 presents the data on the toxin-neutralizing activity (TNA) of the samples of blood serum from the donors at different times after their immunization with LAV. Fig 4 shows the results for 1:25 serum dilutions. Serum dilutions of 1:50 and 1:100 did not show TNA.

The samples of blood serum from most donors were characterized by the ability to neutralize LT of *B*. *anthracis* at all periods of the study. The median of TNA level in the samples of blood serum in 1–3 months after immunization with LAV corresponded to 45.35%, in 4–8 months–to 35.49%, in 9–11 months–to 36.83%, in 12–18 months–to 30.74%.

A comparative analysis of TNA of the samples of blood serum from the donors at different times after vaccination showed the absence of significant differences between the groups of immune serum (Table 2).

**Table 2:** Multiple comparison of the average ranks of all groups of the donors.

Dunn’s multiple comparisons test	Mean rank diff,*	Significant	Adjusted P Value
1–3 vs. 4–8	13,29	No	> 0,9999
1–3 vs. 9–11	11,61	No	> 0,9999
1–3 vs. over 1 year	21,52	No	0,1563
**1–3 vs. the control**	**51,57**	**Yes**	**< 0,0001**
4–8 vs. 9–11	-1,674	No	> 0,9999
4–8 vs. over 1 year	8,232	No	> 0,9999
**4–8 vs. the control**	**38,29**	**Yes**	**< 0,0001**
9–11 vs. over 1 year	9,906	No	> 0,9999
**9–11 vs. the control**	**39,96**	**Yes**	**< 0,0001**
**over 1 year vs. the control**	**30,06**	**Yes**	**0,0031**

* The data were calculated using GraphPad Prism 6.0 software.

### Correlation analysis between the titers of specific antibodies in the samples of blood serum and its toxin-neutralizing activity

**Fig 5:**
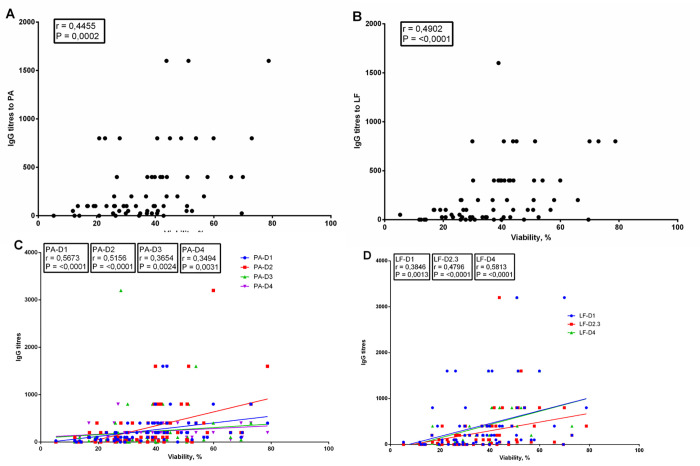
Correlation analysis between the toxin-neutralizing activity of the samples of blood serum from the donors and antibody titers against PA (A), LF (B), PA domains (C) and LF domains (D). See data in S12–S15 Datasets.

As it can be seen from the data presented in Fig 5 and Table 3, a moderate positive correlation was found between the titers of antibodies to PA, LF or their domains, and the TNA of the samples of blood serum from the donors.

**Table 3:** A summary table of the values of parameters r and P according to the result of the Spearman test.

Correlation	r	P value (two-tailed)
IgG titres to PA full-length vs. TNA	0,4455*	0,0002**
IgG titres to LF full-length vs. TNA	0,4902*	<0,0001***
IgG titres to PA-D1 vs. TNA	0.5673*	<0,0001***
IgG titres to PA-D2 vs. TNA	0,5156*	<0,0001***
IgG titres to PA-D3 vs. TNA	0,3654*	0,0024**
IgG titres to PA-D4 vs. TNA	0,3494*	0,0038**
IgG titres to LF-D1 vs. TNA	0,3846*	0,0013**
IgG titres to LF-D2.3 vs. TNA	0,4796*	<0,0001***
IgG titres to LF-D4 vs. TNA	0,5813*	<0,0001***

* moderate positive correlation (r = 0,3≤0,69).

** medium strength statistical significance.

*** strong statistical significance.

#### The effect of the number of vaccinations on TNA of the blood serum of the donors

Though the groups were represented by not a big number of samples of donors’ blood, we have compiled a table (Table 4) that shows the level of TNA of the samples of blood serum from the donors who were immunized against *B*. *anthracis* 1–2 or many times at different times after vaccination.

**Table 4:** The effect of the number of vaccinations on the TNA of the samples of blood serum from the donors.

TNA of the serum, %	Time after the last vaccination, months							
	1–3		4–8		9–11		> 12	
	Number of vaccinations, numbers							
	1–2	> 10	1–2	> 10	1–2	> 10	1–2	> 10
**51–100**	1	**5**	1	2	0	**3**	0	**2**
**26–50**	4	5	5	6	4	5	3	**5**
**12–25**	1	0	1	4	3	0	3	2
**Less than 12**	0	0	0	0	0	0	2	0
**Total**	6	10	7	12	7	8	8	9

A comparative analysis of the samples of blood serum showed that in 1–3 months after the last vaccination 5 out of 10 samples of blood serum from the donors who were vaccinated many times neutralized the lethal toxin of *B*. *anthracis* by more than 51%. In the group of the donors who were vaccinated one or two times only one of the 6 analyzed samples of serum showed TNA of more than 51%. In the samples of blood serum taken at later periods after vaccination TNA decreased regardless of the numbers of administration of the vaccine. However, the effectiveness of TNA of serum was more expressive in the group of the donors who were vaccinated many times. In the samples of blood serum from 15 donors vaccinated no more than 2 times TNA was not detected in any of the analyzed samples of serum in the amount of more than 51% in 9–11 months after the administration of LAV. Five samples of blood serum of the 17 ones taken from the donors who were vaccinated many times, showed TNA of more than 51% in an in vitro test in 9–11 months after the last administration of LAV.

#### The effect of age and gender on the development and duration of anti-anthrax post-vaccination immunity

**Fig 6:**
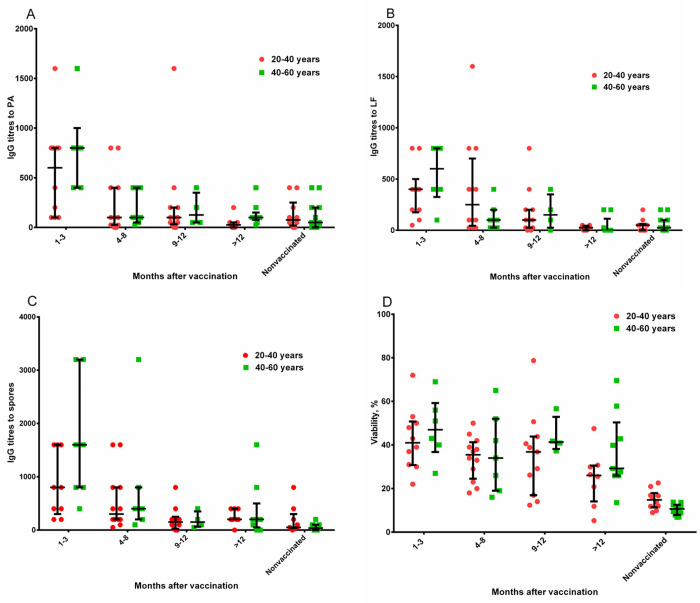
Analysis of the effect of age on the development and duration of anti-anthrax post-vaccination immunity. Statistical analysis was performed using a Two-way ANOVA with Tukey’s multiple comparison (determination of significance and confidence intervals). The histograms show the mean and the confidence interval (CI) as an interval estimate of the general frame. See data in S16–S19 Datasets.

**Fig 7:**
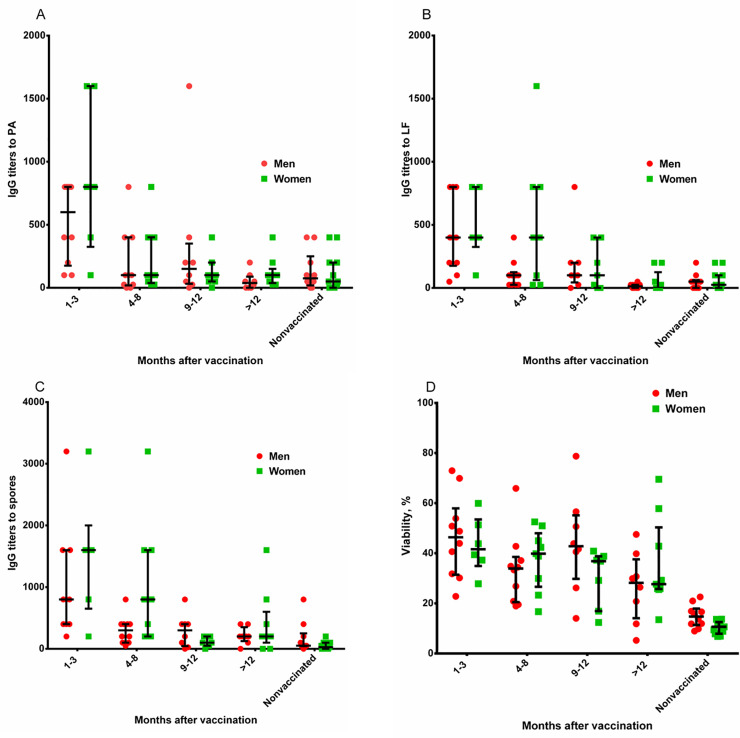
The effect of gender on the development of anti-anthrax post-vaccination immunity and the duration of circulation of IgG to PA (A), LF (B) and *B*. *anthracis* spores (C), as well as the level of TNA of the samples of blood serum from the donors (D). Statistical analysis was performed using a Two-way ANOVA with Tukey’s multiple comparison (determination of significance and confidence intervals). The histograms show the mean and the confidence interval as an interval estimate of the general frame. See data in S20–S23 Datasets.

The donors were divided into 2 groups according to their age: 20-40-year-olds and 40-60-year-olds; and according to gender: men and women. As a result of statistical analysis, no significant differences between the groups at any time after vaccination were detected (Figs 6 and 7).

## Discussion

Antibodies against PA, LF and spore antigens were detected in the samples of blood serum from the donors in early periods (1–3 months) after immunization with LAV. The levels of antibodies in the serum from the donors decreased with an increase in the time period after vaccination. Antibodies against spore antigens were detected in the serum of the majority of the donors during the period of up to 8 months after vaccination. Analysis of the domain specificity of antibodies against PA and LF showed that in 1–3 months after vaccination antibodies against all PA and LF domains circulated in the blood serum of the majority of the donors. Antibodies against all LF domains circulated in the blood of the donors during 11 months after vaccination. The longest circulating antibodies against PA were characterized by domain specificity of PA-D4 (9–11 months after vaccination) and PA-D1 (detected in 12–18 months after vaccination).

Long-term circulating antibodies against PA were characterized by the domain specificity of PA-D4 (9–11 months after vaccination) and PA-D1 (detected 12–18 months after vaccination). Antibodies against PA-D1 and PA-D4 play an important role in the anthrax toxin neutralization. The importance of the contribution to the neutralization of LT by antibodies directed against PA-D1b was shown in the experiments of Abbound N (2009) [14].

Most of PA-D1 is PA20 (PA-D1a) that is cleaved from full-length PA83 by furin-like proteases. It is believed that the PA20 region does not play a functionally important role in the pathogenesis of anthrax toxin. Nevertheless, the binding of antibodies to individual PA20 epitopes leads to the neutralization of LT by the inhibition of the proteolysis of PA83 [15]. The residual part of the domain I (PA-D1b) plays an important role in the binding of PA and LF [16].

The longest circulating antibodies against PA-D4 in the blood of those vaccinated can also play a significant role in the neutralization of the anthrax toxin. Our studies have shown that IgG specific to PA-D4 are detected in the blood serum of vaccinated people during the period of up to 11 months after the last immunization. In particular, it was shown in an in vitro model that antibodies that bind between amino acids D671 and I721 are able to block the binding of PA to cell receptors. In the experiments with laboratory rats, it was found that the presence of antibodies only against the PA-D4 domain may be sufficient to neutralize LT and to protect against anthrax infection [17–19].

We expected to see a strong correlation between the level of antibodies to PA, LF and/or their domains and TNA, as it was shown in a number of studies on the samples of blood serum obtained from the people vaccinated against *B*. *anthrax* with chemical vaccines [8, 20]. Correlation analysis showed a moderate positive correlation between these parameters.

It was previously shown that only 24% of antibodies from the total number of PA-specific antibodies are capable of showing a neutralizing effect on PA-mediated cytotoxicity in vitro [9]. Apparently, the amount of TNA of the total number of antibodies to PA is individual for each donor. This is what causes the absence of a strong correlation between the level of antibodies to PA and protection against the infection: this was shown in many previous experiments with animals [21]. The blood must contain not just PA or LF-specific antibodies, but antibodies capable of neutralizing LT, the presence of which correlates with the protection against anthrax [22]. Our studies have shown that TNA in the samples of blood serum from vaccinated donors was detected in more than 1 year after vaccination. In 12–18 months after vaccination TNA of the blood serum from the majority of the donors was in the range 25–47%, and in two donors out of 17 analyzed ones, the blood serum was able to neutralize the cytotoxic effect of LT *in vitro* and protect more than 50% of J774.1-A cells from death.

Stepanov et al. showed that immunoglobulins synthesized as a result of vaccination with LAV strain STI-1 have not only the anti-toxic effect, but also inhibit the germination of *B*. *anthracis* spores [23]. It was later shown that after immunization with an AVA vaccine, synthesis of antibodies to PA is induced; these antibodies by opsonizing the spores of Ames virulent strain or Sterne vaccine strain contribute to the enhancement of their phagocytosis by murine peritoneal macrophages [12, 24].

The results of our studies have shown that after immunization of people with LAV antibodies against anthrax spores are synthesized and circulate in the blood of most donors during the period of 8 months after vaccination. Given the short incubation period and the rapid progression of anthrax in an aerosol or gastrointestinal forms, antibodies against surface antigens of the spores will be the most effective barrier even at the early stages of infection. This was confirmed in a mouse model with the use of monoclonal antibodies against *B*. *anthracis* spores. Monoclonal antibodies prevented spore germination into the vegetative cells in vitro, enhanced the spore phagocytosis and the death of the vegetative cells in macrophages, that resulted in 90% survival of mice that were infected with *B*. *anthracis* Ames strain [25]. These observations suggested that the immune response resulting from such vaccination can prevent the lethal intoxication, as well as fight against anthrax infection even at the initial stage of infecting with the pathogen [12].

In connection with the well-known fact that vaccination of elderly people induces a lower level of antibody synthesis compared to the vaccination of young people [26–28] we analyzed the effect of age on the level of post-vaccination immune responses. The analysis of the obtained data did not show any correlation between the age of the donors and the development and duration of immune response to immunization with LAV, as well as the duration of the circulation of IgG specific to antibodies of the causative agent of anthrax toxin, and their TNA levels of the blood serum. This can be explained by the fact that in our studies the age of the elder group of donors ranged from 40 to 60 years. Most studies have shown that a significant decrease in the effectiveness of the development of immune responses is observed in groups of people over 65 [29, 30, 31]. Gender did not affect the level of specific post-vaccination immune responses in the donors either. It seems that we were not able to see the differences in the immune response in different age or gender groups due to the small number of donors in the groups. This number resulted from the limited number of people vaccinated against LAV that live in anthrax-endemic regions.

A comparative analysis of indicators between the groups of donors with different numbers of vaccinations showed that the donors vaccinated many times (more than 10 annual immunizations with LAV) have a longer presence of TNA of the serum after the administration of the next vaccination.

One of the major directions in developing subunit anthrax vaccines is obtaining a highly purified rPA that remains stable during its storage. In our study, we showed that after immunization with LAV, antibodies against 1 and 4 domains of PA circulate in the blood of the majority of donors for the longest time possible. This suggests that the inclusion of PA domains 1 and 4 into the new subunit vaccine is effective as they are most immunogenic.

Other studies (e.g. [32]) have shown that low titers of antibodies to toxin components are observed in the blood samples of healthy unvaccinated donors who have not previously had anthrax. Moreover, background ELISA titers can be observed in the blood samples of people that live in both non-endemic and endemic areas for anthrax and who have never been vaccinated against anthrax and did not get sick with it. Typically, people who live in endemic areas have a higher immune response to the components of the *B*. *anthracis* toxin than healthy people living in non-endemic areas. However, in any case these indicators are significantly lower than the titers of specific antibodies in the blood samples of vaccinated or recovered donors.

The control group of unvaccinated donors described in our study are healthy people living in an anthrax-endemic region (in the south of Moscow region) who do not work with bacterial pathogens, do not have access to buildings where microbiological laboratory studies are carried out, and who were not vaccinated against anthrax. We cannot assert whether the titers to anthrax pathogen spores in the samples of their blood are specific or not. But we accept the data from the control group of donors as a background for comparison with the data from the group of vaccinated donors who live in the same anthrax-endemic area.

Antibody titers to PA, LF, and spores in the blood samples in the group of vaccinated donors were not very high. It may be partially connected with the high background data obtained from control donors.

On the other hand, vaccines differ in how they stimulate the immune system. Live anthrax vaccine provides a broader humoral immune response to *B*. *anthracis* antigens than AVP or AVA vaccines. But the antibody titer against PA after LAAV vaccination is lower compared to the blood samples in the group of donors vaccinated with AVP or AVA [33].

The majority of unvaccinated donors have statistically insignificant IgG titers to PA, LF and spores. IgG titers to PA and spores were determined in 71% of the blood samples in the control group of donors, IgG titers to LF were determined in 62% of the blood samples. At the same time the presence of IgG antibodies to one of the listed antigens does not necessarily mean the presence of antibodies to the rest of them. Thus, antibodies to all three components (PA, LF, and spores) were detected only in 7 blood samples out of 21, and antibodies to two components were detected in 9 out of 21 blood samples (data in S24 Dataset).

## Materials and methods

### Ethic statement

Each human volunteer provided a written informed consent for blood donation. Date of birth, gender, number of previous vaccination against anthrax were recorded. This study was approved by the Local Bioethics Committee of the Federal State Budget Institution of Science "Kirov Research Institute of Hematology and Blood Transfusion of the Federal Biomedical Agency" (Ethics committee approval number 11 of December 11^th^, 2018).

### Information about the groups of donors

The recruitment date range was from January 2017 to March 2018. The inclusion criteria was: healthy men or women aged 20 to 60 years old at the time of screening who had previously been vaccinated with LAV (Table 5). All the donors provided a written informed consent for participation in the study. The exclusion criteria were: donors multiple vaccinated with live anthrax vaccine, but who missed the next vaccine administration at least once; any confirmed or suspected immunosuppressive or immunodeficient state; pregnancy or lactation during the study.

**Table 5:** Table of the donors’ age and gender.

	No. of participants		
**Total**	88		
Vaccinated	67	Male—34	20–40 years—40
		Female—33	40–60 years—27
Non-vaccinated (Control)	21	Male—10	20–40 years—10
		Female—11	40–60 years—11
**Gender (total)**			
Male	44		
Female	44		
**Age**			
**Median age (in years)**	**37**		
20–40	51		
20–40 (median age, in years)	33		
40–60	37		
40–60 (median age, in years)	50		

The group of unvaccinated control donors included in our study are healthy people living in an anthrax-endemic region (south of Moscow region), who do not work with bacterial pathogens, who do not have access to buildings where microbiological laboratory studies are carried out, and who have not been vaccinated against anthrax.

LAV is a vaccine licensed to prevent anthrax, but it is not typically available for the general public. LAV consists of lyophilized suspension of *Bacillus anthracis*

STI-1 live vaccine strain spores and 10% solution of sucrose as stabilizer. These components are dissolved in 30% solution of glycerol.

LAV vaccine is currently provided only to people who are at an increased risk of coming in contact with anthrax spores, such as certain laboratory workers, and some people who handle animals or animal products. This means that a small number of people are vaccinated against anthrax, and even fewer people are vaccinated annually for more than 10 years. In this regard, our sample is representative of the people who were vaccinated with LAV. All the participants are employees of the FBIS SRCAMB (State Research Center for Applied Microbiology & Biotechnology) that are regularly vaccinated because of occupational risk.

### Investigated samples of blood serum from the donors

People who were vaccinated with LAV many times were chosen as donors. Their last vaccination was between 1–18 months from the date of the blood sampling for the study. The serum was obtained from venous blood collected in centrifuge vacuum tubes (Vacuette, Greiner Bio-One, Austria) containing the coagulation activator. Then this venous blood in the tubes was incubated at room temperature for 30 minutes until the clot formed completely. It was centrifuged at 2000×g for 10 min, and the resulting serum was transferred into another tube. For the analysis of toxin neutralizing activity (TNA) freshly collected serum was used, if necessary, it was stored at 4°C for six days. For ELISA studies of specific antibody titers, we used freshly collected serum or aliquots of the serum that were preserved by adding of sodium aside to a final concentration of 0.01% and frozen one time to minus 80°C. The second freezing and thawing was not allowed.

### Obtaining recombinant proteins PA and LF of *B*. *anthracis* and their domains

The expression of recombinant proteins was performed from plasmid vectors pET-PA (that contain an expression cassette with the sequence of the full-length protein PA—UniProtKB #P13423 (PAG_BACAN)), pET-PA-D1 (data in S1 Fig) (I PA domain– 30–287 aa, data in S2 Fig), pET-PA-D2 (data in S3 Fig) (II PA domain– 288–516 aa, data in S4 Fig), pET-PA-D3 (data in S5 Fig) (III PA domain– 517–623 aa, data in S6 Fig), pET-PA-D4 (data in S7 Fig) (IV PA domain– 625–764 aa, data in S8 Fig), pETHIS-LF (full-size LF—UniProtKB #P15917 (LEF_BACAN)) [34], pET-LF-D1 (data in S9 Fig) (I LF domain– 34–295 aa, data in S10 Fig), pET-LF-D2.3 (data in S11 Fig) (II and III LF domains– 296–583 aa, data in S12 Fig), pET-LF-D4 (data in S13 Fig) (IV domain of LF– 584–810 aa, data in S14 Fig). All of the mentioned expression plasmids are constructed on the basis of the commercial vector pET22b (+) (Novagen) in which BglII restriction site was destroyed [34], and as a result, we obtained the plasmid pET22b (+) Bgl-.

To obtain the plasmids from which full-length recombinant PA and LF proteins were expressed, we introduced PCR fragment containing a polyhistidine tag and c-myc peptide that were added for chromatographic purification and identification of the resulting proteins into the plasmid pET22b (+) Bgl- across NdeI and BamHI restriction sites. Then, the obtained plasmid was cleaved across the BamHI and XhoI sites into which we introduced the sequences encoding the full-length PA or LF proteins cleaved at the BamHI and SalI sites. These sites were introduced into the sequences during their amplification with *B*. *anthracis* STI-1 (State Collection of Pathogenic Microorganisms and Cell Cultures, FBIS SRCAMB, Obolensk) genomic DNA.

The plasmids, from which the full-length recombinant domains of the PA and LF proteins were expressed, were obtained in a similar way. The poly-His tag in these plasmids was replaced by a fragment of glutathione S-transferase (GST) that allowed for chromatographic purification of the proteins on a sorbent with glutathione, as well as for increase of the mass of synthesized protein domains in order to facilitate the work with them.

All the enzymes that were used are from Fermentas, USA.

Competent cells of the strain *E*. *coli* BL21(DE3) (NEB) were transformed by the obtained plasmid constructs using the electroporation method and so the producer- strains of *E*. *coli* BL-PA, *E*. *coli* BL-LF, *E*. *coli* BL-PA-D1, *E*. *coli* BL-PA -D2, *E*. *coli* BL-PA-D3, *E*. *coli* BL-PA-D4, *E*. *coli* BL-LF-D1, *E*. *coli* BL-LF-D2.3 and E. coli BL-LF-D4 were obtained.

Bacterial expression was carried out in the fermentation mode in a volume of 2xYT 10 L nutrient medium containing a selective antibiotic at a concentration of 100 μg/ml of the medium and 0.5% glucose. The culture was incubated at 37°C until glucose production was complete (after 5–6 hours of incubation the culture density is achieved up to OD600 = 7–9 units; the medium stops acidifying, the pH value reaches a plateau), then the entire volume of the culture was cooled up to 30°C, after that the expression was induced by adding IPTG to a final concentration of 1 mM. After 3 hours of cultivation with an expression inducer the biomass was separated from the culture medium by centrifugation at 7000×g for 10 minutes. The cell pellet was placed in a low-temperature refrigerator, stored at a temperature of minus 70°C until the recombinant proteins were isolated from the biomass.

All stages of the isolation of recombinant proteins were carried out while cooling on ice. The biomass collected after the expression was resuspended to homogeneity in an adequate volume of 20 mM Tris-HCl (pH 8.0), 100 mM NaCl. For lysis of bacterial cells, the cell suspension was treated with lysozyme (20 μg/ ml) for 10 minutes at 4°C. Triton X-100 was added to this suspension up to a concentration of 4%, after that this suspension was kept in the cold for at least 10 minutes. Then in order to get rid of DNA and RNA in the resulting mixture, DNase and RNase were added to a concentration of 10 μg/ml of each enzyme in the presence of 1 mm MgCl_2_ to this suspension. Then it was kept at room temperature for 15 minutes. The resulting cell lysate was cleared by centrifugation for 20 minutes at 18,000×g.

The first stage of isolation of the full-length recombinant PA and LF proteins was performed on a column clogged with His-binding sorbent cOmplete ™ His-Tag Purification Resin (Cat. No. 05 893 801 001, Roche, Switzerland). The column was balanced with 20 mM Tris-HCl buffer (pH 8.0), 100 mM NaCl, the cleared lysate was applied to the column in the same buffer, the column was washed with the application buffer until the balance was reached, and eluted with the same buffer containing 150 mM of imidazole.

To isolate the recombinant GST-containing proteins (PA and LF domains) from the cleared lysate we used a column packed by Glutathione Sepharose® 4B sorbent (Cat. No. 17-5132-02, GE Healthcare, GB) and then equilibrated with 20 mM Tris-HCl buffer (pH 8.0), 100 mM NaCl. The cleared lysate was applied to the column, the column was washed with the same buffer until the balance was reached. The elution was performed by 20 mM Tris-HCl buffer (pH 8.0), 100 mM NaCl, of 10 mM reduced glutathione.

Final purification of all recombinant proteins and their transfer to the storage buffer (20 mM Tris-HCl (pH 8.0), 100 mM NaCl) was carried out by size exclusion chromatography on Superdex 200 10/300 GL columns (full-length PA and LF proteins) (Cat. No. 17517501, GE Healthcare, GB) or Superdex 75 10/300 GL (PA and LF domains) (Cat. No. 17517401, GE Healthcare, GB). Glycerol was added to the eluted protein fraction to a volume of 30%, then we divided the obtained solution into aliquots and put for storage at a temperature of minus 70°C. The concentration of all proteins was measured using a DirectDetect infrared spectrometer (Merck Millipore, USA).

**Fig 8:**
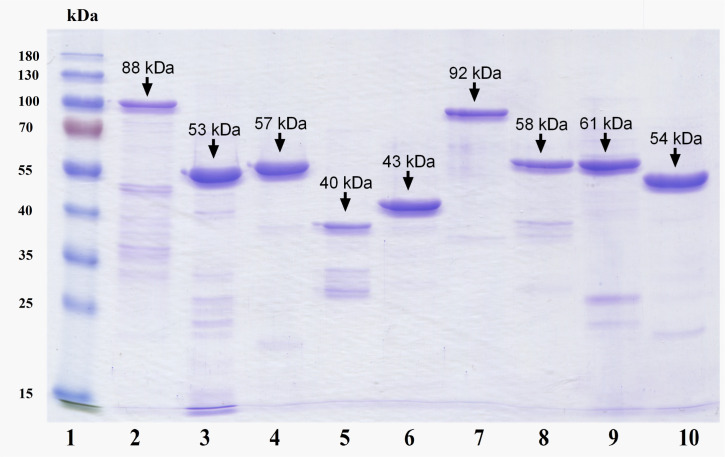
SDS-PAGE electroforesis results for recombinant proteins. 1. Commercial molecular weight marker SM0671 (Fermentas); 2. PA full-length; 3. PA-D2; 4. PA-D1; 5. PA-D3; 6. PA-D4; 7. LF full-length; 8. LF-D1; 9. LF-D2.3; 10. LF-D4.

The purity of the obtained recombinant proteins was checked by electrophoresis in SDS-PAGE under denaturing conditions using the Laemmli method [35]. The gel was stained with Coomassie brilliant blue (R-250), the molecular weight of the obtained bands was compared to a commercial molecular weight marker (Fig 8).

### Obtaining of inactivated spores of *B*. *anthracis* STI-1

Spores of *B*. *anthracis* STI-1 strain were obtained by growing microorganisms in culture bottles on Luria-Bertani agar, they were incubated at a temperature of 34±1°C until the sporulation. Sporulation was analysed on the 3^d^-10^th^ day by viewing the spores and the smears stained by Ziehl-Nielsen in the microscope. The spore concentration was detected by counting them in the Goryaev chamber. Based on the results of the calculation in the Goryaev chamber and the volume of the spore suspension, the amount of a physiological solution for diluting the bacterial suspension was calculated so that the finished spore culture contained 1×10^9^ spores/ml. Spore inactivation was carried out by adding sodium thiolate to a concentration of 0.1%. The inactivation time was 24 hours at room temperature. Inactivation control was carried out by seeding on meat-peptone agar with incubation at 37°C for 24 hours.

### Determination of antibody titers specific to the full-length PA and LF proteins of *B*. *anthracis*, their domains, and to surface spore antigens of *B*. *anthracis*, in the samples of blood serum of vaccinated donors

For the study of humoral and cellular immunity specific antigens of *B*. *anthracis* (the components of the lethal toxin and inactivated spores) were used as targets.

Determination of IgG titers in serum was performed using indirect enzyme-linked immunosorbent assay (ELISA).

#### Plate preparation, antigen sorption

A solution of one of the recombinant proteins (PA or LF *B*. *anthracis* or their domains) was added to each well of a 96-well plate in an amount of 1 μg in a volume of 100 μl in 10 mM phosphate-buffered saline (PBS), pH 7.4. The filled plate was incubated in an orbital shaker incubator for 2 hours at a temperature of 37°C with a platform rotation speed of 300 rpm. Then, all the wells of the plate were washed three times with PBS solution containing 0.05% Tween-20 (PBS-Tw). Free binding sites in the wells of the polycarbonate plate were blocked with inert proteins of skimmed milk, added in an amount of 200 μl/well. The plate was incubated for 40 min under the conditions described above. At the end of the incubation, the wells of the PBS-Tw plate were washed three times.

To perform the sorption of the inactivated *B*. *anthracis* STI-1 spores on a plate, 50 μl of a suspension containing 10^8^ spores was added to the wells. The plate was kept in a dry heat oven at a temperature of 60°С until the liquid completely evaporated. To fix the spores on the plate surface, 60 μl of 96% ethanol were added to all the wells, then the plate was dried under the same conditions. The free valences of the plate wells were blocked with skimmed milk for 40 minutes as described above, then they were washed off the milk using PBS-Tw.

All the prepared sensibilized plates were stored in a dried state with the surface sealed by a film at 4°C for a month until they were used.

#### Conducting the research

The samples of blood serum from the donors was examined in the dilutions with a double step from 1:25 to 1:3200. The dilutions in a volume of 100 μl were added to the wells of the plate with one of the target antigens in duplicate on its surface. Wells A1 and A2 with 100 μl of pure PBS were used as a negative control. The dilution of 1:500 murine polyclonal antibodies to PA, LF or *B*. *anthracis* spores previously obtained in the laboratory were used as a positive control of the plate activation. The filled plate was incubated for one hour in a shaker-incubator under the conditions described above. The wells of the plate were washed three times with PBS-Tw at the end of the incubation. To detect the binding of antibodies to the antigen, conjugates of anti-species anti-IgG antibodies with HPR: Anti-Human IgG (Fc specific) −Alkaline Phosphatase antibody produced in goat (I2136, Sigma) in dilution 1:10000 was used for the detection of the bound human immunoglobulins of the donors’ serum; and Anti-Mouse IgG (whole molecule)–Peroxidase antibody produced in rabbit (A9044, Sigma) in dilution 1:20000 was used for the detection of the bound mouse antibodies. One of two types of the conjugate was introduced into the wells of the negative control (A1 and A2) in order to control its non-specific binding to the surface of the well. Anti-mouse conjugate was added to the wells with the control of sensibilization of the plate, and the anti-human conjugate was added to the wells with the samples of blood serum. The volume of the introduced solutions was 100 μl/well, the dilution of the conjugates was 1:5000 in PBS. The plates were incubated for 1 hour at a temperature of 37°C in a shaker-incubator. After washing eight times with PBS-Tw, 100 μl of the substrate mixture which is a 0.08% solution of orthophenyl diamine in the phosphate-citrate buffer (pH 5.0) with the addition of hydrogen peroxide (37%) in a ratio of 1:1000 was added to the wells of the plate. An enzymatic reaction was carried out until the color of the solution appeared, avoiding a significant staining of the solution in the wells with negative controls. 50 μl of 4N sulfuric acid acting as a “stop reagent” was added to all the wells.

#### Registration of the results

The results were registered on Varioskan Flash spectrophotometer (Thermo Scientific, USA) at a wavelength of 492 nm. We considered the dilution with the optical density indices that exceeded by at least two times those in the wells with negative control as a limit titer. The results of the study were considered invalid if the optical density of the solution in the wells with negative control exceeded 0.15 units of the optical density, and when in the wells with positive control it was less than 1.0.

### Determination of toxin-neutralizing activity of the samples of blood serum from vaccinated donors by MTT method

#### Preparing the cell line for the experiment

The cells of the macrophage line J744A.1 (ATCC® TIB-67™) sensitive to *B*. *anthracis* LT were removed from the nitrogen storage and thawed using a water bath heated to 37°С until it was almost completely thawed, leaving a small amount of ice on the surface of the content of the vial. The cells were washed from cryopreservation medium (90% fetal bovine serum (FBS), 10% DMSO). To reach this effect, the content of the vial was placed in a centrifuge tube containing 10 ml of DMEM culture medium supplemented with 2 mM L-glutamine, a single concentration of antibiotic-antimycotic solution and 10% inactivated FBS (all Gibco, Thermo Fisher, USA) (DMEM-FBS), then it was resuspended. The cells were pelleted by centrifugation for 5 minutes at 1200 rpmin. The resulting pellet was resuspended in clean warm DMEM-FBS medium, 10 ml in volume, and placed in a T-75 culture bottle (Corning, USA) with a surface area of 75 cm^2^. The cells were incubated for three days at a temperature of 37°C, with the humidity not less than 80%, with CO_2_ content of 5%.

At the end of the incubation the attached cells monolayers were dissociated using a 0.05% trypsin-EDTA solution (Gibco, Thermo Fisher, USA) and the suspension was prepared in supplemented medium. The concentration of viable cells was counted on a TC20 Automated Cell Counter (Cat. No. 1450102, Bio-Rad) stained with 0.4% trypan blue solution (Gibco, Thermo Fisher, USA) according to the manufacturer’s method.

The J774A.1 cell suspension was added to the wells of a 96-well culture plate in an amount of 1×10^4^ viable cells/well in a total volume of 85 μl of the DMEM-FBS growth medium. The plate was placed in a CO_2_ incubator for 24 hours to adhere the cells on the surface of the wells of the plate. Then, in all the wells of the plate, the culture medium was replaced with the fresh one in a volume of 90 μl.

#### Detection of ED_100_ for *B*. *anthracis* LT

Various amounts of LT of *B*. *anthracis* from 150 ng/ml to 4800 ng/ml with a double titration step were added to the wells of the prepared plate with J744A.1 line cells; the PA/LF ratio was 5/1. Intact wells were used as negative controls, we used three types of wells: intact wells, wells with addition of only PA at a concentration of 4000 ng/ml, and wells with only LF at a concentration of 800 ng ml. Wells with the addition of sodium merthiolate (Oskar Tropitzsch, Germany) to a final concentration of 0.025% were used as a positive control. In the wells supplemented with sodium thiolate, 100% cell death is achieved. All the tests, including the control ones, were tested in four replicates. The plate was placed in a CO_2_ incubator for 4 hours. Then, a solution of 3- (4,5-dimethylthiazol-2-yl) -2,5-diphenyl-tetrazolium bromide (yellow tetrazolium, MTT) was added to all the wells. MTT stock solution was 2 mg/ml (in PBS). The final concentration of MTT reagent in the wells with the cells was 0.5 mg/ml. The plate was kept in a CO_2_ incubator for 4 hours, after that the medium in all the wells was replaced with 175 μl of DMSO (Sigma-Aldrich, USA). For full cell lysis and even dissolution of formazan crystals, the plates were placed on a TS-100C shaker platform (BioSan, Latvia) and incubated for 10 minutes at a platform rotation speed of 500 rpm at room temperature. The results were detected using an xMark Microplate Absorbance Spectrophotometer (Cat. No. 1681150, Bio-Rad, USA) at λ = 530 nm and at λ = 700 nm. The optical density was calculated by the formula: OD = A530-A700, where OD is the optical density, A530 is the value at a wavelength of 530 nm, A700 is the value at a wavelength of 700 nm.

The reference optical density values in the control wells were calculated, and then the optical density indicators in the test wells were compared with the control ones.

#### TNA study of the samples of blood serum from the donors

The samples of blood serum from the vaccinated donors were investigated in various dilutions which were 1:25, 1:50 and 1:100. Previously, the serum in the indicated dilutions were incubated for 1 hour with LT at a concentration corresponding to ED_100_ for recombinant LT, calculated before as 1000 ng/ml of PA and 200 ng/ml of LF. The dilution of the serum and LT was performed in DMEM-FBS.

All the liquid was taken away from the wells on the plate and it was replaced with a mixture of LT and one of the dilutions of the test serum in a volume of 100 μl/well. Each test dilution was added to 8 wells of a plate: 4—for testing TNA and 4—as a control for non-specific serum toxicity (K1-). 10 μl of LT stock solution containing 10,000 ng/ml PA and 2,000 ng/ml LF were added to the wells as a monitoring of the effectiveness of LT (K1 +; also 4 wells), it corresponds to the previously obtained ED_100_ value in the final dilution. Intact wells with the cells (K2) were obligatory for each plate, as well as a positive control of the cell death, into which sodium merthiolate was added up to 0.025% in a well (K2+). Further manipulations in the MTT test correspond to the process described above.

For each test well of the plate, the viability mean (in %) was calculated by the formula: OD (test)—OD (K2 +) / OD (K2 -)—OD (K2 +) × 100%, where OD (test) is the optical density value calculated as the average value for all replicates in the test wells, OD (K2 +) is the optical density value calculated as the average value for all K2+ wells, OD (K2-) is the optical density value calculated as the average value for all wells K2-.

### Statistical analysis

The obtained data were analyzed and graphically presented using GraphPad Prism 6. The ELISA and MTT test data were analyzed using the Kruskal-Wallis test with multiple Dunn’s comparisons in a One-Way ANOVA. The distribution was analysed using the Shapiro-Wilk test, p values from 0.05 and lower were considered as significant. The effect of age and gender on the development of post-vaccination immunity was analysed using a Two-way ANOVA with Tukey’s multiple comparisons. Correlation analysis was carried out using Spearman’s rank correlation test.

## Supporting information

S1 Fig An expression cassette of pET-PA-D1 vector. (PDF)

S2 Fig Amino acid sequence of the expressed protein GST-containing I PA domain protein. (PDF)

S3 Fig An expression cassette of pET-PA-D2 vector. (PDF)

S4 Fig Amino acid sequence of the expressed protein GST-containing II PA domain protein. (PDF)

S5 Fig An expression cassette of pET-PA-D3 vector. (PDF)

S6 Fig Amino acid sequence of the expressed protein GST-containing III PA domain protein. (PDF)

S7 Fig An expression cassette of pET-PA-D4 vector. (PDF)

S8 Fig Amino acid sequence of the expressed protein GST-containing IV PA domain protein. (PDF)

S9 Fig An expression cassette of pET-LF-D1 vector. (PDF)

S10 Fig Amino acid sequence of the expressed protein GST-containing I LF domain protein. (PDF)

S11 Fig An expression cassette of pET-LF-D2.3 vector. (PDF)

S12 Fig Amino acid sequence of the expressed protein GST-containing II+III LF domain protein. (PDF)

S13 Fig An expression cassette of pET-LF-D4 vector. (PDF)

S14 Fig Amino acid sequence of the expressed protein GST-containing IV LF domain protein. (PDF)

S1 Raw images (PDF)

S1 Dataset Level of specific IgG to full-length PA of *B*. *anthracis* in the samples of blood serum from the donors. (PDF)

S2 Dataset Level of specific IgG to full-length LF antigens of *B*. *anthracis* in the samples of blood serum from the donors. (PDF)

S3 Dataset Level of specific IgG to spores antigens of *B*. *anthracis* in the samples of blood serum from the donors. (PDF)

S4 Dataset Level of specific IgG to PA-D1 of *B*. *anthracis* in the samples of blood serum from the donors. (PDF)

S5 Dataset Level of specific IgG to PA-D2 of *B*. *anthracis* in the samples of blood serum from the donors. (PDF)

S6 Dataset Level of specific IgG to PA-D3 of *B*. *anthracis* in the samples of blood serum from the donors. (PDF)

S7 Dataset Level of specific IgG to PA-D4 of *B*. *anthracis* in the samples of blood serum from the donors. (PDF)

S8 Dataset Level of specific IgG to LF-D1 of *B*. *anthracis* in the samples of blood serum from the donors. (PDF)

S9 Dataset Level of specific IgG to LF-D2.3 of *B*. *anthracis* in the samples of blood serum from the donors. (PDF)

S10 Dataset Level of specific IgG to LF-D4 of *B*. *anthracis* in the samples of blood serum from the donors. (PDF)

S11 Dataset Toxin-neutralizing activity of the samples of blood serum (dilution 1:25) from the donors vaccinated against anthrax. (PDF)

S12 Dataset Correlation analysis between the toxin-neutralizing activity of the samples of blood serum from the donors and antibody titers against PA. (PDF)

S13 Dataset Correlation analysis between the toxin-neutralizing activity of the samples of blood serum from the donors and antibody titers against LF. (PDF)

S14 Dataset Correlation analysis between the toxin-neutralizing activity of the samples of blood serum from the donors and antibody titers against PA domains. (PDF)

S15 Dataset Correlation analysis between the toxin-neutralizing activity of the samples of blood serum from the donors and antibody titers against LF domains. (PDF)

S16 Dataset Analysis of the effect of age on the development and duration of anti-anthrax post-vaccination immunity (age vs. PA titers). (PDF)

S17 Dataset Analysis of the effect of age on the development and duration of anti-anthrax post-vaccination immunity (age vs. LF titers). (PDF)

S18 Dataset Analysis of the effect of age on the development and duration of anti-anthrax post-vaccination immunity (age vs. spores titers). (PDF)

S19 Dataset Analysis of the effect of age on the development and duration of anti-anthrax post-vaccination immunity (age vs. TNA). (PDF)

S20 Dataset The effect of gender on the development of anti-anthrax post-vaccination immunity and the duration of circulation of IgG to PA. (PDF)

S21 Dataset The effect of gender on the development of anti-anthrax post-vaccination immunity and the duration of circulation of IgG to LF. (PDF)

S22 Dataset The effect of gender on the development of anti-anthrax post-vaccination immunity and the duration of circulation of IgG to spores. (PDF)

S23 Dataset The effect of gender on the development of anti-anthrax post-vaccination immunity and TNA. (PDF)

S24 Dataset Level of specific IgG PA, LF of *B*. *anthracis* and domains and levels of TNA in the samples of blood serum from the donors in the control group. (PDF)

## References

[1] Chen Z, Moayeri M, Zhou Y, Leppla S, Emerson S, Sebrell A, et al. Efficient Neutralization of Anthrax Toxin by Chimpanzee Monoclonal Antibodies against Protective Antigen. J Infect Dis. 2006 Mar; 193(5): 625–33. doi: 10.1086/500148 16453257 PMC7110013

[2] Brossier F, Lévy M, Landier A, Lafaye P, Mock M. Functional Analysis of *Bacillus anthracis* Protective Antigen by Using Neutralizing Monoclonal Antibodies. Infect Immun. 2004 Nov;72(11): 6313–7. doi: 10.1128/IAI.72.11.6313-6317.2004 15501759 PMC523002

[3] Sawada-Hirai R, Jiang I, Wang F, Sun S, Nedellec R, Ruther P, et al. Human anti-anthrax protective antigen neutralizing monoclonal antibodies derived from donors vaccinated with anthrax vaccine adsorbed. J Immune Based Ther Vaccines. 2004;2(1): 5. doi: 10.1186/1476-8518-2-5 15140257 PMC420254

[4] Lim N-K, Kim J-H, Oh MS, Lee S, Kim S-Y, Kim K-S, et al. An Anthrax Lethal Factor-Neutralizing Monoclonal Antibody Protects Rats before and after Challenge with Anthrax Toxin. Infect Immun. 2005 Oct;73(10): 6547–51. doi: 10.1128/IAI.73.10.6547-6551.2005 16177329 PMC1230968

[5] Maynard JA, Maassen CBM, Leppla SH, Brasky K, Patterson JL, Iverson BL, et al. Protection against anthrax toxin by recombinant antibody fragments correlates with antigen affinity. Nat Biotechnol. 2002 Jun;20(6): 597–601. doi: 10.1038/nbt0602-597 12042864

[6] Leppla SH. Anthrax Toxin. In: Bacterial Protein Toxins. Springer Berlin Heidelberg; 2000. p. 445–72. doi: 10.1007/978-3-662-05971-5_19

[7] Liu S, Moayeri M, Leppla SH. Anthrax lethal and edema toxins in anthrax pathogenesis. Trends Microbiol. 2014 Jun;22(6): 317–25. doi: 10.1016/j.tim.2014.02.012 24684968 PMC4041834

[8] Crowe SR, Ash LL, Engler RJM, Ballard JD, Harley JB, Farris AD, et al. Select Human Anthrax Protective Antigen Epitope‐Specific Antibodies Provide Protection from Lethal Toxin Challenge. J Infect Dis. 2010 Jul 15;202(2):251–60. doi: 10.1086/653495 20533877 PMC2891133

[9] Reason D, Liberato J, Sun J, Keitel W, Zhou J. Frequency and Domain Specificity of Toxin-Neutralizing Paratopes in the Human Antibody Response to Anthrax Vaccine Adsorbed. IAI. 2009 Feb 17; 77(5):2030–5. doi: 10.1128/IAI.01254-08 19223482 PMC2681729

[10] Laws TR, Kuchuloria T, Chitadze N, Little SF, Webster W, Debes AK et al. A Comparison of the Adaptive Immune Response between Recovered Anthrax Patients and Individuals Receiving Three Different Anthrax Vaccines. PLOS ONE. 2016 Mar 23;11(3): e0148713. doi: 10.1371/journal.pone.0148713 27007118 PMC4805272

[11] Ionin B, Hopkins RJ, Pleune B, Sivko GS, Reid FM, Clement KH et al. Evaluation of Immunogenicity and Efficacy of Anthrax Vaccine Adsorbed for Postexposure Prophylaxis. Clin Vaccine Immunol. 2013 Jul;20(7):1016–1026. doi: 10.1128/CVI.00099-13 23658392 PMC3697458

[12] Welkos S, Little S, Friedlander A, Fritz D, Fellows P. The role of antibodies to *Bacillus anthracis* and anthrax toxin components in inhibiting the early stages of infection by anthrax spores. Microbiol. 2001 Jun 1;147(6): 1677–85. doi: 10.1099/00221287-147-6-1677 11390699

[13] Welkos S. Friedlander A. Weeks S. Little S. Mendelson I. In-vitro characterisation of the phagocytosis and fate of anthrax spores in macrophages and the effects of anti-PA antibody. J Med Microbiol. 2002 Oct 1;51(10): 821–31. doi: 10.1099/0022-1317-51-10-821 12435060

[14] Abboud N, De Jesus M, Nakouzi A, Cordero RJB, Pujato M, Fiser A, et al. Identification of linear epitopes in *Bacillus anthracis* protective antigen bound by neutralizing antibodies. J Biol Chem. 2009;284: 25077–86. doi: 10.1074/jbc.M109.022061 19617628 PMC2757211

[15] Reason D, Liberato J, Sun J, Camacho J, Zhou J. Mechanism of Lethal Toxin Neutralization by a Human Monoclonal Antibody Specific for the PA20 Region of *Bacillus anthracis* Protective Antigen. Toxins. 2011 Aug 9;3(8): 979–90. doi: 10.3390/toxins3080979 22069752 PMC3202870

[16] Cunningham K, Lacy DB, Mogridge J, Collier RJ. Mapping the lethal factor and edema factor binding sites on oligomeric anthrax protective antigen. Proc Natl Acad Sci USA. 2002 May 7;99(10): 7049–53. doi: 10.1073/pnas.062160399 11997439 PMC124526

[17] Rosovitz MJ, Schuck P, Varughese M, Chopra AP, Mehra V, Singh Y, et al. Alanine-scanning Mutations in Domain 4 of Anthrax Toxin Protective Antigen Reveal Residues Important for Binding to the Cellular Receptor and to a Neutralizing Monoclonal Antibody. J Biol Chem. 2003 May 27;278(33): 30936–44. doi: 10.1074/jbc.M301154200 12771151

[18] Little SF, Leppla SH, Cora E. Production and characterization of monoclonal antibodies to the protective antigen component of *Bacillus anthracis* toxin. Infect Immun. 1988;56(7): 1807–13. doi: 10.1128/iai.56.7.1807-1813.1988 3384478 PMC259481

[19] Ahn B-E, Bae H-W, Lee H-R, Woo S-J, Park O-K, Jeon JH, et al. A therapeutic human antibody against the domain 4 of the *Bacillus anthracis* protective antigen shows protective efficacy in a mouse model. Biochem Biophys Res Commun. 2019 Feb;509(2): 611–6. doi: 10.1016/j.bbrc.2018.12.146 30606479

[20] Crowe SR, Garman L, Engler RJM, Farris AD, Ballard JD, Harley JB, et al. Anthrax vaccination induced anti-lethal factor IgG: Fine specificity and neutralizing capacity. Vaccine. 2011 May;29(20):3670–8. doi: 10.1016/j.vaccine.2011.03.011 21420416 PMC3233230

[21] Reuveny S, White MD, Adar YY, Kafri Y, Altboum Z, Gozes Y, et al. Search for Correlates of Protective Immunity Conferred by Anthrax Vaccine. Burns DL, editor. Infect Immun. 2001 May 1;69(5):2888–93. doi: 10.1128/IAI.69.5.2888-2893.2001 11292703 PMC98239

[22] Savransky V, Shearer JD, Gainey MR, Sanford DC, Sivko GS, Stark GV, et al. Correlation between anthrax lethal toxin neutralizing antibody levels and survival in guinea pigs and nonhuman primates vaccinated with the AV7909 anthrax vaccine candidate. Vaccine. 2017 Sep;35(37):4952–9. doi: 10.1016/j.vaccine.2017.07.076 28774566 PMC5580334

[23] Stepanov AV, Marinin LI, Pomerantsev AP, Staritsin NA. Development of novel vaccines against anthrax in man. J Biotechnol. 1996 Jan;44(1–3): 155–60. doi: 10.1016/0168-1656(95)00092-5 8717399

[24] Cote CK, Rossi CA, Kang AS, Morrow PR, Lee JS, Welkos SL. The detection of protective antigen (PA) associated with spores of *Bacillus anthracis* and the effects of anti-PA antibodies on spore germination and macrophage interactions. Microb Pathog. 2005 May;38(5–6): 209–25. doi: 10.1016/j.micpath.2005.02.001 15925272

[25] Majumder S, Das S, Kingston J, Shivakiran MS, Batra HV, Somani VK, et al. Functional characterization and evaluation of protective efficacy of EA752–862 monoclonal antibody against *B*. *anthracis* vegetative cell and spores. Med Microbiol Immunol. 2019 Dec 6;209(2): 125–37. doi: 10.1007/s00430-019-00650-5 31811379

[26] Frasca D, Diaz A, Romero M, Landin AM, Blomberg BB. Age effects on B cells and humoral immunity in humans. Ageing Res Rev. 2011 Jul;10(3): 330–5. doi: 10.1016/j.arr.2010.08.004 20728581 PMC3040253

[27] Frasca D, Landin AM, Alvarez JP, Blackshear PJ, Riley RL, Blomberg BB. Tristetraprolin, a Negative Regulator of mRNA Stability, Is Increased in Old B Cells and Is Involved in the Degradation of E47 mRNA. J Immunol. 2007 Jul 3;179(2): 918–27. doi: 10.4049/jimmunol.179.2.918 17617583

[28] Saurwein-Teissl M, Lung TL, Marx F, Gschösser C, Asch E, Blasko I, et al. Lack of Antibody Production Following Immunization in Old Age: Association with CD8+CD28− T Cell Clonal Expansions and an Imbalance in the Production of Th1 and Th2 Cytokines. J Immunol. 2002 Jun 1;168(11): 5893–9. doi: 10.4049/jimmunol.168.11.5893 12023394

[29] Chong Y, Ikematsu H, Yamaji K, Nishimura M, Nabeshima S, Kashiwagi S, et al. CD27+ (memory) B cell decrease and apoptosis-resistant CD27- (naive) B cell increase in aged humans: implications for age-related peripheral B cell developmental disturbances. Int Immunol. 2005 Feb 14;17(4): 383–90. doi: 10.1093/intimm/dxh218 15724062

[30] Colonna-Romano G, Bulati M, Aquino A, Scialabba G, Candore G, Lio D, et al. B cells in the aged: CD27, CD5, and CD40 expression. Mech Ageing Dev. 2003 Apr;124(4): 389–93. doi: 10.1016/s0047-6374(03)00013-7 12714244

[31] Van der Heiden M, Boots AMH, Bonacic Marinovic AA, de Rond LGH, van Maurik M, Tcherniaeva I, et al. Novel Intervention in the Aging Population: A Primary Meningococcal Vaccine Inducing Protective IgM Responses in Middle-Aged Adults. Front Immunol. 2017 Jul 19;8. doi: 10.3389/fimmu.2017.00817 28769927 PMC5515833

[32] Ghosh N, Goel AK. Anti-protective antigen IgG enzyme-linked immunosorbent assay for diagnosis of cutaneous anthrax in India. Clinical and Vaccine Immunology. 2012; 19(8): 1238–1242. doi: 10.1128/CVI.00154-12 22718130 PMC3416091

[33] Laws TR, Kuchuloria T, Chitadze N, Little SF, Webster WM, Debes AK, et al. (2016) A Comparison of the Adaptive Immune Response between Recovered Anthrax Patients and Individuals Receiving Three Different Anthrax Vaccines. PLoS ONE 11(3): e0148713. doi: 10.1371/journal.pone.0148713 27007118 PMC4805272

[34] Kolesnikov AV, Zakharova MJu, Kozyr AV, Shemjakin IG, inventors; Shemyakin-Ovchinnikov Institute of Bioorganic Chemistry, assignee. Method of obtaining functionally active recombinant protein of anthrax lethal factor (LF), recombinant plasmid pETHis-LF coding active LF protein and *Escherichia coli* BL-HisLF strain producing active protein of anthrax lethal factor. Russian Federation patent RU 2361921. 2007 Nov 23.

[35] Laemmli UK. Cleavage of Structural Proteins during the Assembly of the Head of Bacteriophage T4. Nature. 1970 Aug;227(5259): 680–5. doi: 10.1038/227680a0 5432063

