# Supplementary material for: Characterization of the adaptive immune response of donors receiving live anthrax vaccine
Source: PLoS One. 2021 Dec 20;16(12):e0260202. doi: 10.1371/journal.pone.0260202 (PMC8687594; doi:10.1371/journal.pone.0260202)
Supplement: S4 Fig — (PDF) [file pone.0260202.s004.pdf]

MSPILGYWKIKGLVQPTRLLLEYLEEKYEEHLYERDEGDKWRNKKFELGLEFPNLPYYIDGDVKLTQSMA
IIRYIADKHNMLGGCPKERAEISMLEGAVLDIRYGVSRIAYSKDFETLKVDFLSKLPEMLKMFEDRLCHK
TYLNGDHVTHPDFMLYDALDVVLYMDPMCLDAFPKLVCFKKRIEAIPQIDKYLKSSKYIAWPLQGWQATF
GGGDHPPKSGEDLEQKLISEEDLEDPYPIVHVDMENIILSKNEDQSTQNTDSQTRTISKNTSTSRTHTSE
VHGNAEVHASFFDIGGSVSAGFSNSNSSTVAIDHSLSLAGERTWAETMGLNTADTARLNANIRYVNTGTA
PIYNVLPTTSLVLGKNQTLATIKAKENQLSQILAPNNYYPSKNLAPIALNAQDDFSSTPITMNYNQFLEL
EKTKQLRLDTDQVYGNIATYNFENGRVRVDTGSNWSEVLPQIQET

S4 Fig. Amino acid sequence of the expressed protein GST-containing II PA domain protein. Colours: magenta – GST protein, cyan - c-Myc peptide, yellow - II PA domain polypeptide.
