# Supplementary material for: Characterization of the adaptive immune response of donors receiving live anthrax vaccine
Source: PLoS One. 2021 Dec 20;16(12):e0260202. doi: 10.1371/journal.pone.0260202 (PMC8687594; doi:10.1371/journal.pone.0260202)
Supplement: S7 Fig — (PDF) [file pone.0260202.s007.pdf]

TCTCGATCCCGCGAAATTAATACGACTCACTATAGGGGAATTGTGAGCGGATAACAATTCCCCTCTAGAA
ATAATTTTGTTTAACTTTAAGAAGGAGATATACATATGTCCCCTATACTAGGTTATTGGAAAATTAAGGG
CCTTGTGCAACCCACTCGACTTCTTTTGGAATATCTTGAAGAAAAATATGAAGAGCATTTGTATGAGCGC
GATGAAGGTGATAAATGGCGAAACAAAAAGTTTGAATTGGGTTTGGAGTTTCCCAATCTTCCTTATTATA
TTGATGGTGATGTTAAATTAACACAGTCTATGGCCATCATACGTTATATAGCTGACAAGCACAACATGTT
GGGTGGTTGTCCAAAAGAGCGTGCAGAGATTTCAATGCTTGAAGGAGCGGTTTTGGATATTAGATACGGT
GTTTCGAGAATTGCATATAGTAAAGACTTTGAAACTCTCAAAGTTGATTTTCTTAGCAAGCTACCTGAAA
TGCTGAAAATGTTCGAAGATCGTTTATGTCATAAAACATATTTAAATGGTGATCATGTAACCCATCCTGA
CTTCATGTTGTATGACGCTCTTGATGTTGTTTTATACATGGACCCAATGTGCCTGGATGCGTTCCCAAAA
TTAGTTTGTTTTAAAAAACGTATTGAAGCTATCCCACAAATTGATAAGTACTTGAAATCCAGCAAGTATA
TAGCATGGCCTTTGCAGGGCTGGCAAGCCACGTTTGGTGGTGGCGACCATCCTCCGAAATCTGGCGAAGA
TCTGGAACAGAAGCTTATCTCCGAAGAGGACCTGGAGGATCCGTTTCATTATGATAGAAATAACATAGCA
GTTGGGGCGGATGAGTCAGTAGTTAAGGAGGCTCATAGAGAAGTAATTAATTCGTCAACAGAGGGATTAT
TGTTAAATATTGATAAGGATATAAGAAAAATATTATCAGGTTATATTGTAGAAATTGAAGATACTGAAGG
GCTTAAAGAAGTTATAAATGACAGATATGATATGTTGAATATTTCTAGTTTACGGCAAGATGGAAAAACA
TTTATAGATTTTAAAAAATATAATGATAAATTACCGTTATATATAAGTAATCCCAATTATAAGGTAAATG
TATATGCTGTTACTAAAGAAAACACTATTATTAATCCTAGTGAGAATGGGGATACTAGTACCAACGGGAT
CAAGAAAATTTTAATCTTTTCTAAAAAAGGCTATGAGATAGGATAACTCGAGCACCACCACCACCACCAC
TGAGATCCGGCTGCTAACAAAGCCCGAA

S7 Fig. An expression cassette of pET-PA-D4 vector. Colours: magenta – GST protein, cyan - c-Myc peptide, yellow - IV PA domain polypeptide.
