# Supplementary material for: Characterization of the adaptive immune response of donors receiving live anthrax vaccine
Source: PLoS One. 2021 Dec 20;16(12):e0260202. doi: 10.1371/journal.pone.0260202 (PMC8687594; doi:10.1371/journal.pone.0260202)
Supplement: S9 Fig — (PDF) [file pone.0260202.s009.pdf]

TCTCGATCCCGCGAAATTAATACGACTCACTATAGGGGAATTGTGAGCGGATAACAATTCCCCTCTAGAA
ATAATTTTGTTTAACTTTAAGAAGGAGATATACATATGTCCCCTATACTAGGTTATTGGAAAATTAAGGG
CCTTGTGCAACCCACTCGACTTCTTTTGGAATATCTTGAAGAAAAATATGAAGAGCATTTGTATGAGCGC
GATGAAGGTGATAAATGGCGAAACAAAAAGTTTGAATTGGGTTTGGAGTTTCCCAATCTTCCTTATTATA
TTGATGGTGATGTTAAATTAACACAGTCTATGGCCATCATACGTTATATAGCTGACAAGCACAACATGTT
GGGTGGTTGTCCAAAAGAGCGTGCAGAGATTTCAATGCTTGAAGGAGCGGTTTTGGATATTAGATACGGT
GTTTCGAGAATTGCATATAGTAAAGACTTTGAAACTCTCAAAGTTGATTTTCTTAGCAAGCTACCTGAAA
TGCTGAAAATGTTCGAAGATCGTTTATGTCATAAAACATATTTAAATGGTGATCATGTAACCCATCCTGA
CTTCATGTTGTATGACGCTCTTGATGTTGTTTTATACATGGACCCAATGTGCCTGGATGCGTTCCCAAAA
TTAGTTTGTTTTAAAAAACGTATTGAAGCTATCCCACAAATTGATAAGTACTTGAAATCCAGCAAGTATA
TAGCATGGCCTTTGCAGGGCTGGCAAGCCACGTTTGGTGGTGGCGACCATCCTCCGAAATCTGGCGAAGA
TCTGGAACAGAAGCTTATCTCCGAAGAGGACCTGGAGGATCCGGCGGGCGGCCATGGCGATGTGGGCATG
CATGTGAAAGAAAAAGAAAAAAACAAAGATGAAAACAAACGCAAAGATGAAGAACGCAACAAAACCCAGG
AAGAACATCTGAAAGAAATTATGAAACATATTGTGAAAATTGAAGTGAAAGGCGAAGAAGCGGTGAAAAA
AGAAGCGGCGGAAAAACTGCTGGAAAAAGTGCCGAGCGATGTGCTGGAAATGTATAAAGCGATTGGCGGC
AAAATTTATATTGTGGATGGCGATATTACCAAACATATTAGCCTGGAAGCGCTGAGCGAAGATAAAAAAA
AAATTAAAGATATTTATGGCAAAGATGCGCTGCTGCATGAACATTATGTGTATGCGAAAGAAGGCTATGA
ACCGGTGCTGGTGATTCAGAGCAGCGAAGATTATGTGGAAAACACCGAAAAAGCGCTGAACGTGTATTAT
GAAATTGGCAAAATTCTGAGCCGCGATATTCTGAGCAAAATTAACCAGCCGTATCAGAAATTTCTGGATG
TGCTGAACACCATTAAAAACGCGAGCGATAGCGATGGCCAGGATCTGCTGTTTACCAACCAGCTGAAAGA
ACATCCGACCGATTTTAGCGTGGAATTTCTGGAACAGAACAGCAACGAAGTGCAGGAAGTGTTTGCGAAA
GCGTTTGCGTATTATATTGAACCGCAGCATCGCGATGTGCTGCAGCTGTATGCGCCGGAAGCGTTTAACT
ATATGGATAAATTTAACGAACAGGAAATTAACCTGAGCCTGGAAGAACTGAAAGATCAGTAACTCGAGCA
CCACCACCACCACCACTGAGATCCGGCTGCTAACAAAGCCCGAA

S9 Fig. An expression cassette of pET-LF-D1 vector. Colours: magenta – GST protein, cyan - c-Myc peptide, yellow - I LF domain polypeptide.
