# Supplementary material for: Characterization of the adaptive immune response of donors receiving live anthrax vaccine
Source: PLoS One. 2021 Dec 20;16(12):e0260202. doi: 10.1371/journal.pone.0260202 (PMC8687594; doi:10.1371/journal.pone.0260202)
Supplement: S11 Fig — (PDF) [file pone.0260202.s011.pdf]

TCTCGATCCCGCGAAATTAATACGACTCACTATAGGGGAATTGTGAGCGGATAACAATTCCCCTCTAGAA
ATAATTTTGTTTAACTTTAAGAAGGAGATATACATATGTCCCCTATACTAGGTTATTGGAAAATTAAGGG
CCTTGTGCAACCCACTCGACTTCTTTTGGAATATCTTGAAGAAAAATATGAAGAGCATTTGTATGAGCGC
GATGAAGGTGATAAATGGCGAAACAAAAAGTTTGAATTGGGTTTGGAGTTTCCCAATCTTCCTTATTATA
TTGATGGTGATGTTAAATTAACACAGTCTATGGCCATCATACGTTATATAGCTGACAAGCACAACATGTT
GGGTGGTTGTCCAAAAGAGCGTGCAGAGATTTCAATGCTTGAAGGAGCGGTTTTGGATATTAGATACGGT
GTTTCGAGAATTGCATATAGTAAAGACTTTGAAACTCTCAAAGTTGATTTTCTTAGCAAGCTACCTGAAA
TGCTGAAAATGTTCGAAGATCGTTTATGTCATAAAACATATTTAAATGGTGATCATGTAACCCATCCTGA
CTTCATGTTGTATGACGCTCTTGATGTTGTTTTATACATGGACCCAATGTGCCTGGATGCGTTCCCAAAA
TTAGTTTGTTTTAAAAAACGTATTGAAGCTATCCCACAAATTGATAAGTACTTGAAATCCAGCAAGTATA
TAGCATGGCCTTTGCAGGGCTGGCAAGCCACGTTTGGTGGTGGCGACCATCCTCCGAAATCTGGCGAAGA
TCTGGAACAGAAGCTTATCTCCGAAGAGGACCTGGAGGATCCGCGGATGCTGGCAAGATATGAAAAATGG
GAAAAGATAAAACAGCACTATCAACACTGGAGCGATTCTTTATCTGAAGAAGGAAGAGGACTTTTAAAAA
AGCTGCAGATTCCTATTGAGCCAAAGAAAGATGACATAATTCATTCTTTATCTCAAGAAGAAAAAGAGCT
TCTAAAAAGAATACAAATTGATAGTAGTGATTTTTTATCTACTGAGGAAAAAGAGTTTTTAAAAAAGCTA
CAAATTGATATTCGTGATTCTTTATCTGAAGAAGAAAAAGAGCTTTTAAATAGAATACAGGTGGATAGTA
GTAATCCTTTATCTGAAAAAGAAAAAGAGTTTTTAAAAAAGCTGAAACTTGATATTCAACCATATGATAT
TAATCAAAGGTTGCAAGATACAGGAGGGTTAATTGATAGTCCGTCAATTAATCTTGATGTAAGAAAGCAG
TATAAAAGGGATATTCAAAATATTGATGCTTTATTACATCAATCCATTGGAAGTACCTTGTACAATAAAA
TTTATTTGTATGAAAATATGAATATCAATAACCTTACAGCAACCCTAGGTGCGGATTTAGTTGATTCCAC
TGATAATACTAAAATTAATAGAGGTATTTTCAATGAATTCAAAAAAAATTTCAAATATAGTATTTCTAGT
AACTATATGATTGTTGATATAAATGAAAGGCCTGCATTAGATAATGAGCGTTTGAAATGGAGAATCCAAT
TATCACCAGATACTCGAGCAGGATATTTAGAAAATGGAAAGCTTATATTACAAAGAAACATCGGTCTGGA
AATAAAGGATGTACAAATAATTAAGCAATCCGAAAAAGAATATATAAGGATTGATGCGAAAGTAGTGCCT
AACTCGAGCACCACCACCACCACCACTGAGATCCGGCTGCTAACAAAGCCCGAA

S11 Fig. An expression cassette of pET-LF-D2.3 vector. Colours: magenta – GST protein, cyan - c-Myc peptide, yellow – II+III LF domain polypeptide.
