# Supplementary figures and images for: Characterization of the adaptive immune response of donors receiving live anthrax vaccine
Source: PLoS One. 2021 Dec 20;16(12):e0260202. doi: 10.1371/journal.pone.0260202 (PMC8687594; doi:10.1371/journal.pone.0260202)

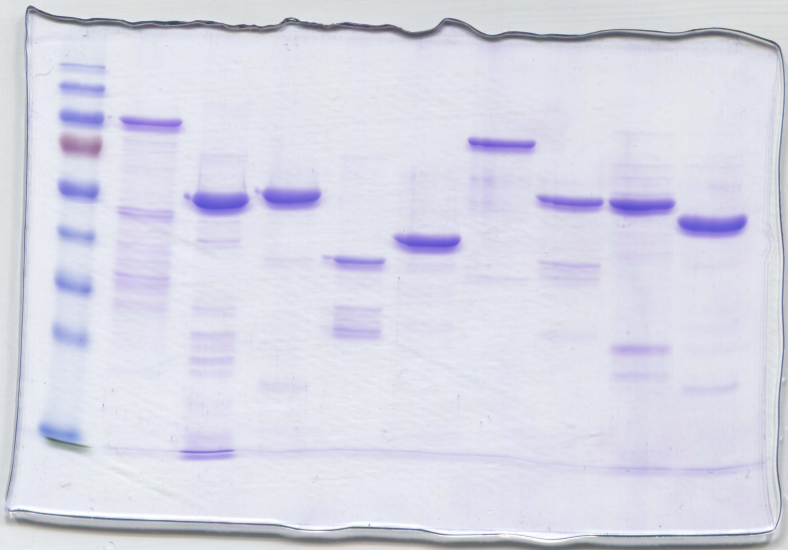

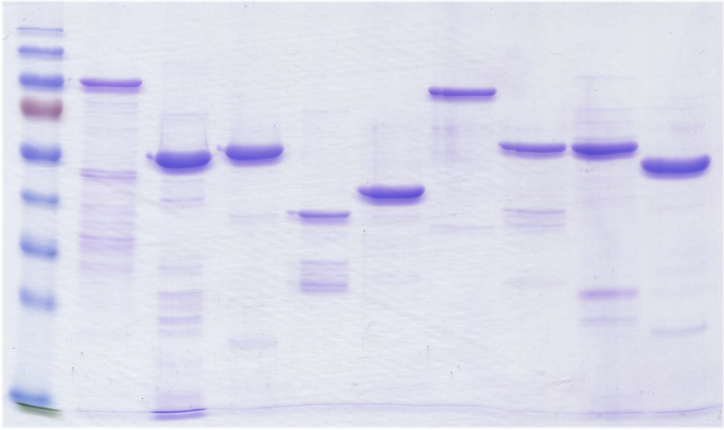

**kDa**

180  
130  
100  
70  
55  
40  
35  
25  
15

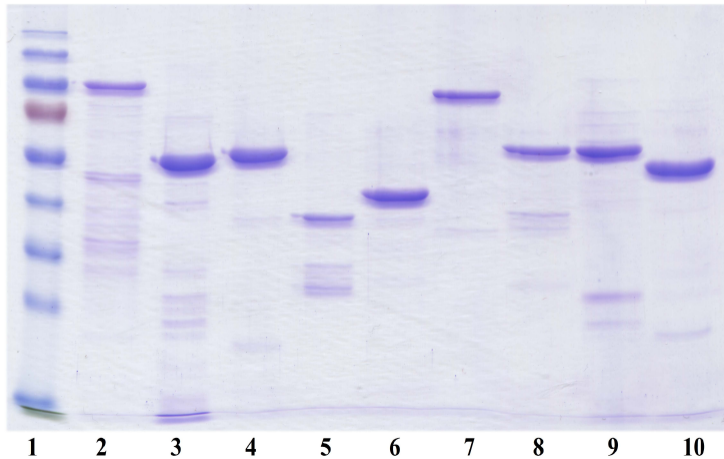

Supplement: S1 Raw images — (PDF) [file pone.0260202.s015.pdf]
