# Supplementary material for: Characterization of the adaptive immune response of donors receiving live anthrax vaccine
Source: PLoS One. 2021 Dec 20;16(12):e0260202. doi: 10.1371/journal.pone.0260202 (PMC8687594; doi:10.1371/journal.pone.0260202)

Level of specific IgG to full-length PA of *B. anthracis* in the samples of blood serum from the donors.

The data are presented by a median titer with an interquartile range as a characteristic of the spread of values in the groups. The distribution was analysed using the Shapiro-Wilk test. The data were analysed using the Kruskal-Wallis test with multiple Dunn's comparisons in a One-Way ANOVA.

	**Months after Vaccination**				
	**1-3**	**4-8**	**9-11**	**>12**	**Nonvaccinated**
**Titers**	200	800	0	50	100
	800	400	400	25	0
	100	400	50	100	0
	800	100	100	100	400
	800	0	1600	100	200
	100	25	200	200	50
	1600	400	200	100	200
	1600	800	100	50	400
	800	400	0	50	0
	800	25	50	400	50
	400	100	50	25	400
	100	25	25	200	100
	800	50	100	0	0
	800	100	400	0	25
	400	25	200	0	400
	400	400		25	50
		0		100	0
		100			100
		100			50
					25
					200

**One-Way ANOVA**	
**Table Analyzed**	**PA full-length**
Kruskal-Wallis test	
P value	< 0,0001
Exact or approximate P value?	Approximate
P value summary	****
Do the medians vary signif. (P < 0.05)	Yes
Number of groups	5
Kruskal-Wallis statistic	23,73
Data summary	
Number of treatments (columns)	5

**ANOVA Multiple Comparison**					
Number of families	1				
Number of comparisons per family	10				
Alpha	0,05				
Dunn's multiple comparisons test	Mean rank diff,	Significant?	Summary		
1-3 vs. 4-8	27,03	Yes	*		
1-3 vs. 9-11	28,24	Yes	*		
1-3 vs. >12	38,29	Yes	***		
1-3 vs. Nonvaccinated	34,9	Yes	***		
4-8 vs. 9-11	1,209	No	ns		
4-8 vs. >12	11,25	No	ns		
4-8 vs. Nonvaccinated	7,866	No	ns		
9-11 vs. >12	10,05	No	ns		
9-11 vs. Nonvaccinated	6,657	No	ns		
>12 vs. Nonvaccinated	-3,388	No	ns		
Test details	Mean rank 1	Mean rank 2	Mean rank diff,	n1	n2
1-3 vs. 4-8	70,88	43,84	27,03	16	19
1-3 vs. 9-11	70,88	42,63	28,24	16	15
1-3 vs. >12	70,88	32,59	38,29	16	17
1-3 vs. Nonvaccinated	70,88	35,98	34,9	16	21
4-8 vs. 9-11	43,84	42,63	1,209	19	15
4-8 vs. >12	43,84	32,59	11,25	19	17
4-8 vs. Nonvaccinated	43,84	35,98	7,866	19	21
9-11 vs. >12	42,63	32,59	10,05	15	17
9-11 vs. Nonvaccinated	42,63	35,98	6,657	15	21
>12 vs. Nonvaccinated	32,59	35,98	-3,388	17	21

**Descriptive Statistics**					
	Months after vaccination				
	1-3	4-8	9-11	>12	Nonvaccinated
Number of values	16	19	15	17	21
Minimum	100	0	0	0	0
25% Percentile	250	25	50	25	12,5
Median	800	100	100	50	50
75% Percentile	800	400	200	100	200
Maximum	1600	800	1600	400	400
Mean	656,3	223,7	231,7	89,71	131
Std. Deviation	466,1	256,5	399,4	100,8	148,5
Std. Error of Mean	116,5	58,85	103,1	24,45	32,41
Lower 95% CI	407,9	100,1	10,49	37,88	63,35
Upper 95% CI	904,6	347,3	452,8	141,5	198,6
Mean ranks	70,88	43,84	42,63	32,59	35,98

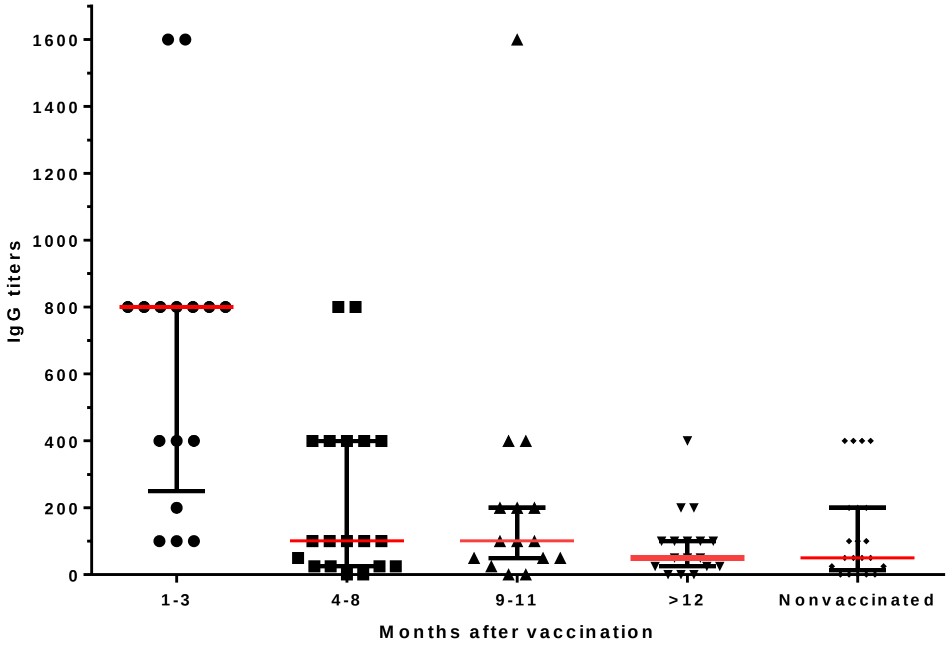

Supplement: S1 Dataset — (PDF) [file pone.0260202.s016.pdf]
