# Supplementary material for: Characterization of the adaptive immune response of donors receiving live anthrax vaccine
Source: PLoS One. 2021 Dec 20;16(12):e0260202. doi: 10.1371/journal.pone.0260202 (PMC8687594; doi:10.1371/journal.pone.0260202)

Level of specific IgG to full-length LF antigens of *B. anthracis* in the samples of blood serum from the donors.

The data are presented by a median titer with an interquartile range as a characteristic of the spread of values in the groups. The distribution was analysed using the Shapiro-Wilk test. The data were analysed using the Kruskal-Wallis test with multiple Dunn's comparisons in a One-Way ANOVA.

	**Months after Vaccination**				
	**1-3**	**4-8**	**9-11**	**>12**	**Nonvaccinated**
**Titers**	200	100	0	0	50
	800	100	400	0	25
	400	200	400	0	25
	100	25	200	25	200
	400	400	800	0	50
	200	1600	100	200	200
	800	400	200	0	0
	800	800	100	0	50
	400	400	0	0	100
	800	800	100	200	50
	800	25	0	50	25
	400	25	25	0	0
	100	25	25	25	0
	50	100	200	25	0
	400	100	100	50	0
	400	400		25	0
		25		0	0
		100			100
		25			200
					100
					0

**One-Way ANOVA**	
**Table Analyzed**	**LF full-length**
Kruskal-Wallis test	
P value	< 0,0001
Exact or approximate P value?	Approximate
P value summary	****
Do the medians vary signif. (P < 0.05)	Yes
Number of groups	5
Kruskal-Wallis statistic	36,17
Data summary	
Number of treatments (columns)	5
Number of values (total)	88

ANOVA Multiple Comparison						
Number of families	1					
Number of comparisons per family	4					
Alpha	0,05					
Dunn's multiple comparisons test	Mean rank diff,	Significant?	Summary			
1-3 vs. 4-8	16	No	ns			
1-3 vs. 9-11	23,23	Yes	*			
1-3 vs. >12	46	Yes	****			
1-3 vs. Nonvaccinated	38,67	Yes	****			
Test details	Mean rank 1	Mean rank 2	Mean rank diff,	n1	n2	
1-3 vs. 4-8	70,03	54,03	16	16	19	
1-3 vs. 9-11	70,03	46,8	23,23	16	15	
1-3 vs. >12	70,03	24,03	46	16	17	
1-3 vs. Nonvaccinated	70,03	31,36	38,67	16	21	

Descriptive Statistics					
	1-3	4-8	9-11	>12	Nonvaccinated
Number of values	16	19	15	17	21
Minimum	50	25	0	0	0
25% Percentile	200	25	25	0	0
Median	400	100	100	0	25
75% Percentile	800	400	200	37,5	100
Maximum	800	1600	800	200	200
Mean	440,6	297,4	176,7	35,29	55,95
Std. Deviation	277,6	400,9	216	64,38	69,33
Std. Error of Mean	69,41	91,98	55,77	15,62	15,13
Lower 95% CI	292,7	104,1	57,06	2,191	24,39
Upper 95% CI	588,6	490,6	296,3	68,4	87,51
Mean ranks	70,03	54,03	46,8	24,03	31,36

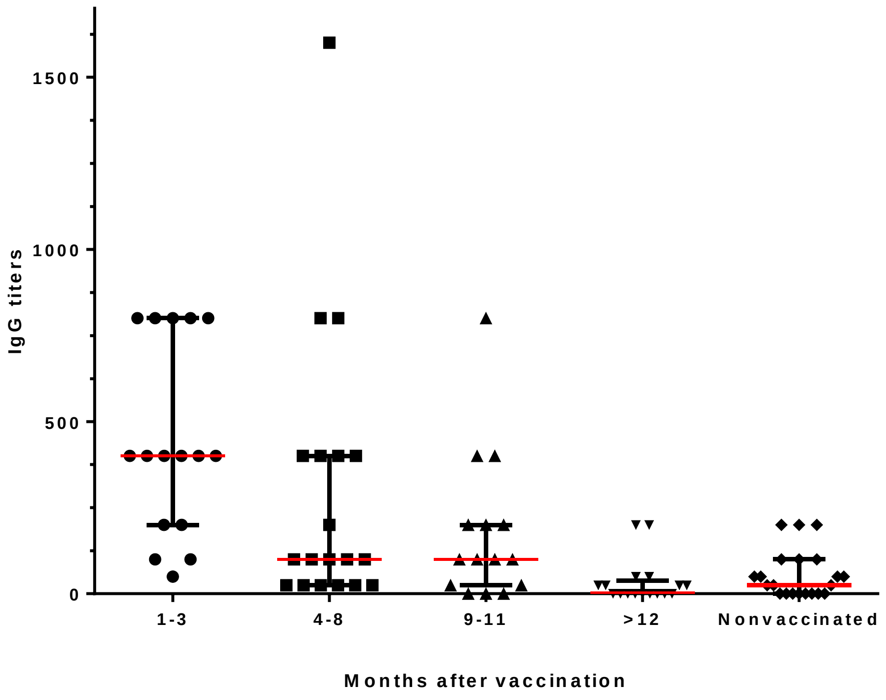

Supplement: S2 Dataset — (PDF) [file pone.0260202.s017.pdf]
