# Supplementary material for: Characterization of the adaptive immune response of donors receiving live anthrax vaccine
Source: PLoS One. 2021 Dec 20;16(12):e0260202. doi: 10.1371/journal.pone.0260202 (PMC8687594; doi:10.1371/journal.pone.0260202)

Level of specific IgG to spores antigens of *B. anthracis* in the samples of blood serum from the donors.

The data are presented by a median titer with an interquartile range as a characteristic of the spread of values in the groups. The distribution was analysed using the Shapiro-Wilk test. The data were analysed using the Kruskal-Wallis test with multiple Dunn’s comparisons in a One-Way ANOVA.

	**Months after Vaccination**				
	**1-3**	**4-8**	**9-11**	**>12**	**Nonvaccinated**
**Titers**	200	200	0	200	200
	1600	400	200	200	100
	200	400	100	100	0
	400	800	400	0	0
	1600	400	800	200	50
	800	1600	400	1600	200
	3200	800	200	200	50
	1600	1600	200	200	100
	3200	3200	0	400	800
	1600	200	25	800	400
	800	200	50	400	0
	800	100	100	200	50
	800	400	100	200	0
	400	200	200	0	50
	400	100	400	400	100
	1600	800		200	50
		50		0	25
		400			25
		200			0
					100
					0

**One-Way ANOVA**	
**Table Analyzed**	**Spores titers**
**Kruskal-Wallis test**	
P value	< 0,0001
Exact or approximate P value?	Approximate
P value summary	****
Do the medians vary signif. (P < 0.05)	Yes
Number of groups	5
Kruskal-Wallis statistic	38,63
Data summary	
Number of treatments (columns)	5
Number of values (total)	88

**ANOVA Multiple Comparison**					
**Number of families**	1				
**Number of comparisons per family**	10				
**Alpha**	0,05				
**Dunn's multiple comparisons test**	**Mean rank diff,**	**Significant?**	**Summary**		
**1-3 vs. 4-8**	15,78	No	ns		
**1-3 vs. 9-11**	34,77	Yes	**		
**1-3 vs. >12**	28,94	Yes	**		
**1-3 vs. Nonvaccinated**	48,37	Yes	****		
**4-8 vs. 9-11**	18,98	No	ns		
**4-8 vs. >12**	13,15	No	ns		
**4-8 vs. Nonvaccinated**	32,59	Yes	***		
**9-11 vs. >12**	-5,829	No	ns		
**9-11 vs. Nonvaccinated**	13,6	No	ns		
**>12 vs. Nonvaccinated**	19,43	No	ns		
**Test details**	**Mean rank 1**	**Mean rank 2**	**Mean rank diff,**	**n1**	**n2**
**1-3 vs. 4-8**	70,97	55,18	15,78	16	19
**1-3 vs. 9-11**	70,97	36,2	34,77	16	15
**1-3 vs. >12**	70,97	42,03	28,94	16	17
**1-3 vs. Nonvaccinated**	70,97	22,6	48,37	16	21
**4-8 vs. 9-11**	55,18	36,2	18,98	19	15
**4-8 vs. >12**	55,18	42,03	13,15	19	17
**4-8 vs. Nonvaccinated**	55,18	22,6	32,59	19	21
**9-11 vs. >12**	36,2	42,03	-5,829	15	17
**9-11 vs. Nonvaccinated**	36,2	22,6	13,6	15	21
**>12 vs. Nonvaccinated**	42,03	22,6	19,43	17	21

Descriptive Statistics					
	**1-3**	**4-8**	**9-11**	**>12**	**Nonvaccinated**
**Number of values**	16	19	15	17	21
**Minimum**	200	50	0	0	0
**25% Percentile**	400	200	50	150	0
**Median**	800	400	200	200	50
**75% Percentile**	1600	800	400	400	100
**Maximum**	3200	3200	800	1600	800
**Mean**	1200	634,2	211,7	311,8	109,5
**Std. Deviation**	943,8	769,6	214	383,9	184,6
**Std. Error of Mean**	235,9	176,6	55,25	93,1	40,29
**Lower 95% CI**	697,1	263,3	93,16	114,4	25,48
**Upper 95% CI**	1703	1005	330,2	509,1	193,6
**Mean ranks**	70,97	55,18	36,2	42,03	22,6

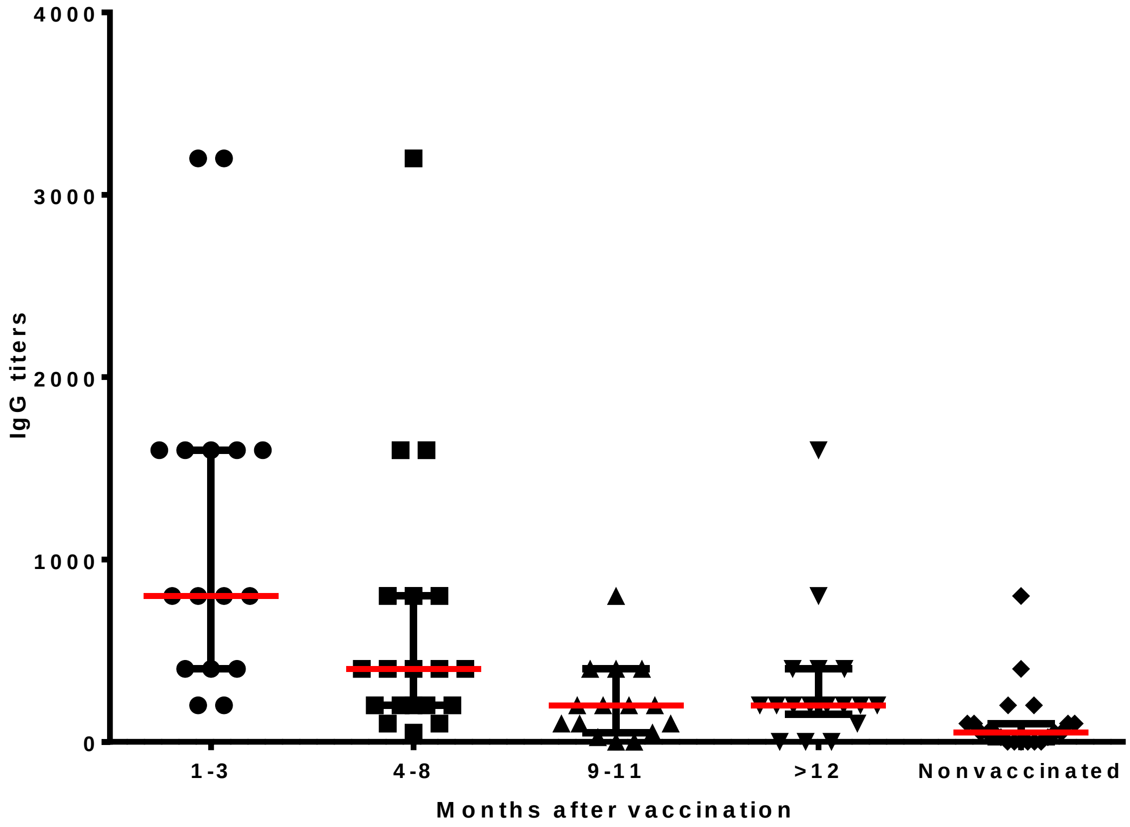

Supplement: S3 Dataset — (PDF) [file pone.0260202.s018.pdf]
