# Supplementary material for: Characterization of the adaptive immune response of donors receiving live anthrax vaccine
Source: PLoS One. 2021 Dec 20;16(12):e0260202. doi: 10.1371/journal.pone.0260202 (PMC8687594; doi:10.1371/journal.pone.0260202)

Level of specific IgG to PA-D1 of *B. anthracis* in the samples of blood serum from the donors.

The data are presented by a median titer with an interquartile range as a characteristic of the spread of values in the groups. The distribution was analysed using the Shapiro-Wilk test. The data were analysed using the Kruskal-Wallis test with multiple Dunn's comparisons in a One-Way ANOVA.

	**Months after Vaccination**				
	**1-3**	**4-8**	**9-11**	**>12**	**Nonvaccinated**
**Titers**	100	50	0	0	100
	400	50	100	100	25
	400	200	200	200	25
	200	100	200	50	0
	800	200	400	0	500
	200	400	400	100	100
	400	800	200	200	50
	1600	800	100	50	50
	400	1600	0	25	50
	800	200	200	100	0
	200	50	200	25	0
	400	25	200	0	0
	100	50	100	25	25
	200	100	400	50	25
	200	100	100	25	0
	400	200		100	0
		400		25	25
		50			25
		200			0
					0
					100

**One-Way ANOVA**	
**Table Analyzed**	**PA-D1 titers**
**Kruskal-Wallis test**	
P value	< 0,0001
Exact or approximate P value?	Approximate
P value summary	****
Do the medians vary signif. (P < 0.05)	Yes
Number of groups	5
Kruskal-Wallis statistic	40,49
Data summary	
Number of treatments (columns)	5
Number of values (total)	88

ANOVA Multiple Comparison					
**Number of families**	1				
**Number of comparisons per family**	10				
**Alpha**	0,05				
**Dunn's multiple comparisons test**	**Mean rank diff,**	**Significant?**	**Summary**		
**1-3 vs. 4-8**	15,42	No	ns		
**1-3 vs. 9-11**	16,71	No	ns		
**1-3 vs. >12**	39,17	Yes	****		
**1-3 vs. Nonvaccinated**	46,38	Yes	****		
**4-8 vs. 9-11**	1,295	No	ns		
**4-8 vs. >12**	23,75	Yes	*		
**4-8 vs. Nonvaccinated**	30,97	Yes	**		
**9-11 vs. >12**	22,45	No	ns		
**9-11 vs. Nonvaccinated**	29,67	Yes	**		
**>12 vs. Nonvaccinated**	7,218	No	ns		
**Test details**	**Mean rank 1**	**Mean rank 2**	**Mean rank diff,**	**n1**	**n2**
**1-3 vs. 4-8**	69,31	53,89	15,42	16	19
**1-3 vs. 9-11**	69,31	52,6	16,71	16	15
**1-3 vs. >12**	69,31	30,15	39,17	16	17
**1-3 vs. Nonvaccinated**	69,31	22,93	46,38	16	21
**4-8 vs. 9-11**	53,89	52,6	1,295	19	15
**4-8 vs. >12**	53,89	30,15	23,75	19	17
**4-8 vs. Nonvaccinated**	53,89	22,93	30,97	19	21
**9-11 vs. >12**	52,6	30,15	22,45	15	17
**9-11 vs. Nonvaccinated**	52,6	22,93	29,67	15	21
**>12 vs. Nonvaccinated**	30,15	22,93	7,218	17	21

Descriptive Statistics					
	**1-3**	**4-8**	**9-11**	**>12**	**Nonvaccinated**
**Number of values**	16	19	15	17	21
**Minimum**	100,0	25,00	0,0	0,0	0,0
**25% Percentile**	200,0	50,00	100,0	25,00	0,0
**Median**	400,0	200,0	200,0	50,00	25,00
**75% Percentile**	400,0	400,0	200,0	100,0	50,00
**Maximum**	1600	1600	400,0	200,0	500,0
**Mean**	425,0	293,4	186,7	63,24	52,38
**Std. Deviation**	376,8	392,1	130,2	62,57	108,1
**Std. Error of Mean**	94,21	89,95	33,62	15,18	23,59
**Lower 95% CI**	224,2	104,4	114,6	31,06	3,183
**Upper 95% CI**	625,8	482,4	258,8	95,41	101,6
**Mean ranks**	69,31	53,89	52,60	30,15	22,93

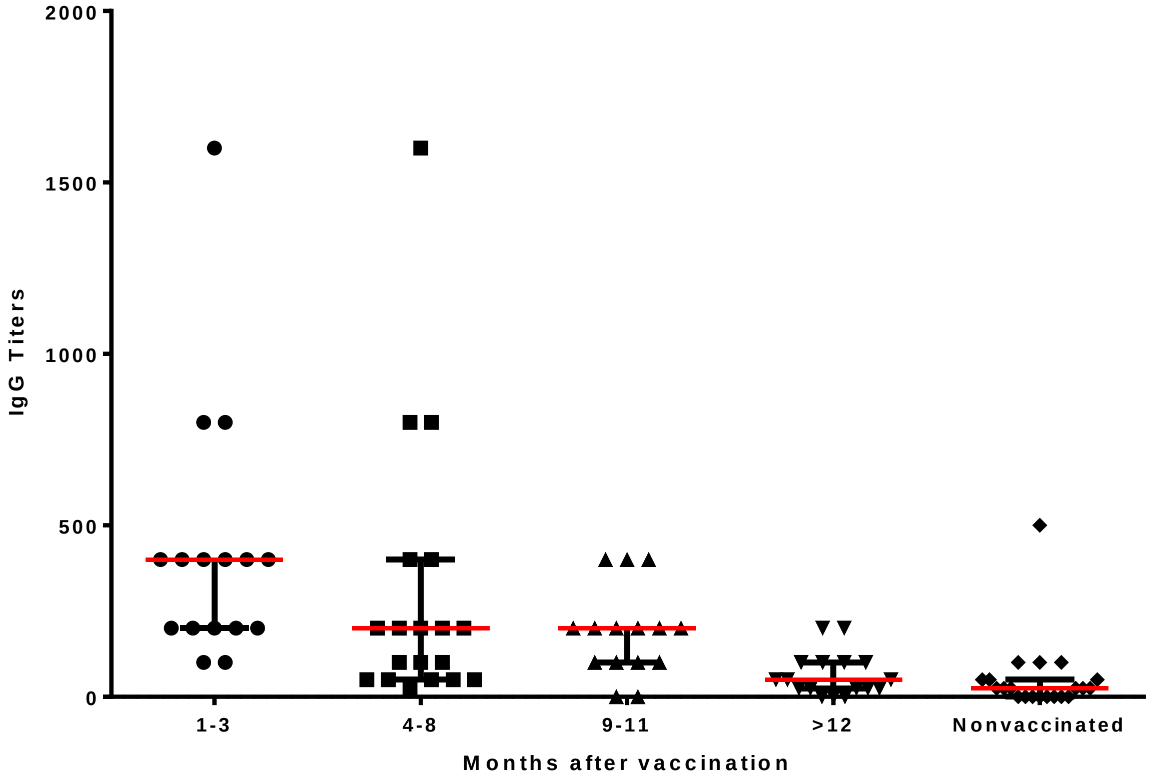

Supplement: S4 Dataset — (PDF) [file pone.0260202.s019.pdf]
