# Supplementary material for: Characterization of the adaptive immune response of donors receiving live anthrax vaccine
Source: PLoS One. 2021 Dec 20;16(12):e0260202. doi: 10.1371/journal.pone.0260202 (PMC8687594; doi:10.1371/journal.pone.0260202)

Level of specific IgG to PA-D2 of *B. anthracis* in the samples of blood serum from the donors.

The data are presented by a median titer with an interquartile range as a characteristic of the spread of values in the groups. The distribution was analysed using the Shapiro-Wilk test. The data were analysed using the Kruskal-Wallis test with multiple Dunn's comparisons in a One-Way ANOVA.

	**Months after Vaccination**				
	**1-3**	**4-8**	**9-11**	**>12**	**Nonvaccinated**
**Titers**	400	200	0	25	100
	800	100	100	200	25
	800	200	25	100	0
	800	400	25	200	0
	3200	0	1600	0	0
	800	50	100	100	100
	1600	1600	200	0	50
	400	400	200	50	50
	400	100	0	100	25
	800	200	50	200	0
	200	25	100	50	0
	200	100	200	200	0
	400	0	25	0	25
	200	100	800	25	50
	200	0	400	0	0
	400	25		50	0
		0		0	25
		0			25
		50			0
					0
					100

**One-Way ANOVA**	
**Table Analyzed**	**PA-D2 titers**
**Kruskal-Wallis test**	
P value	< 0,0001
Exact or approximate P value?	Approximate
P value summary	****
Do the medians vary signif. (P < 0.05)	Yes
Number of groups	5
Kruskal-Wallis statistic	38,66
Data summary	
Number of treatments (columns)	5
Number of values (total)	88

**ANOVA Multiple Comparison**					
**Number of families**	1				
**Number of comparisons per family**	10				
**Alpha**	0,05				
**Dunn's multiple comparisons test**	**Mean rank diff,**	**Significant?**	**Summary**		
**1-3 vs. 4-8**	32,45	Yes	**		
**1-3 vs. 9-11**	26,83	Yes	*		
**1-3 vs. >12**	38,12	Yes	***		
**1-3 vs. Nonvaccinated**	50,52	Yes	****		
**4-8 vs. 9-11**	-5,614	No	ns		
**4-8 vs. >12**	5,67	No	ns		
**4-8 vs. Nonvaccinated**	18,08	No	ns		
**9-11 vs. >12**	11,28	No	ns		
**9-11 vs. Nonvaccinated**	23,69	No	ns		
**>12 vs. Nonvaccinated**	12,41	No	ns		
**Test details**	**Mean rank 1**	**Mean rank 2**	**Mean rank diff,**	**n1**	**n2**
**1-3 vs. 4-8**	75,5	43,05	32,45	16	19
**1-3 vs. 9-11**	75,5	48,67	26,83	16	15
**1-3 vs. >12**	75,5	37,38	38,12	16	17
**1-3 vs. Nonvaccinated**	75,5	24,98	50,52	16	21
**4-8 vs. 9-11**	43,05	48,67	-5,614	19	15
**4-8 vs. >12**	43,05	37,38	5,67	19	17
**4-8 vs. Nonvaccinated**	43,05	24,98	18,08	19	21
**9-11 vs. >12**	48,67	37,38	11,28	15	17
**9-11 vs. Nonvaccinated**	48,67	24,98	23,69	15	21
**>12 vs. Nonvaccinated**	37,38	24,98	12,41	17	21

Descriptive Statistics					
	**1-3**	**4-8**	**9-11**	**>12**	**Nonvaccinated**
**Number of values**	16	19	15	17	21
**Minimum**	200	0	0	0	0
**25% Percentile**	250	0	25	0	0
**Median**	400	100	100	50	25
**75% Percentile**	800	200	200	150	50
**Maximum**	3200	1600	1600	200	100
**Mean**	725	186,8	255	76,47	27,38
**Std. Deviation**	755	363,8	425,9	78,79	35,27
**Std. Error of Mean**	188,7	83,47	110	19,11	7,697
**Lower 95% CI**	322,7	11,47	19,16	35,96	11,33
**Upper 95% CI**	1127	362,2	490,8	117	43,44
**Mean ranks**	75,5	43,05	48,67	37,38	24,98

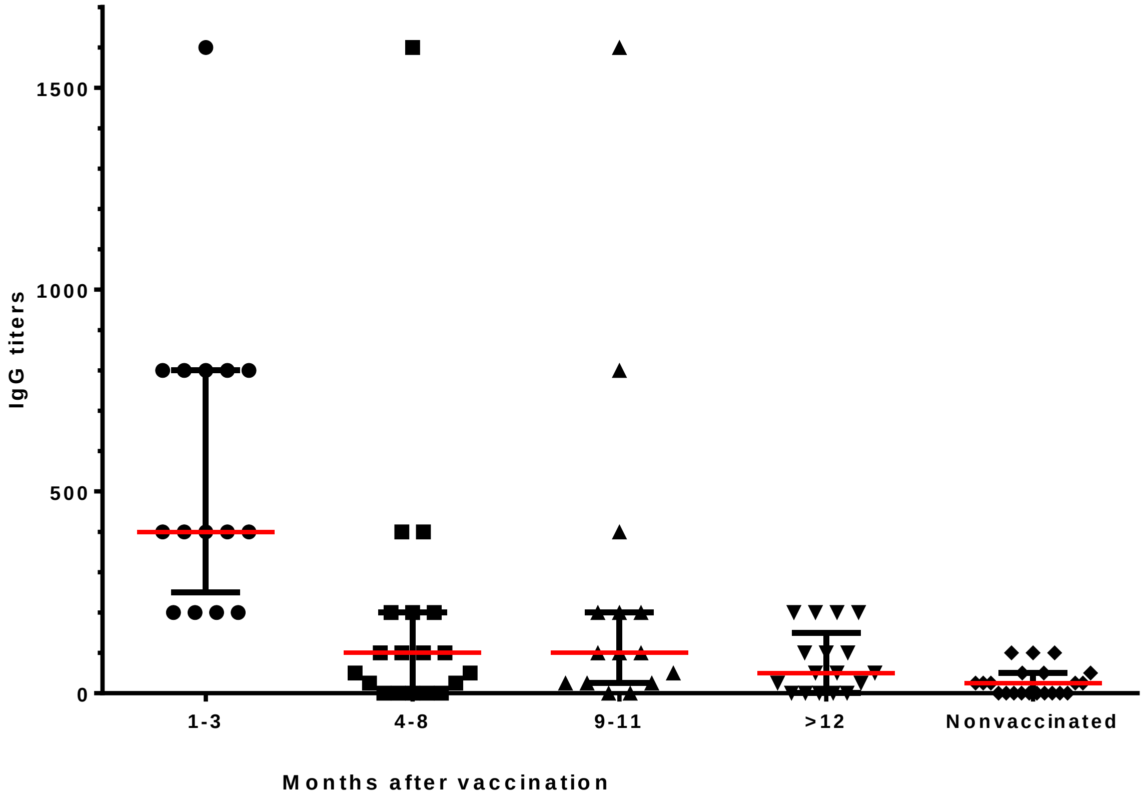

Supplement: S5 Dataset — (PDF) [file pone.0260202.s020.pdf]
