# Supplementary material for: Characterization of the adaptive immune response of donors receiving live anthrax vaccine
Source: PLoS One. 2021 Dec 20;16(12):e0260202. doi: 10.1371/journal.pone.0260202 (PMC8687594; doi:10.1371/journal.pone.0260202)

Level of specific IgG to PA-D3 of *B. anthracis* in the samples of blood serum from the donors.

The data are presented by a median titer with an interquartile range as a characteristic of the spread of values in the groups. The distribution was analysed using the Shapiro-Wilk test. The data were analysed using the Kruskal-Wallis test with multiple Dunn's comparisons in a One-Way ANOVA.

	**Months after Vaccination**				
	**1-3**	**4-8**	**9-11**	**>12**	**Nonvaccinated**
**Titers**	50	200	0	25	25
	400	200	800	50	0
	50	100	50	0	50
	800	100	25	25	100
	400	25	400	100	50
	200	50	50	400	100
	800	100	100	25	50
	50	200	100	0	0
	1600	800	0	0	25
	400	50	0	200	400
	200	50	100	200	0
	800	25	0	100	100
	3200	25	50	0	25
	50	25	400	0	50
	100	50	50	0	0
	100	100		0	400
		25		100	100
		100			25
		25			0
					25
					0

**One-Way ANOVA**	
**Table Analyzed**	**PA-D3 titers**
**Kruskal-Wallis test**	
P value	0,0005
Exact or approximate P value?	Approximate
P value summary	***
Do the medians vary signif. (P < 0.05)	Yes
Number of groups	5
Kruskal-Wallis statistic	19,97
Data summary	
Number of treatments (columns)	5
Number of values (total)	88

**ANOVA Multiple Comparison**					
**Number of families**	1				
**Number of comparisons per family**	10				
**Alpha**	0,05				
**Dunn's multiple comparisons test**	**Mean rank diff,**	**Significant?**	**Summary**		
**1-3 vs. 4-8**	20,21	No	ns		
**1-3 vs. 9-11**	24,94	No	ns		
**1-3 vs. >12**	33,91	Yes	**		
**1-3 vs. Nonvaccinated**	32,71	Yes	***		
**4-8 vs. 9-11**	4,73	No	ns		
**4-8 vs. >12**	13,7	No	ns		
**4-8 vs. Nonvaccinated**	12,5	No	ns		
**9-11 vs. >12**	8,975	No	ns		
**9-11 vs. Nonvaccinated**	7,771	No	ns		
**>12 vs. Nonvaccinated**	-1,203	No	ns		
**Test details**	**Mean rank 1**	**Mean rank 2**	**Mean rank diff,**	**n1**	**n2**
**1-3 vs. 4-8**	67,47	47,26	20,21	16	19
**1-3 vs. 9-11**	67,47	42,53	24,94	16	15
**1-3 vs. >12**	67,47	33,56	33,91	16	17
**1-3 vs. Nonvaccinated**	67,47	34,76	32,71	16	21
**4-8 vs. 9-11**	47,26	42,53	4,73	19	15
**4-8 vs. >12**	47,26	33,56	13,7	19	17
**4-8 vs. Nonvaccinated**	47,26	34,76	12,5	19	21
**9-11 vs. >12**	42,53	33,56	8,975	15	17
**9-11 vs. Nonvaccinated**	42,53	34,76	7,771	15	21
**>12 vs. Nonvaccinated**	33,56	34,76	-1,203	17	21

Descriptive Statistics					
	**1-3**	**4-8**	**9-11**	**>12**	**Nonvaccinated**
**Number of values**	16	19	15	17	21
**Minimum**	50	25	0	0	0
**25% Percentile**	62,5	25	0	0	0
**Median**	300	50	50	25	25
**75% Percentile**	800	100	100	100	100
**Maximum**	3200	800	800	400	400
**Mean**	575	118,4	141,7	72,06	72,62
**Std. Deviation**	817,3	176	223,5	107,8	114,5
**Std. Error of Mean**	204,3	40,37	57,72	26,16	24,99
**Lower 95% CI**	139,5	33,61	17,87	16,61	20,48
**Upper 95% CI**	1011	203,2	265,5	127,5	124,8
**Mean ranks**	67,47	47,26	42,53	33,56	34,76

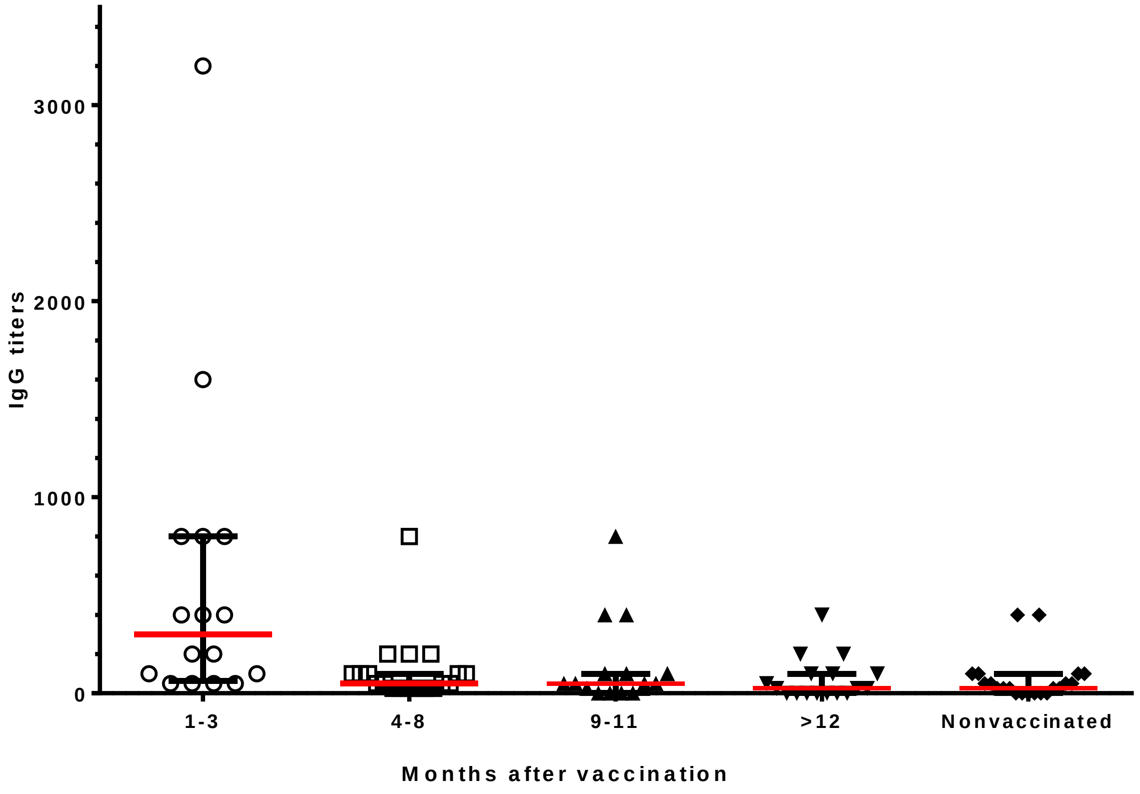

Supplement: S6 Dataset — (PDF) [file pone.0260202.s021.pdf]
