# Supplementary material for: Characterization of the adaptive immune response of donors receiving live anthrax vaccine
Source: PLoS One. 2021 Dec 20;16(12):e0260202. doi: 10.1371/journal.pone.0260202 (PMC8687594; doi:10.1371/journal.pone.0260202)

Level of specific IgG to PA-D4 of *B. anthracis* in the samples of blood serum from the donors.

The data are presented by a median titer with an interquartile range as a characteristic of the spread of values in the groups. The distribution was analysed using the Shapiro-Wilk test. The data were analysed using the Kruskal-Wallis test with multiple Dunn's comparisons in a One-Way ANOVA.

	**Months after Vaccination**				
	**1-3**	**4-8**	**9-11**	**>12**	**Nonvaccinated**
**Titers**	50	200	0	25	25
	400	800	400	400	0
	800	50	50	50	0
	200	50	50	50	100
	200	25	200	200	50
	200	200	100	100	0
	400	400	200	200	50
	1600	400	200	200	0
	200	400	0	0	0
	400	200	100	100	200
	100	100	400	400	50
	200	200	400	400	50
	400	100	200	0	0
	25	400	200	0	0
	400	100	100	0	25
	100	200		50	400
		25		100	0
		50			25
		25			0
					0
					25

**One-Way ANOVA**	
**Table Analyzed**	**PA-D4 titers**
**Kruskal-Wallis test**	
P value	< 0,0001
Exact or approximate P value?	Approximate
P value summary	****
Do the medians vary signif. (P < 0.05)	Yes
Number of groups	5
Kruskal-Wallis statistic	26,7
Data summary	
Number of treatments (columns)	5
Number of values (total)	88

**ANOVA Multiple Comparison**					
**Number of families**	1				
**Number of comparisons per family**	10				
**Alpha**	0,05				
**Dunn's multiple comparisons test**	**Mean rank diff,**	**Significant?**	**Summary**		
**1-3 vs. 4-8**	10,7	No	ns		
**1-3 vs. 9-11**	12,95	No	ns		
**1-3 vs. >12**	21,75	No	ns		
**1-3 vs. Nonvaccinated**	39,94	Yes	****		
**4-8 vs. 9-11**	2,253	No	ns		
**4-8 vs. >12**	11,05	No	ns		
**4-8 vs. Nonvaccinated**	29,24	Yes	**		
**9-11 vs. >12**	8,8	No	ns		
**9-11 vs. Nonvaccinated**	26,99	Yes	*		
**>12 vs. Nonvaccinated**	18,19	No	ns		
**Test details**	**Mean rank 1**	**Mean rank 2**	**Mean rank diff,**	**n1**	**n2**
**1-3 vs. 4-8**	62,75	52,05	10,7	16	19
**1-3 vs. 9-11**	62,75	49,8	12,95	16	15
**1-3 vs. >12**	62,75	41	21,75	16	17
**1-3 vs. Nonvaccinated**	62,75	22,81	39,94	16	21
**4-8 vs. 9-11**	52,05	49,8	2,253	19	15
**4-8 vs. >12**	52,05	41	11,05	19	17
**4-8 vs. Nonvaccinated**	52,05	22,81	29,24	19	21
**9-11 vs. >12**	49,8	41	8,8	15	17
**9-11 vs. Nonvaccinated**	49,8	22,81	26,99	15	21
**>12 vs. Nonvaccinated**	41	22,81	18,19	17	21

Descriptive Statistics					
	**1-3**	**4-8**	**9-11**	**>12**	**Nonvaccinated**
**Number of values**	16	19	15	17	21
**Minimum**	25	25	0	0	0
**25% Percentile**	125	50	50	12,5	0
**Median**	200	200	200	100	25
**75% Percentile**	400	400	200	200	50
**Maximum**	1600	800	400	400	400
**Mean**	354,7	206,6	173,3	133,8	47,62
**Std. Deviation**	383,9	198,4	137,4	144,4	93,51
**Std. Error of Mean**	95,97	45,52	35,48	35,03	20,41
**Lower 95% CI**	150,1	111	97,24	59,57	5,054
**Upper 95% CI**	559,2	302,2	249,4	208,1	90,18
**Mean ranks**	62,75	52,05	49,8	41	22,81

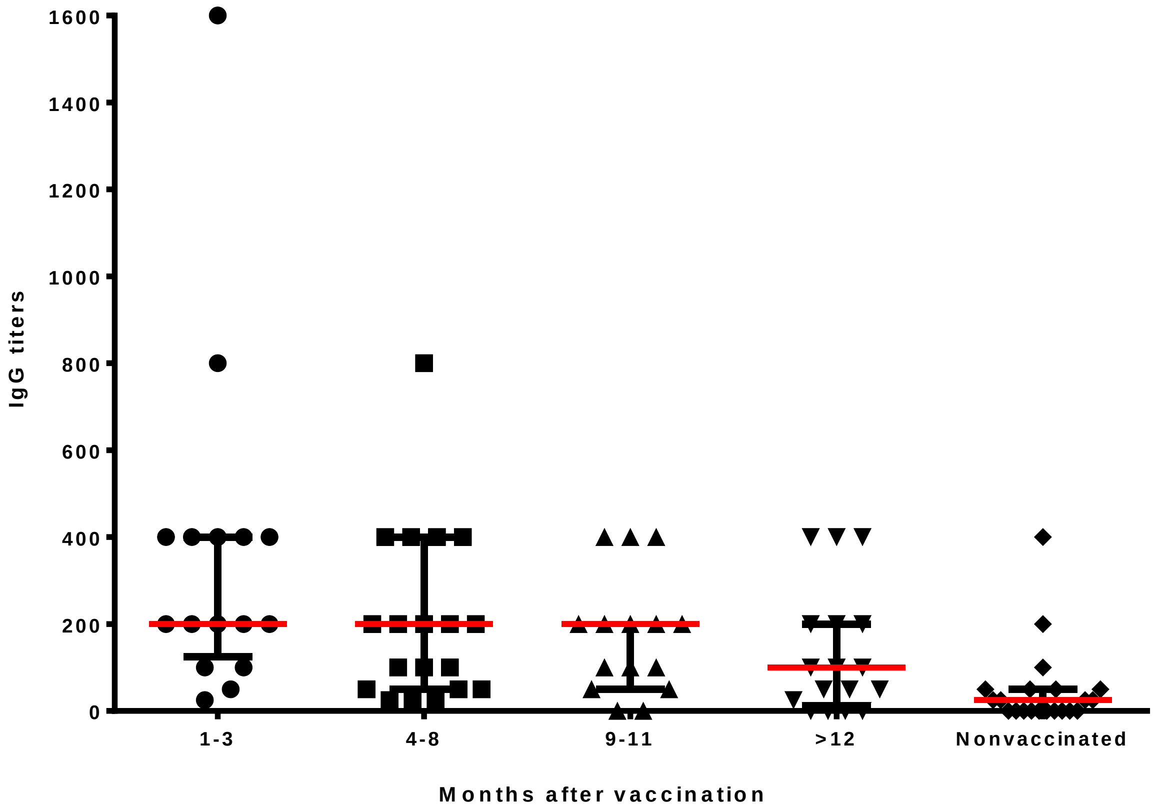

Supplement: S7 Dataset — (PDF) [file pone.0260202.s022.pdf]
