# Supplementary material for: Characterization of the adaptive immune response of donors receiving live anthrax vaccine
Source: PLoS One. 2021 Dec 20;16(12):e0260202. doi: 10.1371/journal.pone.0260202 (PMC8687594; doi:10.1371/journal.pone.0260202)

Level of specific IgG to LF-D1 of *B. anthracis* in the samples of blood serum from the donors.

The data are presented by a median titer with an interquartile range as a characteristic of the spread of values in the groups. The distribution was analysed using the Shapiro-Wilk test. The data were analysed using the Kruskal-Wallis test with multiple Dunn's comparisons in a One-Way ANOVA.

	**Months after Vaccination**				
	**1-3**	**4-8**	**9-11**	**>12**	**Nonvaccinated**
**Titers**	200	100	0	0	0
	200	50	400	0	0
	1600	400	1600	0	0
	400	0	1600	25	25
	1600	25	800	0	0
	3200	200	400	0	0
	1600	400	400	0	0
	50	400	800	0	0
	800	100	0	50	50
	200	400	200	100	100
	3200	0	0	100	100
	800	200	200	50	50
	400	100	50	50	50
	1600	200	200	100	100
	400	200	100	50	50
	200	800		200	200
		50		0	0
		400			0
		25			50
					0
					0

**One-Way ANOVA**	
**Table Analyzed**	**LF-D1 titers**
**Kruskal-Wallis test**	
P value	< 0,0001
Exact or approximate P value?	Approximate
P value summary	****
Do the medians vary signif. (P < 0.05)	Yes
Number of groups	5
Kruskal-Wallis statistic	41,66
Data summary	
Number of treatments (columns)	5
Number of values (total)	88

**ANOVA Multiple Comparison**					
**Number of families**	1				
**Number of comparisons per family**	10				
**Alpha**	0,05				
**Dunn's multiple comparisons test**	**Mean rank diff,**	**Significant?**	**Summary**		
**1-3 vs. 4-8**	21,51	No	ns		
**1-3 vs. 9-11**	16,31	No	ns		
**1-3 vs. >12**	44,08	Yes	****		
**1-3 vs. Nonvaccinated**	45,83	Yes	****		
**4-8 vs. 9-11**	-5,198	No	ns		
**4-8 vs. >12**	22,57	No	ns		
**4-8 vs. Nonvaccinated**	24,32	Yes	*		
**9-11 vs. >12**	27,77	Yes	*		
**9-11 vs. Nonvaccinated**	29,52	Yes	**		
**>12 vs. Nonvaccinated**	1,746	No	ns		
**Test details**	**Mean rank 1**	**Mean rank 2**	**Mean rank diff,**	**n1**	**n2**
**1-3 vs. 4-8**	71,38	49,87	21,51	16	19
**1-3 vs. 9-11**	71,38	55,07	16,31	16	15
**1-3 vs. >12**	71,38	27,29	44,08	16	17
**1-3 vs. Nonvaccinated**	71,38	25,55	45,83	16	21
**4-8 vs. 9-11**	49,87	55,07	-5,198	19	15
**4-8 vs. >12**	49,87	27,29	22,57	19	17
**4-8 vs. Nonvaccinated**	49,87	25,55	24,32	19	21
**9-11 vs. >12**	55,07	27,29	27,77	15	17
**9-11 vs. Nonvaccinated**	55,07	25,55	29,52	15	21
**>12 vs. Nonvaccinated**	27,29	25,55	1,746	17	21

Descriptive Statistics					
	**1-3**	**4-8**	**9-11**	**>12**	**Nonvaccinated**
**Number of values**	16	19	15	17	21
**Minimum**	50	0	0	0	0
**25% Percentile**	200	50	50	0	0
**Median**	600	200	200	25	0
**75% Percentile**	1600	400	800	75	50
**Maximum**	3200	800	1600	200	200
**Mean**	1028	213,2	450	42,65	36,9
**Std. Deviation**	1024	207,2	532,8	55,74	52,21
**Std. Error of Mean**	256	47,54	137,6	13,52	11,39
**Lower 95% CI**	482,5	113,3	154,9	13,99	13,14
**Upper 95% CI**	1574	313	745,1	71,3	60,67
**Mean ranks**	71,38	49,87	55,07	27,29	25,55

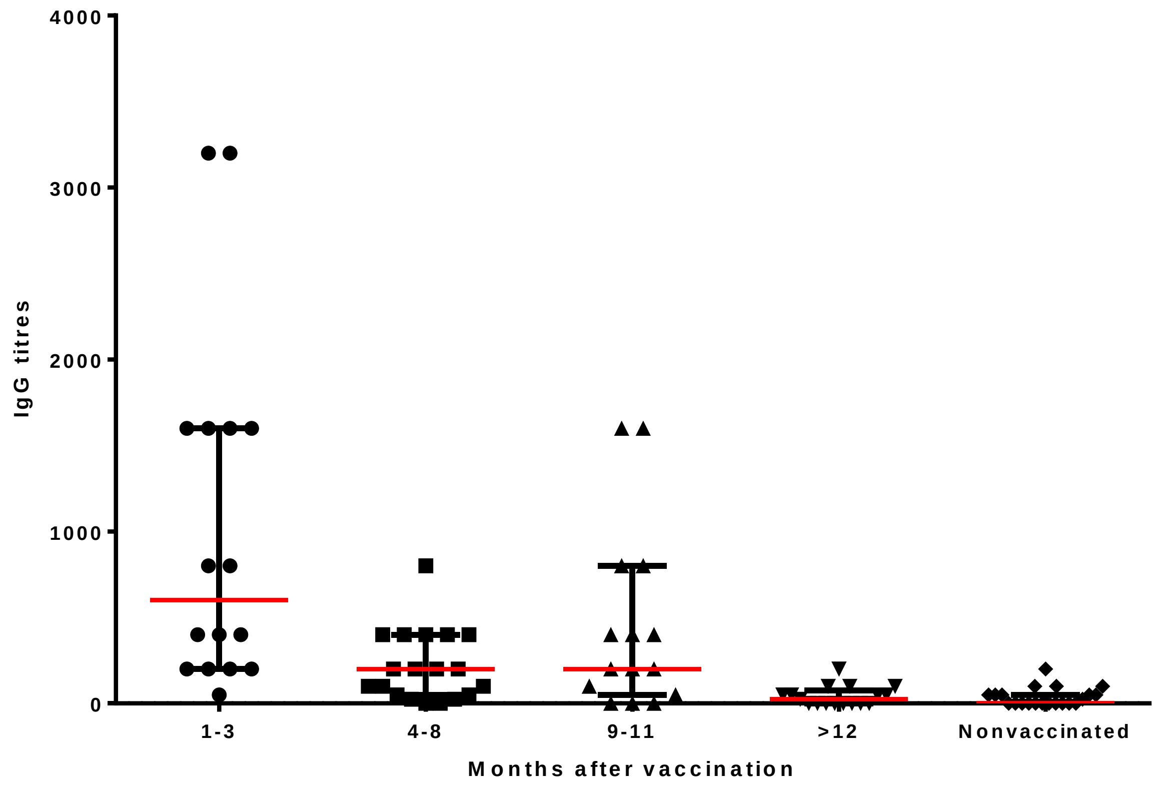

Supplement: S8 Dataset — (PDF) [file pone.0260202.s023.pdf]
