# Supplementary material for: Characterization of the adaptive immune response of donors receiving live anthrax vaccine
Source: PLoS One. 2021 Dec 20;16(12):e0260202. doi: 10.1371/journal.pone.0260202 (PMC8687594; doi:10.1371/journal.pone.0260202)

Level of specific IgG to LF-D2.3 of *B. anthracis* in the samples of blood serum from the donors.

The data are presented by a median titer with an interquartile range as a characteristic of the spread of values in the groups. The distribution was analysed using the Shapiro-Wilk test. The data were analysed using the Kruskal-Wallis test with multiple Dunn’s comparisons in a One-Way ANOVA.

	**Months after Vaccination**				
	**1-3**	**4-8**	**9-11**	**>12**	**Nonvaccinated**
	200	400	0	0	50
	400	200	400	0	0
	100	50	400	0	0
	400	100	200	0	0
	800	200	400	0	0
	800	400	200	100	0
	800	100	400	0	0
	3200	800	50	0	0
	800	400	0	0	25
	200	400	100	400	0
**Titers**	800	50	0	0	0
	400	200	25	0	0
	200	1600	0	0	0
	100	200	800	0	0
	50	0	50	0	0
	100	50		0	0
		100		0	0
		25			0
		0			0
					0
					0

**One-Way ANOVA**	
**Table Analyzed**	**LF-D2.3 titers**
**Kruskal-Wallis test**	
P value	< 0,0001
Exact or approximate P value?	Approximate
P value summary	****
Do the medians vary signif. (P < 0.05)	Yes
Number of groups	5
Kruskal-Wallis statistic	52,39
Data summary	
Number of treatments (columns)	5
Number of values (total)	88

**ANOVA Multiple Comparison**					
**Number of families**	1				
**Number of comparisons per family**	10				
**Alpha**	0,05				
**Dunn's multiple comparisons test**	**Mean rank diff,**	**Significant?**	**Summary**		
**1-3 vs. 4-8**	12,11	No	ns		
**1-3 vs. 9-11**	18,49	No	ns		
**1-3 vs. >12**	44,57	Yes	****		
**1-3 vs. Nonvaccinated**	47,4	Yes	****		
**4-8 vs. 9-11**	6,379	No	ns		
**4-8 vs. >12**	32,46	Yes	***		
**4-8 vs. Nonvaccinated**	35,29	Yes	****		
**9-11 vs. >12**	26,08	Yes	*		
**9-11 vs. Nonvaccinated**	28,91	Yes	**		
**>12 vs. Nonvaccinated**	2,832	No	ns		
**Test details**	**Mean rank 1**	**Mean rank 2**	**Mean rank diff,**	**n1**	**n2**
**1-3 vs. 4-8**	70,19	58,08	12,11	16	19
**1-3 vs. 9-11**	70,19	51,7	18,49	16	15
**1-3 vs. >12**	70,19	25,62	44,57	16	17
**1-3 vs. Nonvaccinated**	70,19	22,79	47,4	16	21
**4-8 vs. 9-11**	58,08	51,7	6,379	19	15
**4-8 vs. >12**	58,08	25,62	32,46	19	17
**4-8 vs. Nonvaccinated**	58,08	22,79	35,29	19	21
**9-11 vs. >12**	51,7	25,62	26,08	15	17
**9-11 vs. Nonvaccinated**	51,7	22,79	28,91	15	21
**>12 vs. Nonvaccinated**	25,62	22,79	2,832	17	21

Descriptive Statistics					
	**1-3**	**4-8**	**9-11**	**>12**	**Nonvaccinated**
**Number of values**	16	19	15	17	21
**Minimum**	50	0	0	0	0
**25% Percentile**	125	50	0	0	0
**Median**	400	200	100	0	0
**75% Percentile**	800	400	400	0	0
**Maximum**	3200	1600	800	400	50
**Mean**	584,4	277,6	201,7	29,41	3,571
**Std. Deviation**	757,6	378,1	233,8	98,52	11,95
**Std. Error of Mean**	189,4	86,75	60,38	23,89	2,608
**Lower 95% CI**	180,7	95,38	72,17	-21,24	-1,869
**Upper 95% CI**	988,1	459,9	331,2	80,07	9,012
**Mean ranks**	70,19	58,08	51,7	25,62	22,79

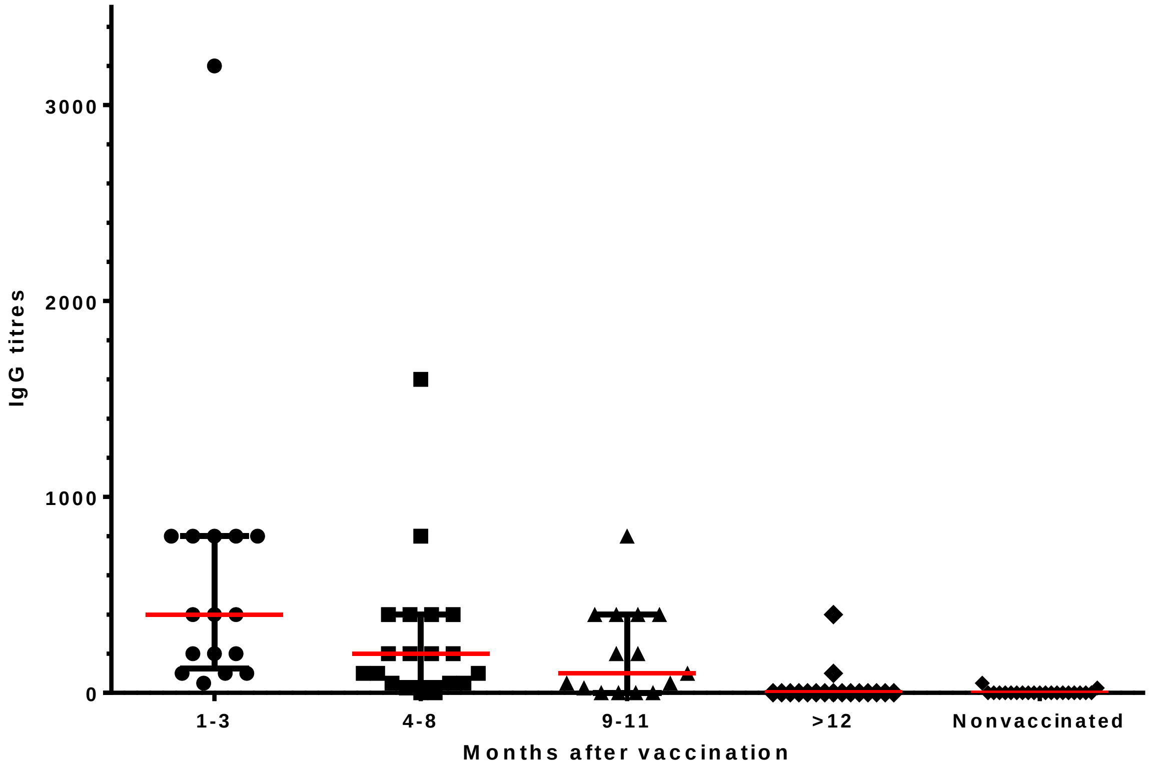

Supplement: S9 Dataset — (PDF) [file pone.0260202.s024.pdf]
