# Supplementary material for: Characterization of the adaptive immune response of donors receiving live anthrax vaccine
Source: PLoS One. 2021 Dec 20;16(12):e0260202. doi: 10.1371/journal.pone.0260202 (PMC8687594; doi:10.1371/journal.pone.0260202)

Level of specific IgG to LF-D4 of *B. anthracis* in the samples of blood serum from the donors.

The data are presented by a median titer with an interquartile range as a characteristic of the spread of values in the groups. The distribution was analysed using the Shapiro-Wilk test. The data were analysed using the Kruskal-Wallis test with multiple Dunn's comparisons in a One-Way ANOVA.

	**Months after Vaccination**				
	**1-3**	**4-8**	**9-11**	**>12**	**Nonvaccinated**
**Titers**	400	400	0	0	0
	800	25	400	0	0
	1600	50	800	50	0
	800	25	200	0	0
	1600	200	400	0	25
	3200	400	800	50	0
	1600	200	400	0	100
	3200	400	400	0	50
	800	400	0	0	0
	400	200	100	400	25
	800	50	0	0	0
	400	200	50	0	0
	200	800	100	0	0
	100	25	800	0	0
	800	200	200	25	0
	200	400		25	0
		200		0	0
		0			0
		25			50
					0
					0

**One-Way ANOVA**	
**Table Analyzed**	**LF-D4 titers**
**Kruskal-Wallis test**	
P value	< 0,0001
Exact or approximate P value?	Approximate
P value summary	****
Do the medians vary signif. (P < 0.05)	Yes
Number of groups	5
Kruskal-Wallis statistic	54,6
Data summary	
Number of treatments (columns)	5
Number of values (total)	88

**ANOVA Multiple Comparison**					
**Number of families**	1				
**Number of comparisons per family**	10				
**Alpha**	0,05				
**Dunn's multiple comparisons test**	**Mean rank diff,**	**Significant?**	**Summary**		
**1-3 vs. 4-8**	21,39	No	ns		
**1-3 vs. 9-11**	20,17	No	ns		
**1-3 vs. >12**	49,35	Yes	****		
**1-3 vs. Nonvaccinated**	51,87	Yes	****		
**4-8 vs. 9-11**	-1,221	No	ns		
**4-8 vs. >12**	27,96	Yes	**		
**4-8 vs. Nonvaccinated**	30,48	Yes	**		
**9-11 vs. >12**	29,18	Yes	**		
**9-11 vs. Nonvaccinated**	31,7	Yes	**		
**>12 vs. Nonvaccinated**	2,522	No	ns		
**Test details**	**Mean rank 1**	**Mean rank 2**	**Mean rank diff,**	**n1**	**n2**
**1-3 vs. 4-8**	74,47	53,08	21,39	16	19
**1-3 vs. 9-11**	74,47	54,3	20,17	16	15
**1-3 vs. >12**	74,47	25,12	49,35	16	17
**1-3 vs. Nonvaccinated**	74,47	22,6	51,87	16	21
**4-8 vs. 9-11**	53,08	54,3	-1,221	19	15
**4-8 vs. >12**	53,08	25,12	27,96	19	17
**4-8 vs. Nonvaccinated**	53,08	22,6	30,48	19	21
**9-11 vs. >12**	54,3	25,12	29,18	15	17
**9-11 vs. Nonvaccinated**	54,3	22,6	31,7	15	21
**>12 vs. Nonvaccinated**	25,12	22,6	2,522	17	21

Descriptive Statistics					
	**1-3**	**4-8**	**9-11**	**>12**	**Nonvaccinated**
**Number of values**	16	19	15	17	21
**Minimum**	100	0	0	0	0
**25% Percentile**	400	25	50	0	0
**Median**	800	200	200	0	0
**75% Percentile**	1600	400	400	25	12,5
**Maximum**	3200	800	800	400	100
**Mean**	1056	221,1	310	32,35	11,9
**Std. Deviation**	968,8	205,4	295,3	96,32	25,76
**Std. Error of Mean**	242,2	47,11	76,25	23,36	5,622
**Lower 95% CI**	540	122,1	146,5	-17,17	0,1779
**Upper 95% CI**	1572	320	473,5	81,88	23,63
**Mean ranks**	74,47	53,08	54,3	25,12	22,6

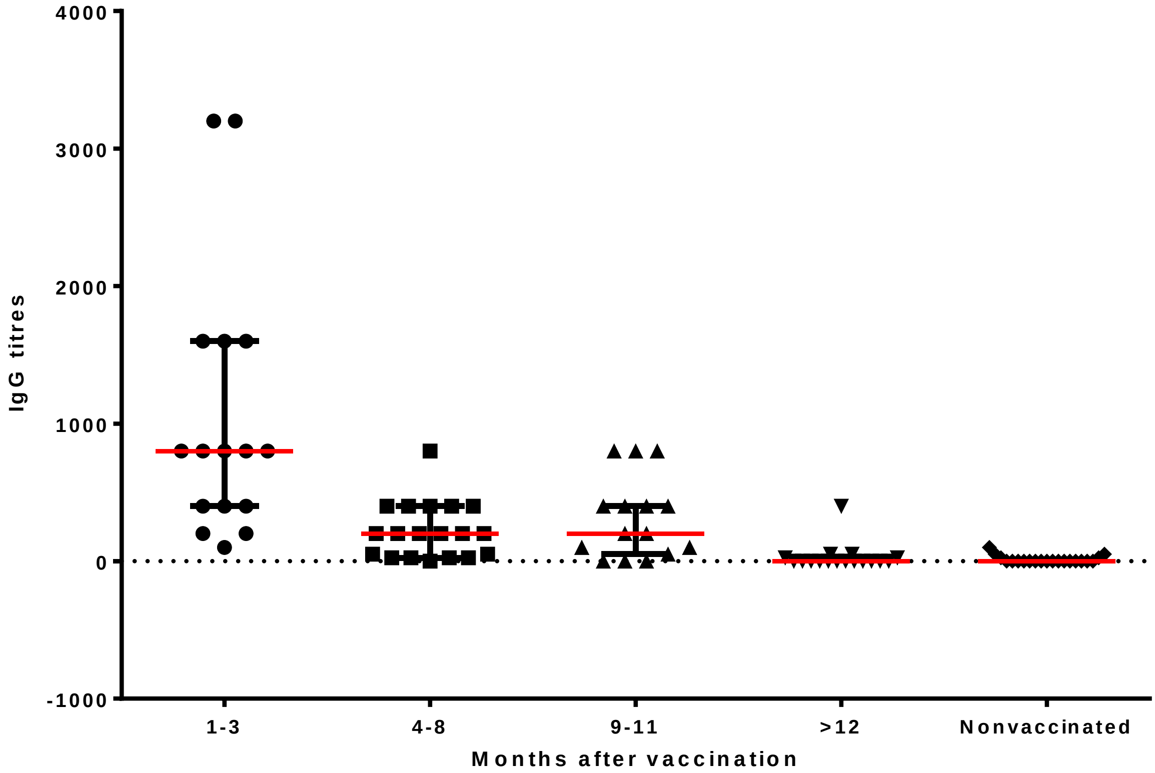

Supplement: S10 Dataset — (PDF) [file pone.0260202.s025.pdf]
