# Supplementary material for: Characterization of the adaptive immune response of donors receiving live anthrax vaccine
Source: PLoS One. 2021 Dec 20;16(12):e0260202. doi: 10.1371/journal.pone.0260202 (PMC8687594; doi:10.1371/journal.pone.0260202)

Toxin-neutralizing activity of the samples of blood serum (dilution 1:25) from the donors vaccinated against anthrax.

The data were analysed using the Kruskal-Wallis test with multiple Dunn comparisons in a One-Way ANOVA.

	Months after Vaccination				
	**1-3**	**4-8**	**9-11**	**>12**	**Nonvaccinated**
Viability, %	31,8346	20,84245	12,39132	27,67199	22,5884
	40,6576	26,864	38,8331	69,54984	13,64473
	39,43994	65,92554	40,8815	39,84637	7,89494
	48,82295	19,5403	26,1954	42,84774	11,95774
	59,92215	42,8114	78,75529	29,2283	16,8835
	50,8534	38,84354	43,8687	25,94855	12,5392
	51,34198	39,84127	36,83326	25,9164	9,47738
	43,81223	45,01134	16,9336	30,74883	13,88594
	53,91235	42,41965	14,038	11,83366	20,9758
	72,9756	29,92754	50,69034	57,8392	11,85338
	69,9372	23,3112	37,26688	25,564	6,9327
	30,19446	34,83672	40,61093	47,55232	9,833217
	27,84848	52,53543	29,16399	20,8193	13,74366
	22,82199	16,73286	41,73322	26,44944	15,6362
	43,9553	37,12018	56,61332	5,277291	6,8291
	37,28819	50,9942		29,93515	8,9672
		34,4423		13,53023	16,6483
		18,95376			11,81109
		33,38234			12,082
					10,6428
					8,88675

**One-Way ANOVA**	
**Table Analyzed**	**TNA for all donors**
**Kruskal-Wallis test**	
P value	< 0,0001
Exact or approximate P value?	Approximate
P value summary	****
Do the medians vary signif. (P < 0.05)	Yes
Number of groups	5
Kruskal-Wallis statistic	44,64
Data summary	
Number of treatments (columns)	5
Number of values (total)	88

**ANOVA Multiple Comparison**					
**Number of families**	1				
**Number of comparisons per family**	10				
**Alpha**	0,05				
**Dunn's multiple comparisons test**	**Mean rank diff,**	**Significant?**	**Summary**		
**1-3 vs. 4-8**	13,29	No	ns		
**1-3 vs. 9-11**	11,61	No	ns		
**1-3 vs. >12**	21,52	No	ns		
**1-3 vs. Nonvaccinated**	51,57	Yes	****		
**4-8 vs. 9-11**	-1,674	No	ns		
**4-8 vs. >12**	8,232	No	ns		
**4-8 vs. Nonvaccinated**	38,29	Yes	****		
**9-11 vs. >12**	9,906	No	ns		
**9-11 vs. Nonvaccinated**	39,96	Yes	****		
**>12 vs. Nonvaccinated**	30,06	Yes	**		
**Test details**	**Mean rank 1**	**Mean rank 2**	**Mean rank diff,**	**n1**	**n2**
**1-3 vs. 4-8**	65,81	52,53	13,29	16	19
**1-3 vs. 9-11**	65,81	54,2	11,61	16	15
**1-3 vs. >12**	65,81	44,29	21,52	16	17
**1-3 vs. Nonvaccinated**	65,81	14,24	51,57	16	21
**4-8 vs. 9-11**	52,53	54,2	-1,674	19	15
**4-8 vs. >12**	52,53	44,29	8,232	19	17
**4-8 vs. Nonvaccinated**	52,53	14,24	38,29	19	21
**9-11 vs. >12**	54,2	44,29	9,906	15	17
**9-11 vs. Nonvaccinated**	54,2	14,24	39,96	15	21
**>12 vs. Nonvaccinated**	44,29	14,24	30,06	17	21

Descriptive Statistics					
	**1-3**	**4-8**	**9-11**	**>12**	**Nonvaccinated**
**Number of values**	16	19	15	17	21
**Minimum**	22,82	16,73	12,39	5,277	6,829
**25% Percentile**	33,2	23,31	26,2	23,19	9,222
**Median**	43,88	34,84	38,83	27,67	11,96
**75% Percentile**	53,27	42,81	43,87	41,35	14,76
**Maximum**	72,98	65,93	78,76	69,55	22,59
**Mean**	45,35	35,49	37,65	31,21	12,56
**Std. Deviation**	14,34	12,99	17,16	16,3	4,216
**Std. Error of Mean**	3,585	2,98	4,431	3,954	0,9201
**Lower 95% CI**	37,71	29,23	28,15	22,83	10,64
**Upper 95% CI**	52,99	41,75	47,16	39,59	14,48
**Mean ranks**	65,81	52,53	54,2	44,29	14,24

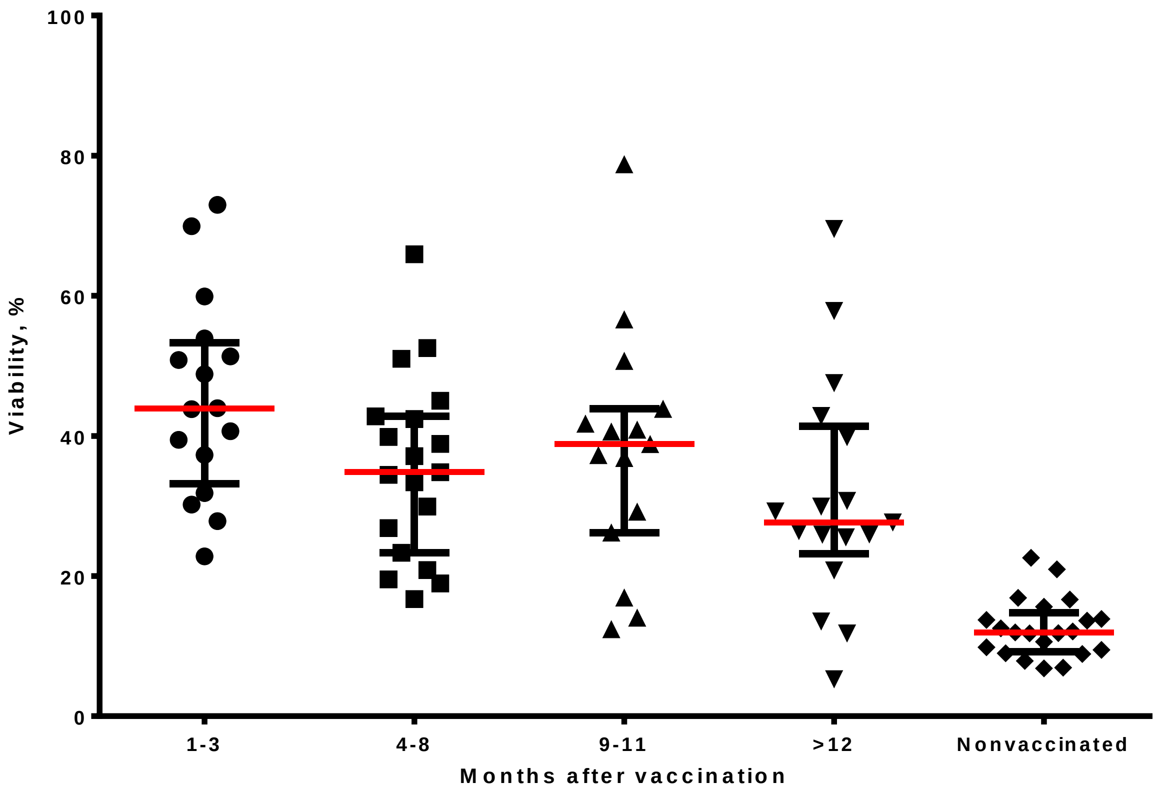

Supplement: S11 Dataset — (PDF) [file pone.0260202.s026.pdf]
