# Supplementary material for: Characterization of the adaptive immune response of donors receiving live anthrax vaccine
Source: PLoS One. 2021 Dec 20;16(12):e0260202. doi: 10.1371/journal.pone.0260202 (PMC8687594; doi:10.1371/journal.pone.0260202)

Correlation analysis between the toxin-neutralizing activity of the samples of blood serum from the donors and antibody titers against PA.

The data were analysed using the Spearman's rank correlation coefficient.

XY Data	
**TNA**	**Titers against PA**
31,834600	200
40,657600	800
39,439940	100
48,822950	800
59,922150	800
50,853400	100
51,341980	1600
43,812230	1600
53,912350	800
72,975600	800
69,937200	400
30,194460	100
27,848480	800
22,821990	800
43,955300	400
37,288190	400
20,842450	800
26,864000	400
65,925540	400
19,540300	100
42,811400	0
38,843540	25
39,841270	400
45,011340	800
42,419650	400
29,927540	25
23,311200	100
34,836720	25
52,535430	50
16,732860	100
37,120180	25
50,994200	400
34,442300	0
18,953760	100
33,382340	100

XY Data	
**TNA**	**Titers against PA**
12,391320	0
38,833100	400
40,881500	50
26,195400	100
78,755290	1600
43,868700	200
36,833260	200
16,933600	100
14,038000	0
50,690340	50
37,266880	50
40,610930	25
29,163990	100
41,733220	400
56,613320	200
27,671990	50
69,549840	25
39,846370	100
42,847740	100
29,228300	100
25,948550	200
25,916400	100
30,748830	50
11,833660	50
57,839200	400
25,564000	25
47,552320	200
20,819300	0
26,449440	0
5,277291	0
29,935150	25
13,530230	100

**Correlation.** **Tabular results**	
	**TNA vs. Titers against PA**
**Spearman r**	
**r**	0,4455
95% confidence interval	0,2230 to 0,6239
**P value**	
P (two-tailed)	0,0002
P value summary	***
Exact or approximate P value?	Approximate
Significant? (alpha = 0.05)	Yes
Number of XY Pairs	67

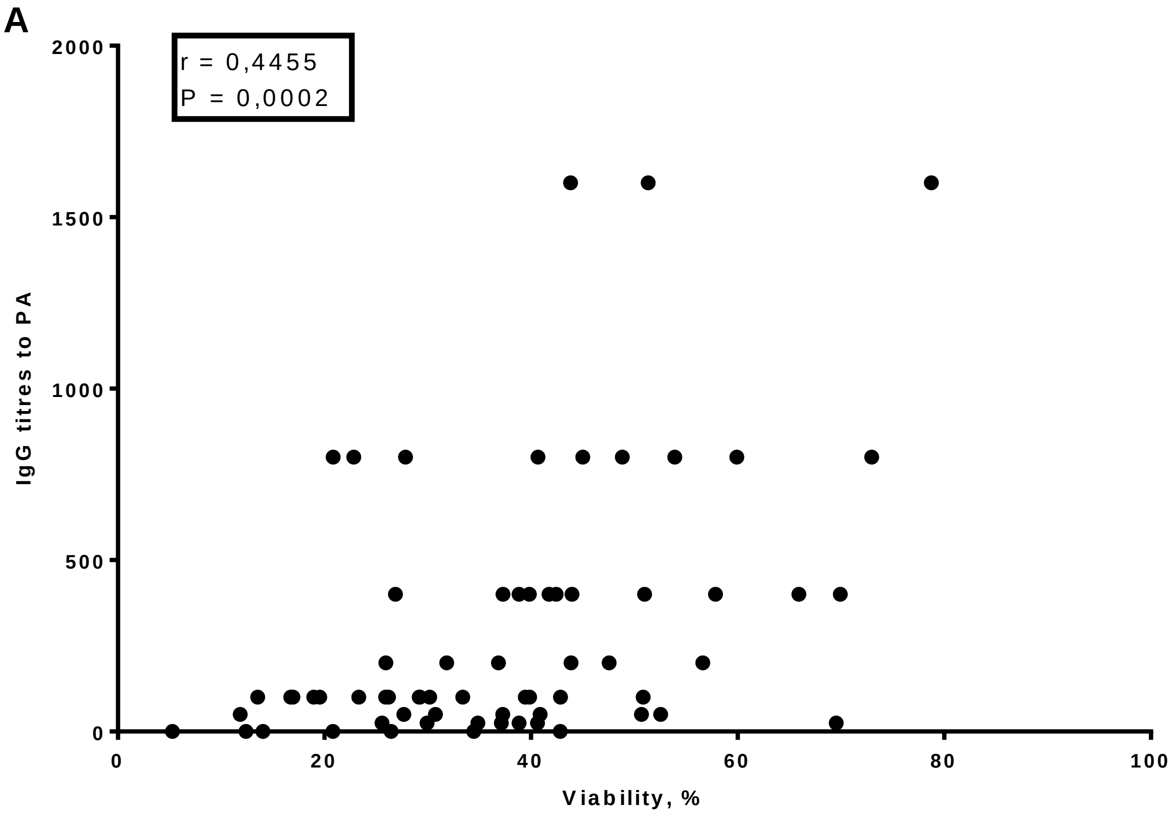

Supplement: S12 Dataset — (PDF) [file pone.0260202.s027.pdf]
