# Supplementary material for: Characterization of the adaptive immune response of donors receiving live anthrax vaccine
Source: PLoS One. 2021 Dec 20;16(12):e0260202. doi: 10.1371/journal.pone.0260202 (PMC8687594; doi:10.1371/journal.pone.0260202)

Correlation analysis between the toxin-neutralizing activity of the samples of blood serum from the donors and antibody titers against LF.

The data were analysed using the Spearman's rank correlation coefficient.

**XY Data**	
**TNA**	**Titers against LF**
31,8346	200
40,6576	800
39,43994	400
48,82295	100
59,92215	400
50,8534	200
51,34198	800
43,81223	800
53,91235	400
72,9756	800
69,9372	800
30,19446	400
27,84848	100
22,82199	50
43,9553	400
37,28819	400
20,84245	100
26,864	100
65,92554	200
19,5403	25
42,8114	400
38,84354	1600
39,84127	400
45,01134	800
42,41965	400
29,92754	800
23,3112	25
34,83672	25
52,53543	25
16,73286	100
37,12018	100
50,9942	400
34,4423	25
18,95376	100
33,38234	25

**XY Data**	
**TNA**	**Titers against LF**
12,39132	0
38,8331	400
40,8815	400
26,1954	200
78,75529	800
43,8687	100
36,83326	200
16,9336	100
14,038	0
50,69034	100
37,26688	0
40,61093	25
29,16399	25
41,73322	200
56,61332	100
27,67199	0
69,54984	0
39,84637	0
42,84774	25
29,2283	0
25,94855	200
25,9164	0
30,74883	0
11,83366	0
57,8392	200
25,564	50
47,55232	0
20,8193	25
26,44944	25
5,277291	50
29,93515	25
13,53023	0

**Correlation.** **Tabular results**	
	**TNA vs. Titers against LF**
**Spearman r**	
**r**	0,4902
95% confidence interval	0,2766 to 0,6576
**P value**	
P (two-tailed)	< 0,0001
P value summary	****
Exact or approximate P value?	Approximate
Significant? (alpha = 0.05)	Yes
Number of XY Pairs	67

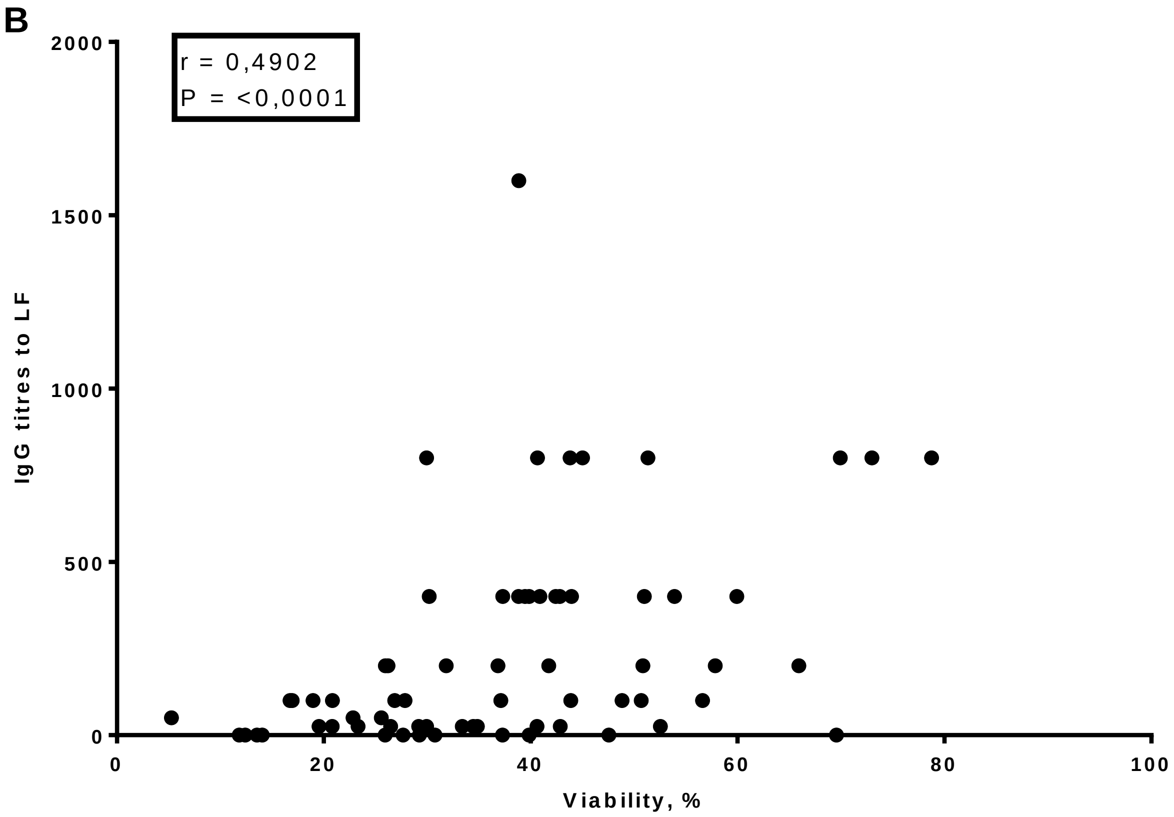

Supplement: S13 Dataset — (PDF) [file pone.0260202.s028.pdf]
