# Supplementary material for: Characterization of the adaptive immune response of donors receiving live anthrax vaccine
Source: PLoS One. 2021 Dec 20;16(12):e0260202. doi: 10.1371/journal.pone.0260202 (PMC8687594; doi:10.1371/journal.pone.0260202)

Correlation analysis between the toxin-neutralizing activity of the samples of blood serum from the donors and antibody titers against PA domains.

The data were analysed using the Spearman's rank correlation coefficient.

XY Data				
**TNA**	**Titers against PA-D1**	**Titers against PA-D2**	**Titers against PA-D3**	**Titers against PA-D4**
31,8346	100	400	50	50
40,6576	400	800	400	400
39,43994	400	800	50	800
48,82295	200	800	800	200
59,92215	800	3200	400	200
50,8534	200	800	200	200
51,34198	400	1600	800	400
43,81223	1600	400	50	1600
53,91235	400	400	1600	200
72,9756	800	800	400	400
69,9372	200	200	200	100
30,19446	400	200	800	200
27,84848	100	400	3200	400
22,82199	200	200	50	25
43,9553	200	200	100	400
37,28819	400	400	100	100
20,84245	50	200	200	200
26,864	50	100	200	800
65,92554	200	200	100	50
19,5403	100	400	100	50
42,8114	200	0	25	25
38,84354	400	50	50	200
39,84127	800	1600	100	400
45,01134	800	400	200	400
42,41965	1600	100	800	400
29,92754	200	200	50	200
23,3112	50	25	50	100
34,83672	25	100	25	200
52,53543	50	0	25	100
16,73286	100	100	25	400
37,12018	100	0	50	100
50,9942	200	25	100	200
34,4423	400	0	25	25
18,95376	50	0	100	50
33,38234	200	50	25	25
12,39132	0	0	0	0
38,8331	100	100	800	400
40,8815	200	25	50	50

XY Data				
**TNA**	**Titers against PA-D1**	**Titers against PA-D2**	**Titers against PA-D3**	**Titers against PA-D4**
26,1954	200	25	25	50
78,75529	400	1600	400	200
43,8687	400	100	50	100
36,83326	200	200	100	200
16,9336	100	200	100	200
14,038	0	0	0	0
50,69034	200	50	0	100
37,26688	200	100	100	400
40,61093	200	200	0	400
29,16399	100	25	50	200
41,73322	400	800	400	200
56,61332	100	400	50	100
27,67199	0	25	25	25
69,54984	100	200	50	400
39,84637	200	100	0	50
42,84774	50	200	25	50
29,2283	0	0	100	200
25,94855	100	100	400	100
25,9164	200	0	25	200
30,74883	50	50	0	200
11,83366	25	100	0	0
57,8392	100	200	200	100
25,564	25	50	200	400
47,55232	0	200	100	400
20,8193	25	0	0	0
26,44944	50	25	0	0
5,277291	25	0	0	0
29,93515	100	50	0	50
13,53023	25	0	100	100

**Correlation.** Tabular results				
	**TNA vs. Titers against PA-D1**	**TNA vs. Titers against PA-D2**	**TNA vs. Titers against PA-D3**	**TNA vs. Titers against PA-D4**
**Spearman r**				
**r**	0,5673	0,5156	0,3654	0,3494
95% confidence interval	0,3725 to 0,7143	0,3077 to 0,6765	0,1300 to 0,5617	0,1120 to 0,5491
**P value**				
P (two-tailed)	< 0,0001	< 0,0001	0,0024	0,0038
P value summary	****	****	**	**
Exact or approximate P value?	Approximate	Approximate	Approximate	Approximate
Significant? (alpha = 0.05)	Yes	Yes	Yes	Yes
Number of XY Pairs	67	67	67	67

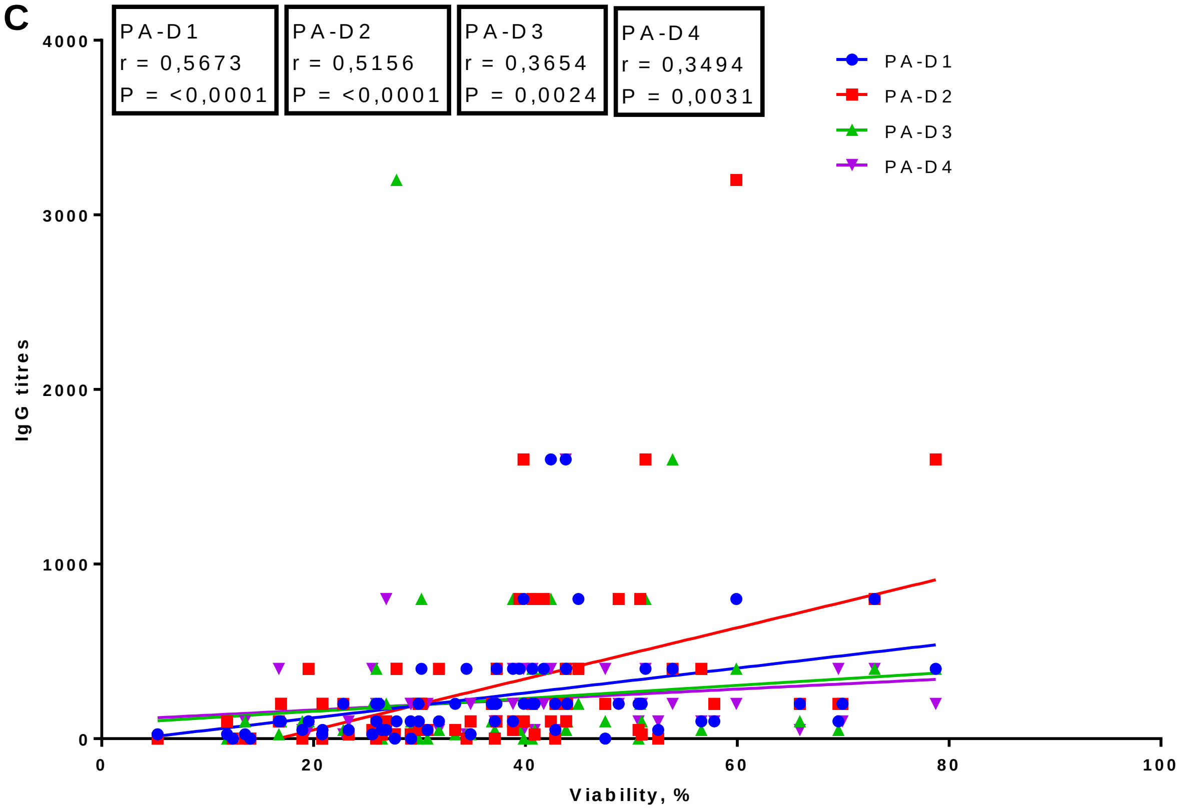

Supplement: S14 Dataset — (PDF) [file pone.0260202.s029.pdf]
