# Supplementary material for: Characterization of the adaptive immune response of donors receiving live anthrax vaccine
Source: PLoS One. 2021 Dec 20;16(12):e0260202. doi: 10.1371/journal.pone.0260202 (PMC8687594; doi:10.1371/journal.pone.0260202)

Correlation analysis between the toxin-neutralizing activity of the samples of blood serum from the donors and antibody titers against LF domains.

The data were analysed using the Spearman's rank correlation coefficient.

**XY Data**			
**TNA**	**Titers against LF-D1**	**Titers against LF-D2.3**	**Titers against LF-D4**
31,8346	200	200	400
40,6576	200	400	800
39,43994	1600	100	1600
48,82295	400	400	800
59,92215	1600	800	1600
50,8534	3200	800	3200
51,34198	1600	800	1600
43,81223	50	3200	3200
53,91235	800	800	800
72,9756	200	200	400
69,9372	3200	800	800
30,19446	800	400	400
27,84848	400	200	200
22,82199	1600	100	100
43,9553	400	50	800
37,28819	200	100	200
20,84245	100	400	400
26,864	50	200	25
65,92554	400	50	50
19,5403	0	100	25
42,8114	25	200	200
38,84354	200	400	400
39,84127	400	100	200
45,01134	400	800	400
42,41965	100	400	400
29,92754	400	400	200
23,3112	0	50	50
34,83672	200	200	200
52,53543	100	1600	800
16,73286	200	200	25
37,12018	200	0	200
50,9942	800	50	400
34,4423	50	100	200
18,95376	400	25	0
33,38234	25	0	25
12,39132	0	0	0
38,8331	400	400	400
40,8815	1600	400	800

XY Data			
**TNA**	**Titers against LF-D1**	**Titers against LF-D2.3**	**Titers against LF-D4**
78,75529	800	400	400
43,8687	400	200	800
36,83326	400	400	400
16,9336	800	50	400
14,038	0	0	0
50,69034	200	100	100
37,26688	0	0	0
40,61093	200	25	50
29,16399	50	0	100
41,73322	200	800	800
56,61332	100	50	200
27,67199	0	0	0
69,54984	0	0	0
39,84637	0	0	50
42,84774	25	0	0
29,2283	0	0	0
25,94855	0	100	50
25,9164	0	0	0
30,74883	0	0	0
11,83366	50	0	0
57,8392	100	400	400
25,564	100	0	0
47,55232	50	0	0
20,8193	50	0	0
26,44944	100	0	0
5,277291	50	0	25
29,93515	200	0	25
13,53023	0	0	0

**Correlation.** **Tabular results**			
	**TNA vs. Titers against LF-D1**	**TNA vs. Titers against LF-D2.3**	**TNA vs. Titers against LF-D4**
**Spearman r**			
**r**	0,3846	0,4796	0,5813
95% confidence interval	0,1519 to 0,5769	0,2638 to 0,6497	0,3903 to 0,7243
**P value**			
P (two-tailed)	0,0013	< 0,0001	< 0,0001
P value summary	**	****	****
Exact or approximate P value?	Approximate	Approximate	Approximate
Significant? (alpha = 0.05)	Yes	Yes	Yes
Number of XY Pairs	67	67	67

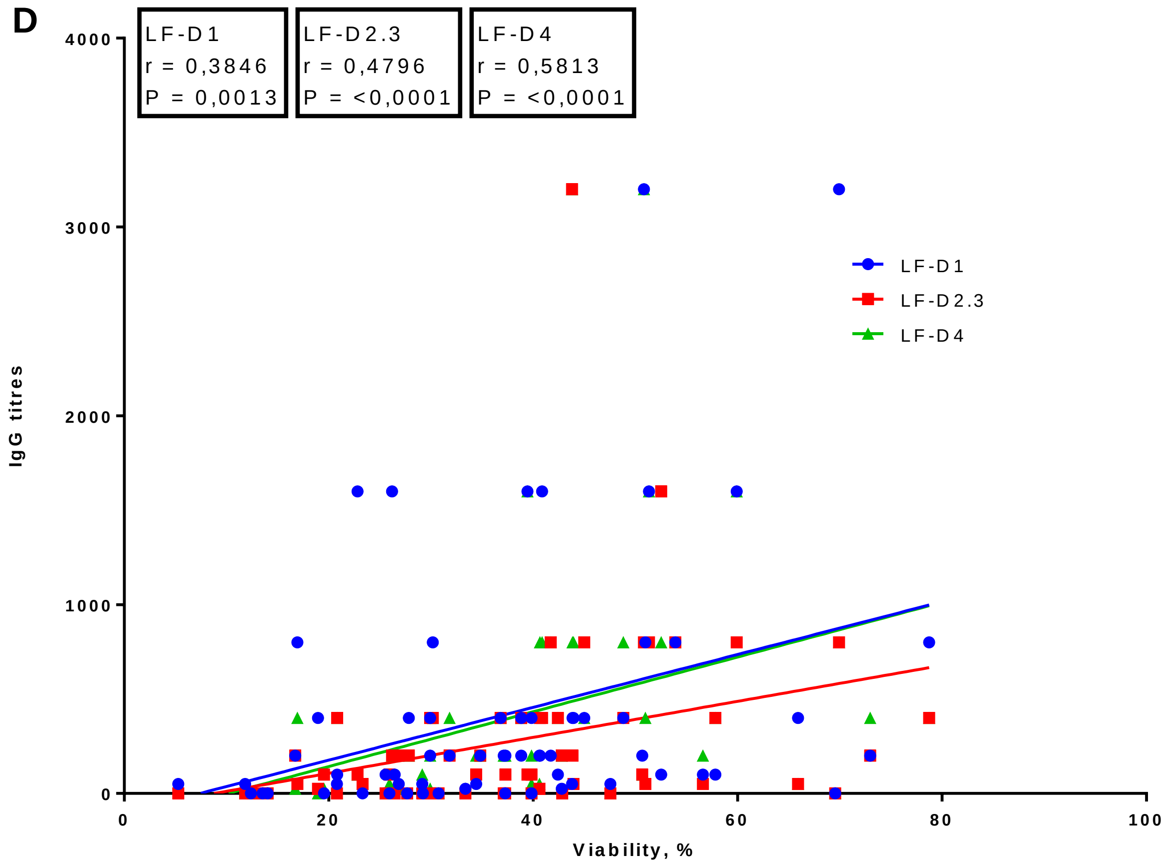

Supplement: S15 Dataset — (PDF) [file pone.0260202.s030.pdf]
