# Supplementary material for: Characterization of the adaptive immune response of donors receiving live anthrax vaccine
Source: PLoS One. 2021 Dec 20;16(12):e0260202. doi: 10.1371/journal.pone.0260202 (PMC8687594; doi:10.1371/journal.pone.0260202)

Analysis of the effect of age on the development and duration of anti-anthrax post-vaccination immunity (Age vs. PA titers).

Statistical analysis was performed using a Two-way ANOVA with Tukey's multiple comparison (determination of significance and confidence intervals). The histograms show the mean and the confidence interval (CI) as an interval estimate of the general frame.

	**Months after Vaccination**				
	**1-3**	**4-8**	**9-11**	**>12**	**Nonvaccinated**
**Titers in the group ages 20-40**	200	800	0	50	100
	100	0	400	50	400
	800	25	100	25	200
	100	400	1600	200	400
	1600	800	200	0	0
	800	25	200	0	50
	800	100	100	0	100
	100	25	0	25	25
	800	400	50		50
	400	0	100		0
		100	25		
		100			
**Titers in the group ages 40-60**	800	400	50	50	0
	800	400	50	25	0
	1600	100	400	100	50
	400	400	200	100	200
	800	25		100	400
	400	50		200	0
		100		100	400
				400	100
				100	50
					25
					200

**Two-Way ANOVA**						
**Table Analyzed**	**Age vs. PA titers**					
	**Ordinary**					
**Alpha**	0,05					
**Source of Variation**	**% of total variation**	**P value**	**P value summary**	**Significant?**		
**Interaction**	1,868	0,693	ns	No		
**Row Factor**	33,82	< 0,0001	****	Yes		
**Column Factor**	0,404	0,4888	ns	No		
**ANOVA table**	SS	DF	MS	F (DFn, DFd)	P value	
**Interaction**	200109	4	50027	F (4, 79) = 0,5591	P = 0,6930	
**Row Factor**	3623000	4	905650	F (4, 79) = 10,12	P < 0,0001	
**Column Factor**	43282	1	43282	F (1, 79) = 0,4838	P = 0,4888	
**Residual**	7068000	79	89472			
**Number of missing values**	31					

ANOVA Multiple Comparison

**Number of families**	1							
**Number of comparisons per family**	10							
**Alpha**	0,05							
**Tukey's multiple comparisons test**	**Mean Diff,**	**95% CI of diff,**	**Significant?**	**Summary**				
20-40 years								
**1-3 vs. 4-8**	338,8	-18,81 to 696,3	No	ns				
**1-3 vs. 9-12**	334,6	-22,97 to 692,1	No	ns				
**1-3 vs. >12**	526,3	130,1 to 922,4	Yes	**				
**1-3 vs. Nonvaccinated**	437,5	64,04 to 811,0	Yes	*				
**4-8 vs. 9-12**	-4,167	-345,1 to 336,8	No	ns				
**4-8 vs. >12**	187,5	-193,7 to 568,7	No	ns				
**4-8 vs. Nonvaccinated**	98,75	-258,8 to 456,3	No	ns				
**9-12 vs. >12**	191,7	-189,5 to 572,8	No	ns				
**9-12 vs. Nonvaccinated**	102,9	-254,6 to 460,5	No	ns				
**>12 vs. Nonvaccinated**	-88,75	-484,9 to 307,4	No	ns				
40-60 years								
**1-3 vs. 4-8**	589,3	124,7 to 1054	Yes	**				
**1-3 vs. 9-12**	625	85,96 to 1164	Yes	*				
**1-3 vs. >12**	669,4	229,3 to 1110	Yes	***				
**1-3 vs. Nonvaccinated**	670,5	246,6 to 1094	Yes	***				
**4-8 vs. 9-12**	35,71	-487,7 to 559,1	No	ns				
**4-8 vs. >12**	80,16	-340,7 to 501,0	No	ns				
**4-8 vs. Nonvaccinated**	81,17	-322,6 to 484,9	No	ns				
**9-12 vs. >12**	44,44	-457,4 to 546,3	No	ns				
**9-12 vs. Nonvaccinated**	45,45	-442,1 to 533,0	No	ns				
**>12 vs. Nonvaccinated**	1,01	-374,3 to 376,3	No	ns				
**Test details**	**Mean 1**	**Mean 2**	**Mean Diff,**	**SE of diff,**	**N1**	**N2**	**q**	**DF**
20-40 years								
**1-3 vs. 4-8**	570,0	231,3	338,8	128,1	10	12	3,741	79
**1-3 vs. 9-11**	570,0	235,4	334,6	128,1	10	12	3,694	79
**1-3 vs. >12**	570,0	43,75	526,3	141,9	10	8	5,245	79
**1-3 vs. Nonvaccinated**	570,0	132,5	437,5	133,8	10	10	4,625	79
**4-8 vs. 9-11**	231,3	235,4	-4,167	122,1	12	12	0,04825	79
**4-8 vs. >12**	231,3	43,75	187,5	136,5	12	8	1,942	79
**4-8 vs. Nonvaccinated**	231,3	132,5	98,75	128,1	12	10	1,090	79
**9-11 vs. >12**	235,4	43,75	191,7	136,5	12	8	1,985	79

**9-11 vs. Nonvaccinated**	235,4	132,5	102,9	128,1	12	10	1,136	79
**>12 vs. Nonvaccinated**	43,75	132,5	-88,75	141,9	8	10	0,8846	79
40-60 years								
**1-3 vs. 4-8**	800	210,7	589,3	166,4	6	7	5,008	79
**1-3 vs. 9-11**	800	175	625	193,1	6	4	4,578	79
**1-3 vs. >12**	800	130,6	669,4	157,6	6	9	6,005	79
**1-3 vs. Nonvaccinated**	800	129,5	670,5	151,8	6	11	6,246	79
**4-8 vs. 9-11**	210,7	175	35,71	187,5	7	4	0,2694	79
**4-8 vs. >12**	210,7	130,6	80,16	150,7	7	9	0,752	79
**4-8 vs. Nonvaccinated**	210,7	129,5	81,17	144,6	7	11	0,7937	79
**9-11 vs. >12**	175	130,6	44,44	179,7	4	9	0,3497	79
**9-11 vs. Nonvaccinated**	175	129,5	45,45	174,6	4	11	0,3681	79
**>12 vs. Nonvaccinated**	130,6	129,5	1,01	134,4	9	11	0,01063	79

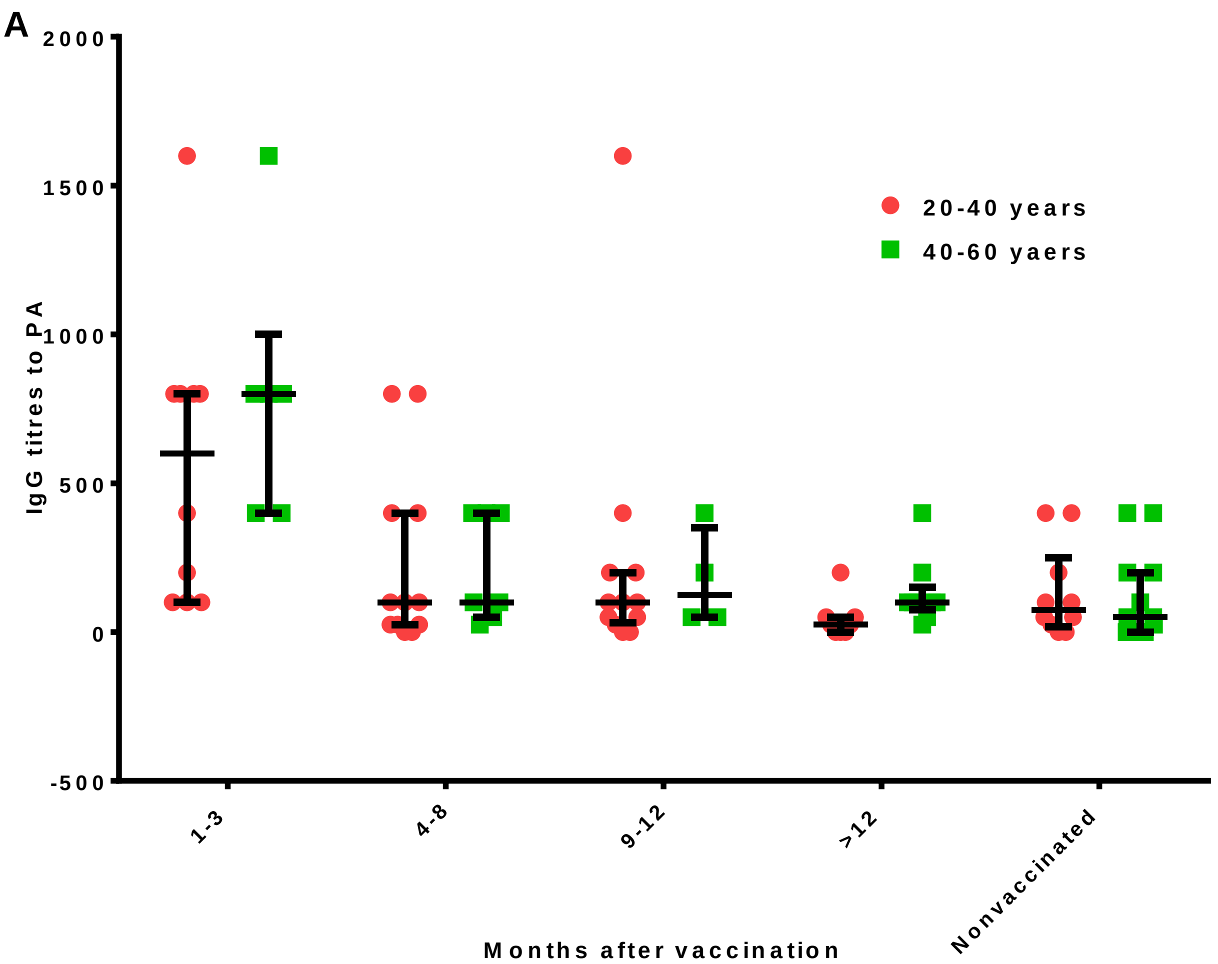

Supplement: S16 Dataset — (PDF) [file pone.0260202.s031.pdf]
