# Supplementary material for: Characterization of the adaptive immune response of donors receiving live anthrax vaccine
Source: PLoS One. 2021 Dec 20;16(12):e0260202. doi: 10.1371/journal.pone.0260202 (PMC8687594; doi:10.1371/journal.pone.0260202)

Analysis of the effect of age on the development and duration of anti-anthrax post-vaccination immunity (Age vs. LF titers).

Statistical analysis was performed using a Two-way ANOVA with Tukey's multiple comparison (determination of significance and confidence intervals). The histograms show the mean and the confidence interval (CI) as an interval estimate of the general frame.

	**Months after Vaccination**				
	**1-3**	**4-8**	**9-11**	**>12**	**Nonvaccinated**
**Titers in the group ages 20-40**	200	100	0	0	50
	400	400	400	0	200
	100	1600	200	50	50
	200	400	800	0	50
	800	800	100	25	100
	400	800	200	25	50
	800	25	100	50	0
	400	100	0	25	0
	50	400	100		0
	400	25	25		0
		100	25		
		25			
**Titers in the group ages 40-60**	800	100	400	0	25
	400	20	0	0	25
	800	25	200	0	200
	800	400	100	25	0
	100	25		0	25
	400	25		200	0
		100		0	0
				200	100
				0	200
					100
					0

**Two-Way ANOVA**						
**Table Analyzed**	**Age vs. LF titers**					
	**Ordinary**					
**Alpha**	0,05					
**Source of Variation**	**% of total variation**	**P value**	**P value summary**	**Significant?**		
**Interaction**	6,403	0,1116	ns	No		
**Row Factor**	28,99	< 0,0001	****	Yes		
**Column Factor**	0,04649	0,8129	ns	No		
**ANOVA table**	SS	DF	MS	F (DFn, DFd)	P value	
**Interaction**	440156	4	110039	F (4, 78) = 1,943	P = 0,1116	
**Row Factor**	1,993e+006	4	498245	F (4, 78) = 8,797	P < 0,0001	
**Column Factor**	3196	1	3196	F (1, 78) = 0,05643	P = 0,8129	
**Residual**	4,418e+006	78	56637			
**Number of missing values**	32					

ANOVA Multiple Comparison

**Number of families**	1							
**Number of comparisons per family**	10							
**Alpha**	0,05							
**Tukey's multiple comparisons test**	**Mean Diff,**	**95% CI of diff,**	**Significant?**	**Summary**				
20-40 years								
**1-3 vs. 4-8**	-22,92	-307,5 to 261,6	No	ns				
**1-3 vs. 9-12**	197,7	-92,66 to 488,1	No	ns				
**1-3 vs. >12**	353,1	37,88 to 668,4	Yes	*				
**1-3 vs. Nonvaccinated**	325	27,78 to 622,2	Yes	*				
**4-8 vs. 9-12**	220,6	-56,78 to 498,1	No	ns				
**4-8 vs. >12**	376	72,69 to 679,4	Yes	**				
**4-8 vs. Nonvaccinated**	347,9	63,35 to 632,5	Yes	**				
**9-12 vs. >12**	155,4	-153,4 to 464,2	No	ns				
**9-12 vs. Nonvaccinated**	127,3	-163,1 to 417,7	No	ns				
**>12 vs. Nonvaccinated**	-28,13	-343,4 to 287,1	No	ns				
40-60 years								
**1-3 vs. 4-8**	425	55,25 to 794,8	Yes	*				
**1-3 vs. 9-12**	375	-54,00 to 804,0	No	ns				
**1-3 vs. >12**	502,8	152,5 to 853,1	Yes	**				
**1-3 vs. Nonvaccinated**	488,6	151,3 to 825,9	Yes	**				
**4-8 vs. 9-12**	-50	-466,6 to 366,6	No	ns				
**4-8 vs. >12**	77,78	-257,2 to 412,7	No	ns				
**4-8 vs. Nonvaccinated**	63,64	-257,7 to 385,0	No	ns				
**9-12 vs. >12**	127,8	-271,6 to 527,2	No	ns				
**9-12 vs. Nonvaccinated**	113,6	-274,4 to 501,7	No	ns				
**>12 vs. Nonvaccinated**	-14,14	-312,9 to 284,6	No	ns				
**Test details**	**Mean 1**	**Mean 2**	**Mean Diff,**	**SE of diff,**	**N1**	**N2**	**q**	**DF**
20-40 years								
**1-3 vs. 4-8**	375	397,9	-22,92	101,9	10	12	0,318	78
**1-3 vs. 9-11**	375	177,3	197,7	104	10	11	2,689	78
**1-3 vs. >12**	375	21,88	353,1	112,9	10	8	4,424	78
**1-3 vs. Nonvaccinated**	375	50	325	106,4	10	10	4,318	78
**4-8 vs. 9-11**	397,9	177,3	220,6	99,34	12	11	3,141	78
**4-8 vs. >12**	397,9	21,88	376	108,6	12	8	4,896	78
**4-8 vs. Nonvaccinated**	397,9	50	347,9	101,9	12	10	4,829	78
**9-11 vs. >12**	177,3	21,88	155,4	110,6	11	8	1,987	78

**9-11 vs. Nonvaccinated**	177,3	50	127,3	104	11	10	1,731	78
**>12 vs. Nonvaccinated**	21,88	50	-28,13	112,9	8	10	0,3523	78
40-60 years								
**1-3 vs. 4-8**	550	125	425	132,4	6	7	4,539	78
**1-3 vs. 9-11**	550	175	375	153,6	6	4	3,452	78
**1-3 vs. >12**	550	47,22	502,8	125,4	6	9	5,669	78
**1-3 vs. Nonvaccinated**	550	61,36	488,6	120,8	6	11	5,721	78
**4-8 vs. 9-11**	125	175	-50	149,2	7	4	0,474	78
**4-8 vs. >12**	125	47,22	77,78	119,9	7	9	0,9171	78
**4-8 vs. Nonvaccinated**	125	61,36	63,64	115,1	7	11	0,7821	78
**9-11 vs. >12**	175	47,22	127,8	143	4	9	1,264	78
**9-11 vs. Nonvaccinated**	175	61,36	113,6	139	4	11	1,157	78
**>12 vs. Nonvaccinated**	47,22	61,36	-14,14	107	9	11	0,187	78

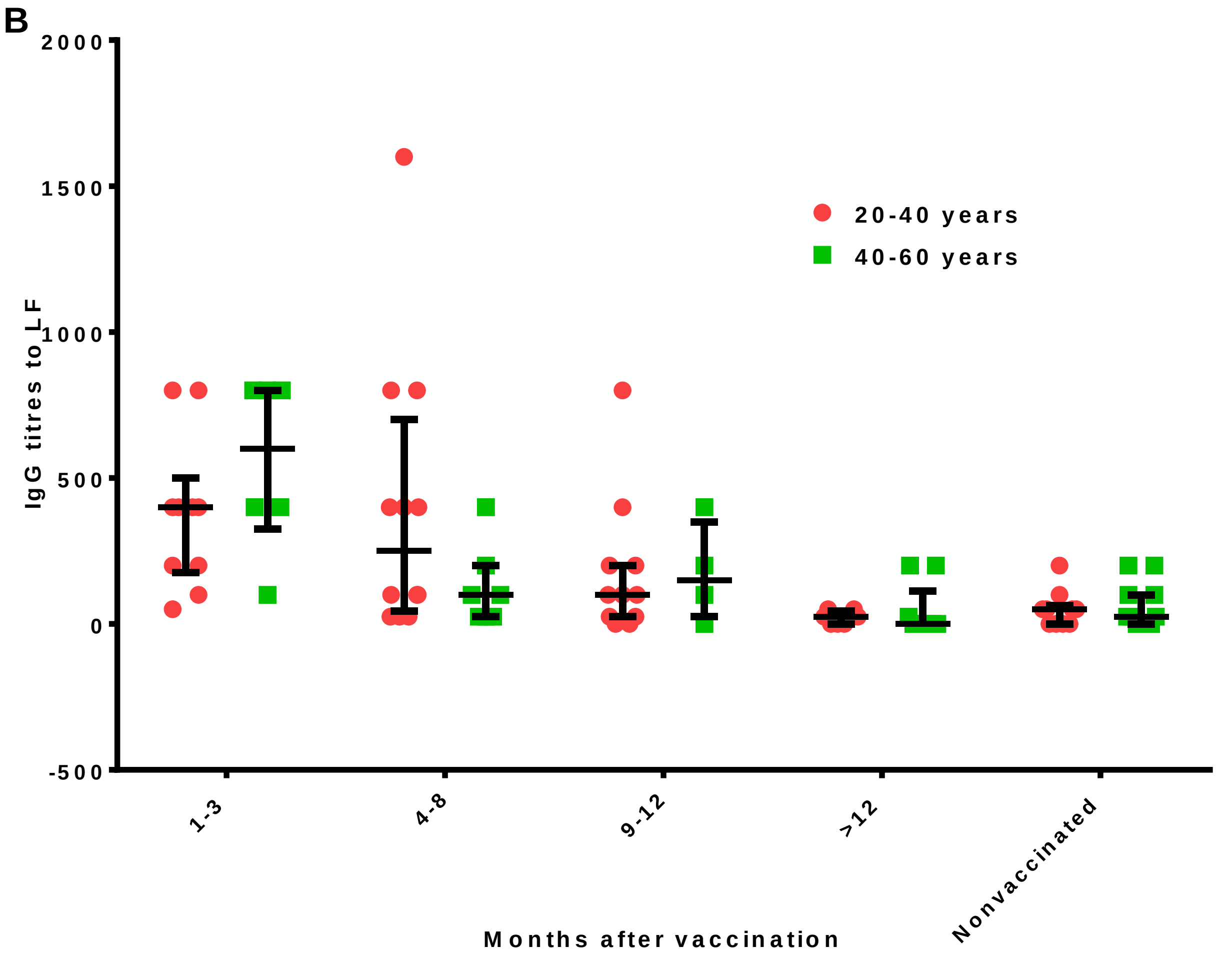

Supplement: S17 Dataset — (PDF) [file pone.0260202.s032.pdf]
