# Supplementary material for: Characterization of the adaptive immune response of donors receiving live anthrax vaccine
Source: PLoS One. 2021 Dec 20;16(12):e0260202. doi: 10.1371/journal.pone.0260202 (PMC8687594; doi:10.1371/journal.pone.0260202)

Analysis of the effect of age on the development and duration of anti-anthrax post-vaccination immunity (Age vs. Spores titers).

Statistical analysis was performed using a Two-way ANOVA with Tukey's multiple comparison (determination of significance and confidence intervals). The histograms show the mean and the confidence interval (CI) as an interval estimate of the general frame.

	**Months after Vaccination**				
	**1-3**	**4-8**	**9-11**	**>12**	**Nonvaccinated**
**Titers in the group ages 20-40**	200	200	0	200	200
	200	400	200	400	0
	400	1600	800	400	50
	800	800	400	200	100
	1600	1600	200	200	800
	1600	200	200	0	400
	800	200	0	400	50
	400	100	25	200	50
	1600	800	100		25
	400	50	100		0
		400	100		
		200			
**Titers in the group ages 40-60**	1600	400	100	200	100
	1600	400	50	200	0
	3200	800	200	100	200
	3200	3200	400	0	50
	800	100		200	0
	800	400		1600	0
		200		200	100
				800	50
				0	25
					0
					100

**Two-Way ANOVA**						
**Table Analyzed**	**Age vs. Spores titers**					
	**Ordinary**					
**Alpha**	0,05					
**Source of Variation**	**% of total variation**	**P value**	**P value summary**	**Significant?**		
**Interaction**	5,43	0,1513	ns	No		
**Row Factor**	34,33	< 0,0001	****	Yes		
**Column Factor**	2,019	0,1124	ns	No		
**ANOVA table**	SS	DF	MS	F (DFn, DFd)	P value	
**Interaction**	2220000	4	555003	F (4, 77) = 1,733	P = 0,1513	
**Row Factor**	14030000	4	3508000	F (4, 77) = 10,96	P < 0,0001	
**Column Factor**	825600	1	825600	F (1, 77) = 2,578	P = 0,1124	
**Residual**	24660000	77	320222			
**Number of missing values**	33					

ANOVA Multiple Comparison								
**Number of families**	1							
**Number of comparisons per family**	10							
**Alpha**	0,05							
**Tukey's multiple comparisons test**	**Mean Diff,**	**95% CI of diff,**	**Significant?**	**Summary**				
20-40 years								
**1-3 vs. 4-8**	298,6	-398,4 to 995,7	No	ns				
**1-3 vs. 9-12**	641,9	-84,37 to 1368	No	ns				
**1-3 vs. >12**	594,4	-173,7 to 1363	No	ns				
**1-3 vs. Nonvaccinated**	658,3	-86,85 to 1404	No	ns				
**4-8 vs. 9-12**	343,3	-333,5 to 1020	No	ns				
**4-8 vs. >12**	295,8	-425,7 to 1017	No	ns				
**4-8 vs. Nonvaccinated**	359,7	-337,3 to 1057	No	ns				
**9-12 vs. >12**	-47,5	-797,3 to 702,3	No	ns				
**9-12 vs. Nonvaccinated**	16,39	-709,9 to 742,7	No	ns				
**>12 vs. Nonvaccinated**	63,89	-704,2 to 832,0	No	ns				
40-60 years								
**1-3 vs. 4-8**	871,4	26,47 to 1716	Yes	*				
**1-3 vs. 9-12**	1470	478,8 to 2460	Yes	***				
**1-3 vs. >12**	1290	493,8 to 2087	Yes	***				
**1-3 vs. Nonvaccinated**	1605	853,3 to 2357	Yes	****				
**4-8 vs. 9-12**	598,2	-392,6 to 1589	No	ns				
**4-8 vs. >12**	419	-377,6 to 1216	No	ns				
**4-8 vs. Nonvaccinated**	733,6	-18,18 to 1485	No	ns				
**9-12 vs. >12**	-179,2	-1129 to 770,8	No	ns				
**9-12 vs. Nonvaccinated**	135,4	-777,2 to 1048	No	ns				
**>12 vs. Nonvaccinated**	314,6	-382,5 to 1012	No	ns				
**Test details**	**Mean 1**	**Mean 2**	**Mean Diff,**	**SE of diff,**	**N1**	**N2**	**q**	**DF**
20-40 years								
**1-3 vs. 4-8**	844,4	545,8	298,6	249,5	9	12	1,692	77
**1-3 vs. 9-11**	844,4	202,5	641,9	260	9	10	3,492	77
**1-3 vs. >12**	844,4	250	594,4	275	9	8	3,057	77
**1-3 vs. Nonvaccinated**	844,4	186,1	658,3	266,8	9	9	3,49	77
**4-8 vs. 9-11**	545,8	202,5	343,3	242,3	12	10	2,004	77
**4-8 vs. >12**	545,8	250	295,8	258,3	12	8	1,62	77
**4-8 vs. Nonvaccinated**	545,8	186,1	359,7	249,5	12	9	2,039	77
**9-11 vs. >12**	202,5	250	-47,5	268,4	10	8	0,2503	77

**9-11 vs. Nonvaccinated**	202,5	186,1	16,39	260	10	9	0,08914	77
**>12 vs. Nonvaccinated**	250	186,1	63,89	275	8	9	0,3286	77
40-60 years								
**1-3 vs. 4-8**	1657	785,7	871,4	302,5	7	7	4,074	77
**1-3 vs. 9-11**	1657	187,5	1470	354,7	7	4	5,86	77
**1-3 vs. >12**	1657	366,7	1290	285,2	7	9	6,4	77
**1-3 vs. Nonvaccinated**	1657	52,08	1605	269,1	7	12	8,434	77
**4-8 vs. 9-11**	785,7	187,5	598,2	354,7	7	4	2,385	77
**4-8 vs. >12**	785,7	366,7	419	285,2	7	9	2,078	77
**4-8 vs. Nonvaccinated**	785,7	52,08	733,6	269,1	7	12	3,855	77
**9-11 vs. >12**	187,5	366,7	-179,2	340,1	4	9	0,7451	77
**9-11 vs. Nonvaccinated**	187,5	52,08	135,4	326,7	4	12	0,5862	77
**>12 vs. Nonvaccinated**	366,7	52,08	314,6	249,5	9	12	1,783	77

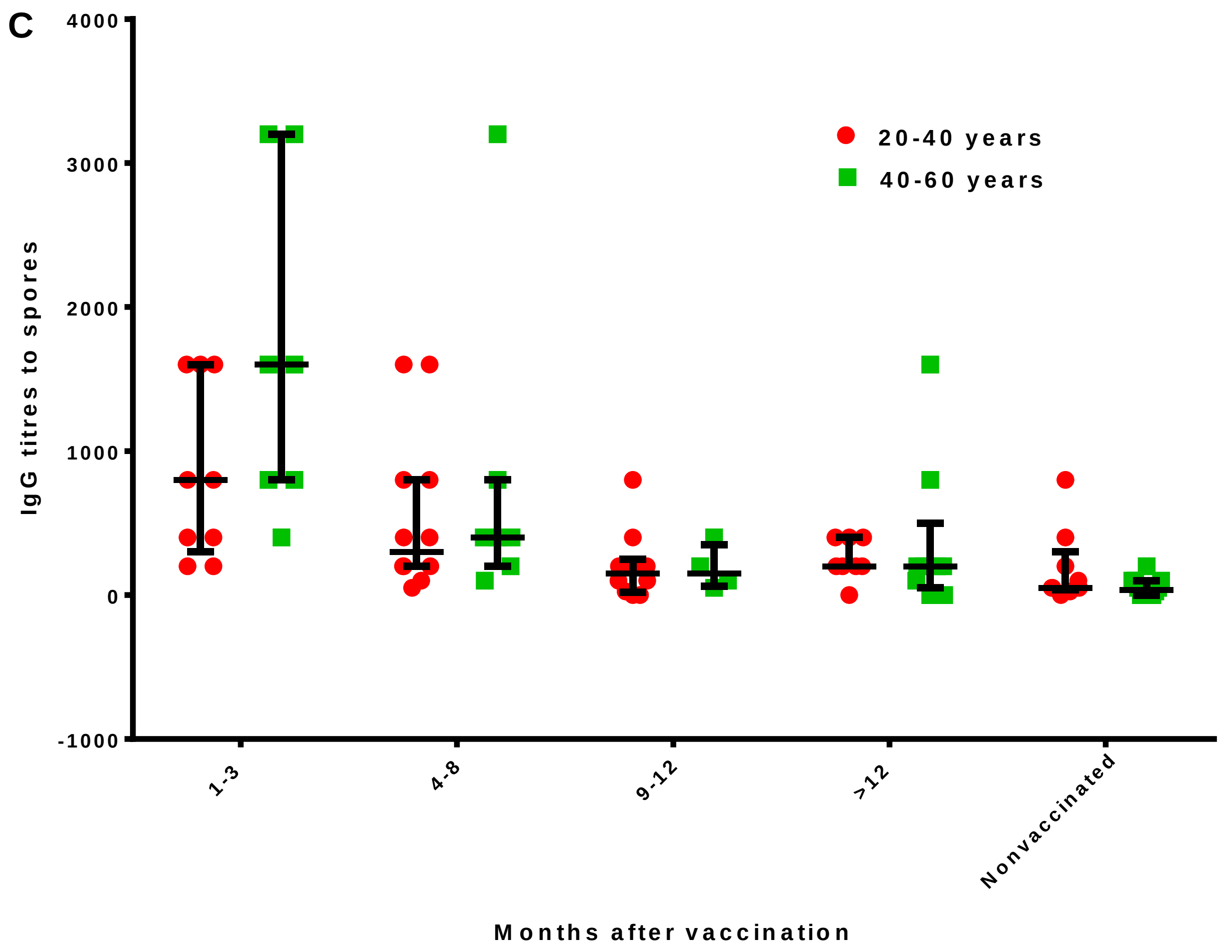

Supplement: S18 Dataset — (PDF) [file pone.0260202.s033.pdf]
