# Supplementary material for: Characterization of the adaptive immune response of donors receiving live anthrax vaccine
Source: PLoS One. 2021 Dec 20;16(12):e0260202. doi: 10.1371/journal.pone.0260202 (PMC8687594; doi:10.1371/journal.pone.0260202)

Analysis of the effect of age on the development and duration of anti-anthrax post-vaccination immunity (Age vs. TNA).

Statistical analysis was performed using a Two-way ANOVA with Tukey's multiple comparison (determination of significance and confidence intervals). The histograms show the mean and the confidence interval (CI) as an interval estimate of the general frame.

	**Months after Vaccination**				
	**1-3**	**4-8**	**9-11**	**>12**	**Nonvaccinated**
**Viability (%) in TNA assay for the serum in the group ages 20-40**	31	20	12	31	23
	39	42	39	12	12
	48	38	26	26	17
	50	39	79	48	14
	43	45	44	21	21
	53	29	37	26	12
	72	23	17	5	10
	30	37	14	30	16
	22	50	51		9
	37	34	41		17
		18	29		
		33			
**Viability (%) in TNA assay for the serum in the group ages 40-60**	40	26	41	28	14
	56	65	37	70	8
	51	19	42	40	13
	69	42	57	43	9
	27	34		29	7
	43	52		26	14
		16		26	7
				58	12
				14	12
					11
					9

**Two-Way ANOVA**						
**Table Analyzed**	**Age vs. TNA**					
	**Ordinary**					
**Alpha**	0,05					
**Source of Variation**	**% of total variation**	**P value**	**P value summary**	**Significant?**		
**Interaction**	2,909	0,3778	ns	No		
**Row Factor**	43,61	< 0,0001	****	Yes		
**Column Factor**	1,765	0,1114	ns	No		
**ANOVA table**	SS	DF	MS	F (DFn, DFd)	P value	
**Interaction**	748	4	187	F (4, 78) = 1,069	P = 0,3778	
**Row Factor**	11213	4	2803	F (4, 78) = 16,02	P < 0,0001	
**Column Factor**	453,7	1	453,7	F (1, 78) = 2,593	P = 0,1114	
**Residual**	13649	78	175			
**Number of missing values**	32					

ANOVA Multiple Comparison

**Number of families**	1							
**Number of comparisons per family**	10							
**Alpha**	0,05							
**Tukey's multiple comparisons test**	**Mean Diff,**	**95% CI of diff,**	**Significant?**	**Summary**				
20-40 years								
**1-3 vs. 4-8**	8,5	-7,317 to 24,32	No	ns				
**1-3 vs. 9-12**	7,199	-8,942 to 23,34	No	ns				
**1-3 vs. >12**	17,73	0,2048 to 35,25	Yes	*				
**1-3 vs. Nonvaccinated**	27,58	11,06 to 44,10	Yes	***				
**4-8 vs. 9-12**	-1,301	-16,72 to 14,12	No	ns				
**4-8 vs. >12**	9,228	-7,634 to 26,09	No	ns				
**4-8 vs. Nonvaccinated**	19,08	3,260 to 34,89	Yes	*				
**9-12 vs. >12**	10,53	-6,636 to 27,69	No	ns				
**9-12 vs. Nonvaccinated**	20,38	4,238 to 36,52	Yes	**				
**>12 vs. Nonvaccinated**	9,85	-7,673 to 27,37	No	ns				
40-60 years								
**1-3 vs. 4-8**	11,38	-9,171 to 31,93	No	ns				
**1-3 vs. 9-12**	3,543	-20,30 to 27,39	No	ns				
**1-3 vs. >12**	10,74	-8,734 to 30,21	No	ns				
**1-3 vs. Nonvaccinated**	37,26	18,51 to 56,01	Yes	****				
**4-8 vs. 9-12**	-7,838	-30,99 to 15,32	No	ns				
**4-8 vs. >12**	-0,6452	-19,26 to 17,97	No	ns				
**4-8 vs. Nonvaccinated**	25,88	8,017 to 43,74	Yes	**				
**9-12 vs. >12**	7,193	-15,01 to 29,39	No	ns				
**9-12 vs. Nonvaccinated**	33,72	12,15 to 55,29	Yes	***				
**>12 vs. Nonvaccinated**	26,52	9,919 to 43,13	Yes	***				
**Test details**	**Mean 1**	**Mean 2**	**Mean Diff,**	**SE of diff,**	**N1**	**N2**	**q**	**DF**
20-40 years								
**1-3 vs. 4-8**	42,5	34	8,5	5,664	10	12	2,122	78
**1-3 vs. 9-11**	42,5	35,3	7,199	5,78	10	11	1,761	78
**1-3 vs. >12**	42,5	24,77	17,73	6,275	10	8	3,996	78
**1-3 vs. Nonvaccinated**	42,5	14,92	27,58	5,916	10	10	6,592	78
**4-8 vs. 9-11**	34	35,3	-1,301	5,522	12	11	0,3333	78
**4-8 vs. >12**	34	24,77	9,228	6,038	12	8	2,161	78
**4-8 vs. Nonvaccinated**	34	14,92	19,08	5,664	12	10	4,763	78
**9-11 vs. >12**	35,3	24,77	10,53	6,147	11	8	2,422	78

**9-11 vs. Nonvaccinated**	35,3	14,92	20,38	5,78	11	10	4,986	78
**>12 vs. Nonvaccinated**	24,77	14,92	9,85	6,275	8	10	2,22	78
40-60 years								
**1-3 vs. 4-8**	47,67	36,29	11,38	7,359	6	7	2,187	78
**1-3 vs. 9-11**	47,67	44,12	3,543	8,539	6	4	0,5868	78
**1-3 vs. >12**	47,67	36,93	10,74	6,972	6	9	2,178	78
**1-3 vs. Nonvaccinated**	47,67	10,41	37,26	6,714	6	11	7,849	78
**4-8 vs. 9-11**	36,29	44,12	-7,838	8,291	7	4	1,337	78
**4-8 vs. >12**	36,29	36,93	-0,6452	6,666	7	9	0,1369	78
**4-8 vs. Nonvaccinated**	36,29	10,41	25,88	6,396	7	11	5,722	78
**9-11 vs. >12**	44,12	36,93	7,193	7,949	4	9	1,28	78
**9-11 vs. Nonvaccinated**	44,12	10,41	33,72	7,724	4	11	6,174	78
**>12 vs. Nonvaccinated**	36,93	10,41	26,52	5,946	9	11	6,309	78

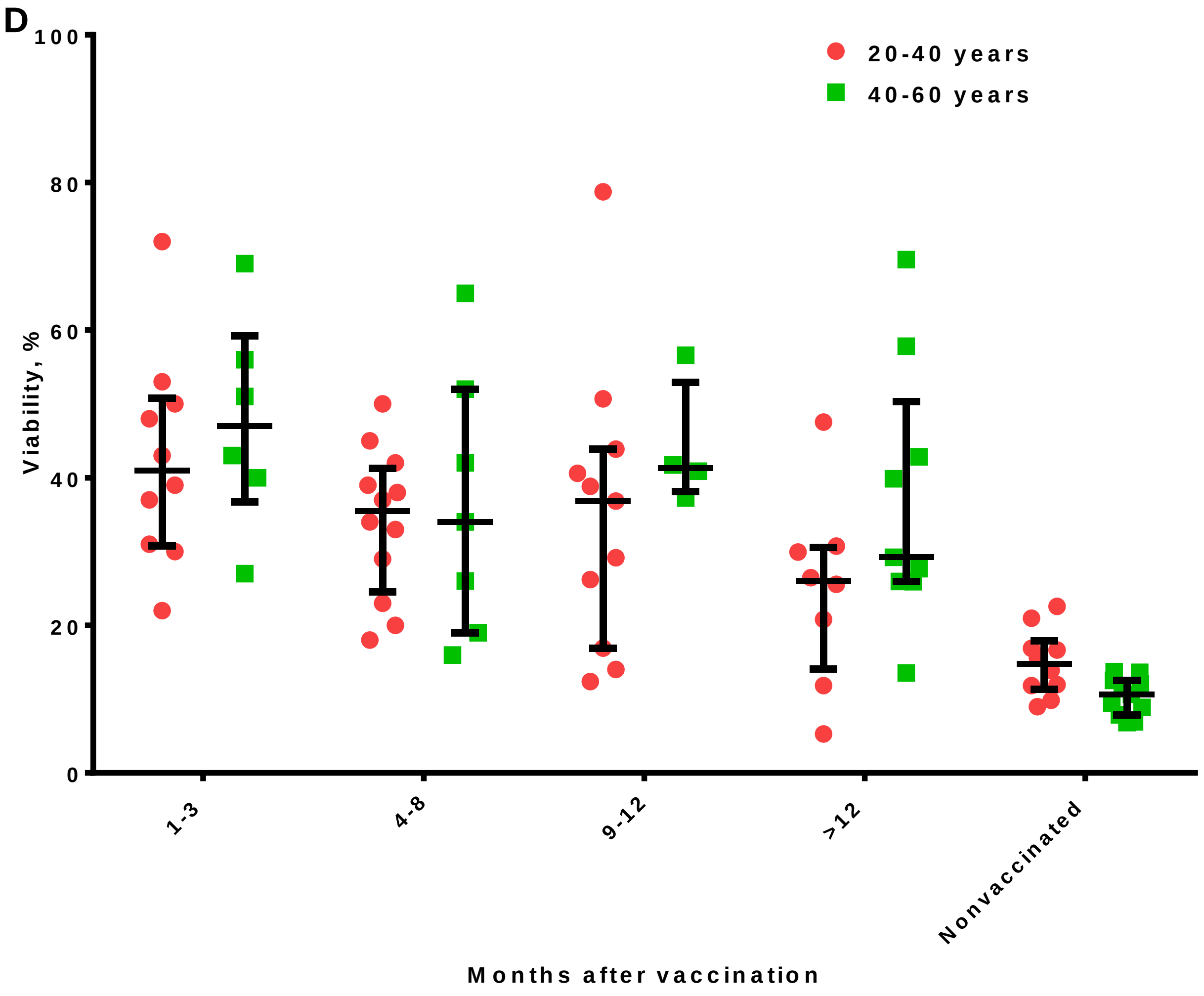

Supplement: S19 Dataset — (PDF) [file pone.0260202.s034.pdf]
