# Supplementary material for: Characterization of the adaptive immune response of donors receiving live anthrax vaccine
Source: PLoS One. 2021 Dec 20;16(12):e0260202. doi: 10.1371/journal.pone.0260202 (PMC8687594; doi:10.1371/journal.pone.0260202)

The effect of gender on the development of anti-anthrax post-vaccination immunity and the duration of circulation of IgG to PA.

Statistical analysis was performed using a Two-way ANOVA with Tukey's multiple comparison (determination of significance and confidence intervals). The histograms show the mean and the confidence interval as an interval estimate of the general frame.

	Months after Vaccination				
	1-3	4-8	9-11	>12	Nonvaccinated
Titers in the group of men	200	800	100	100	100
	800	400	1600	50	400
	800	400	200	50	200
	100	100	0	200	400
	800	25	50	0	0
	400	25	25	0	50
	100	0	400	25	100
	800	100	200	0	25
	400	100			50
	800	0			0
Titers in the group of women	100	25	0	50	0
	800	400	400	100	0
	1600	800	50	200	50
	1600	400	200	100	200
	800	25	100	400	400
	400	100	50	25	0
		50	100	100	400
		100		25	100
		400		100	50
					25
					200

**Two-Way ANOVA**						
**Table Analyzed**	**Gender vs. PA titers**					
	**Ordinary**					
**Alpha**	0,05					
**Source of Variation**	**% of total variation**	**P value**	**P value summary**	**Significant?**		
**Interaction**	5,672	0,1401	ns	No		
**Row Factor**	35,78	< 0,0001	****	Yes		
**Column Factor**	0,7036	0,3494	ns	No		
**ANOVA table**	SS	DF	MS	F (DFn, DFd)	P value	
**Interaction**	605263	4	151316	F (4, 78) = 1,786	P = 0,1401	
**Row Factor**	3818000	4	954559	F (4, 78) = 11,27	P < 0,0001	
**Column Factor**	75084	1	75084	F (1, 78) = 0,8862	P = 0,3494	
**Residual**	6608000	78	84722			
**Number of missing values**	22					

ANOVA Multiple Comparison								
**Number of families**	1							
**Number of comparisons per family**	10							
**Alpha**	0,05							
**Tukey's multiple comparisons test**	**Mean Diff,**	**95% CI of diff,**	**Significant?**	**Summary**				
Men								
**1-3 vs. 4-8**	325	-38,52 to 688,5	No	ns				
**1-3 vs. 9-12**	198,1	-187,4 to 583,7	No	ns				
**1-3 vs. >12**	466,9	81,31 to 852,4	Yes	**				
**1-3 vs. Nonvaccinated**	387,5	23,98 to 751,0	Yes	*				
**4-8 vs. 9-12**	-126,9	-512,4 to 258,7	No	ns				
**4-8 vs. >12**	141,9	-243,7 to 527,4	No	ns				
**4-8 vs. Nonvaccinated**	62,5	-301,0 to 426,0	No	ns				
**9-12 vs. >12**	268,8	-137,7 to 675,2	No	ns				
**9-12 vs. Nonvaccinated**	189,4	-196,2 to 574,9	No	ns				
**>12 vs. Nonvaccinated**	-79,38	-464,9 to 306,2	No	ns				
Woman								
**1-3 vs. 4-8**	627,8	199,4 to 1056	Yes	***				
**1-3 vs. 9-12**	754,8	302,5 to 1207	Yes	***				
**1-3 vs. >12**	761,1	332,7 to 1190	Yes	****				
**1-3 vs. Nonvaccinated**	753,8	341,3 to 1166	Yes	****				
**4-8 vs. 9-12**	127	-282,7 to 536,6	No	ns				
**4-8 vs. >12**	133,3	-249,8 to 516,5	No	ns				
**4-8 vs. Nonvaccinated**	126	-239,3 to 491,4	No	ns				
**9-12 vs. >12**	6,349	-403,3 to 416,0	No	ns				
**9-12 vs. Nonvaccinated**	-0,974	-394,0 to 392,0	No	ns				
**>12 vs. Nonvaccinated**	-7,323	-372,7 to 358,0	No	ns				
**Test details**	**Mean 1**	**Mean 2**	**Mean Diff,**	**SE of diff,**	**N1**	**N2**	**q**	**DF**
Men								
**1-3 vs. 4-8**	520	195	325	130,2	10	10	3,531	78
**1-3 vs. 9-11**	520	321,9	198,1	138,1	10	8	2,029	78
**1-3 vs. >12**	520	53,13	466,9	138,1	10	8	4,782	78
**1-3 vs. Nonvaccinated**	520	132,5	387,5	130,2	10	10	4,21	78
**4-8 vs. 9-11**	195	321,9	-126,9	138,1	10	8	1,3	78
**4-8 vs. >12**	195	53,13	141,9	138,1	10	8	1,453	78
**4-8 vs. Nonvaccinated**	195	132,5	62,5	130,2	10	10	0,679	78
**9-11 vs. >12**	321,9	53,13	268,8	145,5	8	8	2,612	78

**9-11 vs. Nonvaccinated**	321,9	132,5	189,4	138,1	8	10	1,94	78
**>12 vs. Nonvaccinated**	53,13	132,5	-79,38	138,1	8	10	0,813	78
Woman								
**1-3 vs. 4-8**	883,3	255,6	627,8	153,4	6	9	5,787	78
**1-3 vs. 9-11**	883,3	128,6	754,8	161,9	6	7	6,591	78
**1-3 vs. >12**	883,3	122,2	761,1	153,4	6	9	7,016	78
**1-3 vs. Nonvaccinated**	883,3	129,5	753,8	147,7	6	11	7,216	78
**4-8 vs. 9-11**	255,6	128,6	127	146,7	9	7	1,224	78
**4-8 vs. >12**	255,6	122,2	133,3	137,2	9	9	1,374	78
**4-8 vs. Nonvaccinated**	255,6	129,5	126	130,8	9	11	1,362	78
**9-11 vs. >12**	128,6	122,2	6,349	146,7	7	9	0,06121	78
**9-11 vs. Nonvaccinated**	128,6	129,5	-0,974	140,7	7	11	0,009788	78
**>12 vs. Nonvaccinated**	122,2	129,5	-7,323	130,8	9	11	0,07916	78

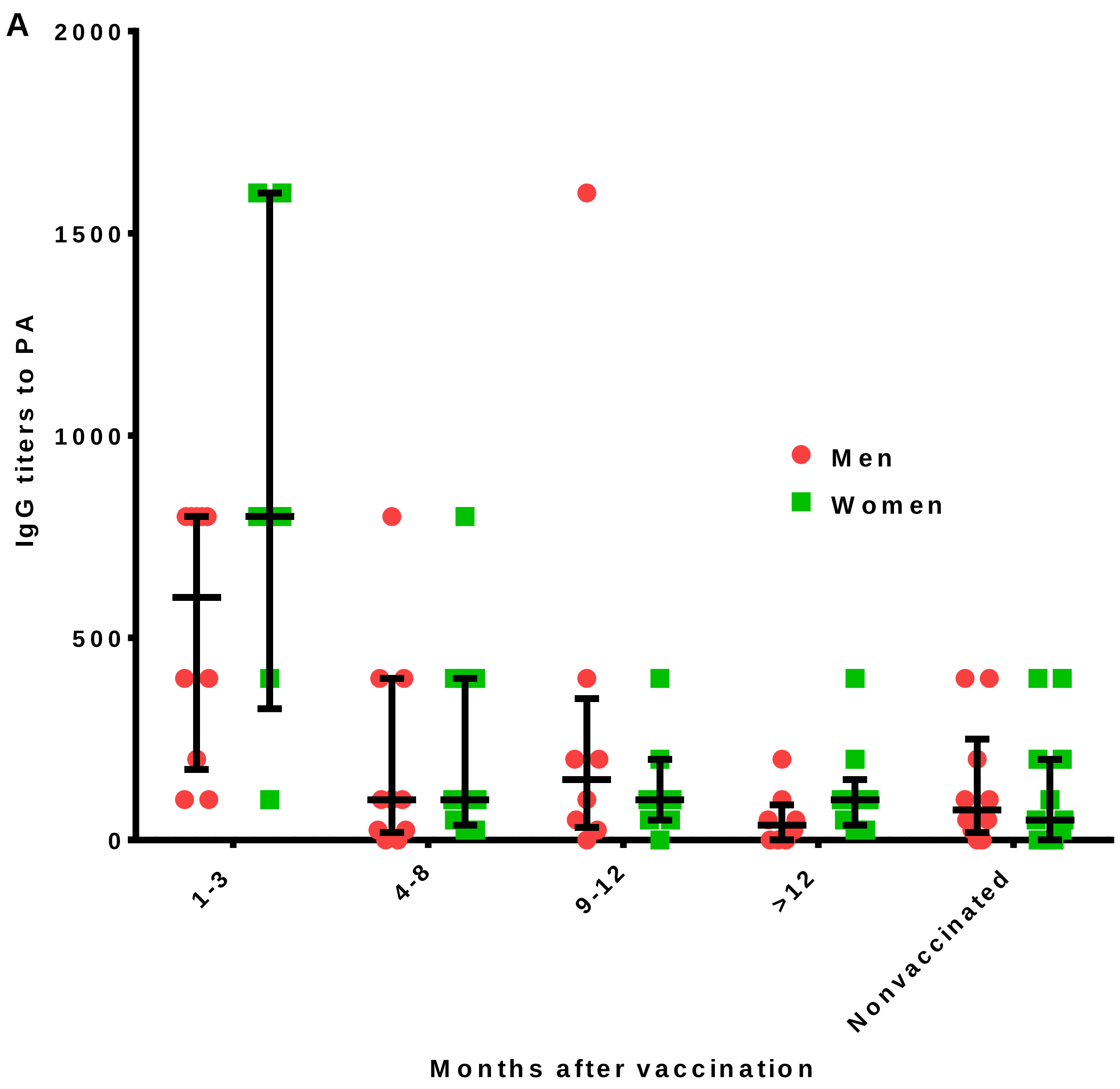

Supplement: S20 Dataset — (PDF) [file pone.0260202.s035.pdf]
