# Supplementary material for: Characterization of the adaptive immune response of donors receiving live anthrax vaccine
Source: PLoS One. 2021 Dec 20;16(12):e0260202. doi: 10.1371/journal.pone.0260202 (PMC8687594; doi:10.1371/journal.pone.0260202)

**The effect of gender on the development of anti-anthrax post-vaccination immunity and the duration of circulation of IgG to LF.**

Statistical analysis was performed using a Two-way ANOVA with Tukey's multiple comparison (determination of significance and confidence intervals). The histograms show the mean and the confidence interval as an interval estimate of the general frame.

| | Months after Vaccination | | | | |
|---|---|---|---|---|---|
| | **1-3** | **4-8** | **9-11** | **>12** | **Nonvaccinated** |
| **Titers in the group of men** | 200 | 100 | 200 | 0 | 50 |
| | 800 | 100 | 800 | 0 | 200 |
| | 100 | 200 | 100 | 0 | 50 |
| | 200 | 25 | 0 | 0 | 50 |
| | 800 | 25 | 100 | 25 | 100 |
| | 800 | 100 | 25 | 50 | 50 |
| | 400 | 25 | 200 | 25 | 0 |
| | 50 | 100 | 100 | 25 | 0 |
| | 400 | 25 | | | 0 |
| | 400 | 400 | | | 0 |
| **Titers in the group of women** | 400 | 1600 | 0 | 0 | 25 |
| | 400 | 400 | 400 | 0 | 25 |
| | 800 | 800 | 400 | 200 | 200 |
| | 800 | 400 | 200 | 0 | 0 |
| | 100 | 800 | 100 | 200 | 25 |
| | 400 | 25 | 0 | 50 | 0 |
| | | 25 | 25 | 0 | 0 |
| | | 100 | | 0 | 100 |
| | | 400 | | 25 | 200 |
| | | | | | 100 |
| | | | | | 0 |

| **Two-Way ANOVA** | | | | | | |
|---|---|---|---|---|---|---|
| **Table Analyzed** | **Gender vs. LF titers** | | | | | |
| | | | | | | |
| | **Ordinary** | | | | | |
| **Alpha** | 0,05 | | | | | |
| | | | | | | |
| **Source of Variation** | **% of total variation** | **P value** | **P value summary** | **Significant?** | | |
| **Interaction** | 7,83 | 0,0449 | * | Yes | | |
| **Row Factor** | 29,97 | < 0,0001 | **** | Yes | | |
| **Column Factor** | 2,888 | 0,0555 | ns | No | | |
| | | | | | | |
| **ANOVA table** | SS | DF | MS | F (DFn, DFd) | P value | |
| **Interaction** | 538226 | 4 | 134557 | F (4, 78) = 2,562 | P = 0,0449 | |
| **Row Factor** | 2060000 | 4 | 515072 | F (4, 78) = 9,807 | P < 0,0001 | |
| **Column Factor** | 198525 | 1 | 198525 | F (1, 78) = 3,780 | P = 0,0555 | |
| **Residual** | 4097000 | 78 | 52522 | | | |
| | | | | | | |
| **Number of missing values** | 22 | | | | | |

| ANOVA Multiple Comparison | | | | | | | | |
|---|---|---|---|---|---|---|---|---|
| | | | | | | | | |
| **Number of families** | 1 | | | | | | | |
| **Number of comparisons per family** | 10 | | | | | | | |
| **Alpha** | 0,05 | | | | | | | |
| | | | | | | | | |
| **Tukey's multiple comparisons test** | **Mean Diff,** | **95% CI of diff,** | **Significant?** | **Summary** | | | | |
| | | | | | | | | |
| ***Men*** | | | | | | | | |
| **1-3 vs. 4-8** | 305 | 18,78 to 591,2 | Yes | * | | | | |
| **1-3 vs. 9-12** | 224,4 | -79,21 to 528,0 | No | ns | | | | |
| **1-3 vs. >12** | 399,4 | 95,79 to 703,0 | Yes | ** | | | | |
| **1-3 vs. Nonvaccinated** | 365 | 78,78 to 651,2 | Yes | ** | | | | |
| **4-8 vs. 9-12** | -80,63 | -384,2 to 223,0 | No | ns | | | | |
| **4-8 vs. >12** | 94,38 | -209,2 to 398,0 | No | ns | | | | |
| **4-8 vs. Nonvaccinated** | 60 | -226,2 to 346,2 | No | ns | | | | |
| **9-12 vs. >12** | 175 | -145,0 to 495,0 | No | ns | | | | |
| **9-12 vs. Nonvaccinated** | 140,6 | -163,0 to 444,2 | No | ns | | | | |
| **>12 vs. Nonvaccinated** | -34,38 | -338,0 to 269,2 | No | ns | | | | |
| | | | | | | | | |
| ***Women*** | | | | | | | | |
| **1-3 vs. 4-8** | -22,22 | -359,5 to 315,1 | No | ns | | | | |
| **1-3 vs. 9-12** | 322,6 | -33,45 to 678,7 | No | ns | | | | |
| **1-3 vs. >12** | 430,6 | 93,24 to 767,9 | Yes | ** | | | | |
| **1-3 vs. Nonvaccinated** | 422 | 97,16 to 746,8 | Yes | ** | | | | |
| **4-8 vs. 9-12** | 344,8 | 22,31 to 667,4 | Yes | * | | | | |
| **4-8 vs. >12** | 452,8 | 151,1 to 754,5 | Yes | *** | | | | |
| **4-8 vs. Nonvaccinated** | 444,2 | 156,5 to 731,9 | Yes | *** | | | | |
| **9-12 vs. >12** | 107,9 | -214,6 to 430,5 | No | ns | | | | |
| **9-12 vs. Nonvaccinated** | 99,35 | -210,1 to 408,8 | No | ns | | | | |
| **>12 vs. Nonvaccinated** | -8,586 | -296,2 to 279,1 | No | ns | | | | |
| | | | | | | | | |
| **Test details** | **Mean 1** | **Mean 2** | **Mean Diff,** | **SE of diff,** | **N1** | **N2** | **q** | **DF** |
| | | | | | | | | |
| ***Men*** | | | | | | | | |
| **1-3 vs. 4-8** | 415 | 110 | 305 | 102,5 | 10 | 10 | 4,209 | 78 |
| **1-3 vs. 9-11** | 415 | 190,6 | 224,4 | 108,7 | 10 | 8 | 2,919 | 78 |
| **1-3 vs. >12** | 415 | 15,63 | 399,4 | 108,7 | 10 | 8 | 5,196 | 78 |
| **1-3 vs. Nonvaccinated** | 415 | 50 | 365 | 102,5 | 10 | 10 | 5,036 | 78 |
| **4-8 vs. 9-11** | 110 | 190,6 | -80,63 | 108,7 | 10 | 8 | 1,049 | 78 |
| **4-8 vs. >12** | 110 | 15,63 | 94,38 | 108,7 | 10 | 8 | 1,228 | 78 |
| **4-8 vs. Nonvaccinated** | 110 | 50 | 60 | 102,5 | 10 | 10 | 0,8279 | 78 |
| **9-11 vs. >12** | 190,6 | 15,63 | 175 | 114,6 | 8 | 8 | 2,16 | 78 |

| | | | | | | | | |
|---|---|---|---|---|---|---|---|---|
| **9-11 vs. Nonvaccinated** | 190,6 | 50 | 140,6 | 108,7 | 8 | 10 | 1,829 | 78 |
| **>12 vs. Nonvaccinated** | 15,63 | 50 | -34,38 | 108,7 | 8 | 10 | 0,4472 | 78 |
| | | | | | | | | |
| ***Women*** | | | | | | | | |
| **1-3 vs. 4-8** | 483,3 | 505,6 | -22,22 | 120,8 | 6 | 9 | 0,2602 | 78 |
| **1-3 vs. 9-11** | 483,3 | 160,7 | 322,6 | 127,5 | 6 | 7 | 3,578 | 78 |
| **1-3 vs. >12** | 483,3 | 52,78 | 430,6 | 120,8 | 6 | 9 | 5,041 | 78 |
| **1-3 vs. Nonvaccinated** | 483,3 | 61,36 | 422 | 116,3 | 6 | 11 | 5,131 | 78 |
| **4-8 vs. 9-11** | 505,6 | 160,7 | 344,8 | 115,5 | 9 | 7 | 4,223 | 78 |
| **4-8 vs. >12** | 505,6 | 52,78 | 452,8 | 108 | 9 | 9 | 5,927 | 78 |
| **4-8 vs. Nonvaccinated** | 505,6 | 61,36 | 444,2 | 103 | 9 | 11 | 6,098 | 78 |
| **9-11 vs. >12** | 160,7 | 52,78 | 107,9 | 115,5 | 7 | 9 | 1,322 | 78 |
| **9-11 vs. Nonvaccinated** | 160,7 | 61,36 | 99,35 | 110,8 | 7 | 11 | 1,268 | 78 |
| **>12 vs. Nonvaccinated** | 52,78 | 61,36 | -8,586 | 103 | 9 | 11 | 0,1179 | 78 |

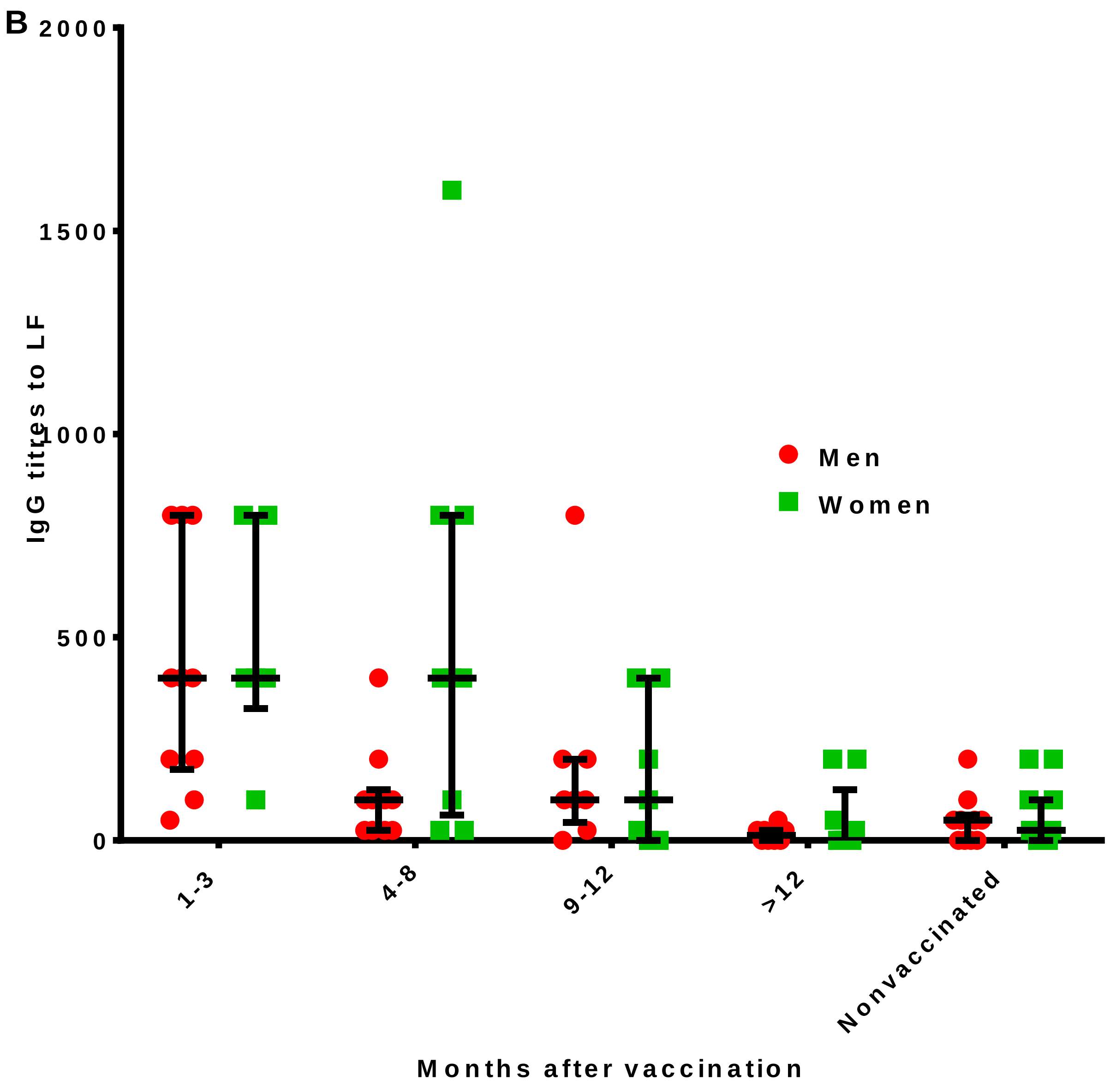

Supplement: S21 Dataset — (PDF) [file pone.0260202.s036.pdf]
