# Supplementary material for: Characterization of the adaptive immune response of donors receiving live anthrax vaccine
Source: PLoS One. 2021 Dec 20;16(12):e0260202. doi: 10.1371/journal.pone.0260202 (PMC8687594; doi:10.1371/journal.pone.0260202)

The effect of gender on the development of anti-anthrax post-vaccination immunity and the duration of circulation of IgG to spores.

Statistical analysis was performed using a Two-way ANOVA with Tukey's multiple comparison (determination of significance and confidence intervals). The histograms show the mean and the confidence interval as an interval estimate of the general frame.

	**Months after Vaccination**				
	**1-3**	**4-8**	**9-11**	**>12**	**Nonvaccinated**
**Titers in the group of men**	200	200	400	100	200
	1600	400	800	200	0
	400	400	400	400	50
	800	800	0	200	100
	1600	100	25	200	800
	800	100	100	400	400
	800	50	200	200	50
	400	400	400	0	50
	400	200			60
	3200	400			25
**Titers in the group of women**	200	1600	0	200	100
	1600	800	200	200	0
	3200	1600	100	1600	200
	1600	3200	200	200	50
	800	200	200	800	0
	1600	200	50	400	0
		400	100	0	100
		200		200	25
		800		0	0
					100
					0

**Two-Way ANOVA**						
**Table Analyzed**	**Gender vs. Spores titers**					
	**Ordinary**					
**Alpha**	0,05					
**Source of Variation**	**% of total variation**	**P value**	**P value summary**	**Significant?**		
**Interaction**	6,108	0,1011	ns	No		
**Row Factor**	34,61	< 0,0001	****	Yes		
**Column Factor**	2,401	0,0792	ns	No		
**ANOVA table**	SS	DF	MS	F (DFn, DFd)	P value	
**Interaction**	2,498e+006	4	624388	F (4, 78) = 2,011	P = 0,1011	
**Row Factor**	1,415e+007	4	3,538e+006	F (4, 78) = 11,40	P < 0,0001	
**Column Factor**	981953	1	981953	F (1, 78) = 3,163	P = 0,0792	
**Residual**	2,422e+007	78	310479			
**Number of missing values**	22					

ANOVA Multiple Comparison

**Number of families**	1							
**Number of comparisons per family**	10							
**Alpha**	0,05							
**Tukey's multiple comparisons test**	**Mean Diff,**	**95% CI of diff,**	**Significant?**	**Summary**				
Men								
**1-3 vs. 4-8**	715,0	19,11 to 1411	Yes	*				
**1-3 vs. 9-12**	729,4	-8,730 to 1467	No	ns				
**1-3 vs. >12**	807,5	69,39 to 1546	Yes	*				
**1-3 vs. Nonvaccinated**	847,5	151,6 to 1543	Yes	**				
**4-8 vs. 9-12**	14,38	-723,7 to 752,5	No	ns				
**4-8 vs. >12**	92,50	-645,6 to 830,6	No	ns				
**4-8 vs. Nonvaccinated**	132,5	-563,4 to 828,4	No	ns				
**9-12 vs. >12**	78,13	-699,9 to 856,2	No	ns				
**9-12 vs. Nonvaccinated**	118,1	-620,0 to 856,2	No	ns				
**>12 vs. Nonvaccinated**	40,00	-698,1 to 778,1	No	ns				
Woman								
**1-3 vs. 4-8**	500,0	-320,1 to 1320	No	ns				
**1-3 vs. 9-12**	1379	512,9 to 2244	Yes	***				
**1-3 vs. >12**	1100	279,9 to 1920	Yes	**				
**1-3 vs. Nonvaccinated**	1448	658,0 to 2237	Yes	****				
**4-8 vs. 9-12**	878,6	94,39 to 1663	Yes	*				
**4-8 vs. >12**	600,0	-133,5 to 1334	No	ns				
**4-8 vs. Nonvaccinated**	947,7	248,3 to 1647	Yes	**				
**9-12 vs. >12**	-278,6	-1063 to 505,6	No	ns				
**9-12 vs. Nonvaccinated**	69,16	-683,2 to 821,5	No	ns				
**>12 vs. Nonvaccinated**	347,7	-351,7 to 1047	No	ns				
**Test details**	**Mean 1**	**Mean 2**	**Mean Diff,**	**SE of diff,**	**N1**	**N2**	**q**	**DF**
Men								
**1-3 vs. 4-8**	1020	305,0	715,0	249,2	10	10	4,058	78
**1-3 vs. 9-11**	1020	290,6	729,4	264,3	10	8	3,903	78
**1-3 vs. >12**	1020	212,5	807,5	264,3	10	8	4,321	78
**1-3 vs. Nonvaccinated**	1020	172,5	847,5	249,2	10	10	4,810	78
**4-8 vs. 9-11**	305,0	290,6	14,38	264,3	10	8	0,07692	78
**4-8 vs. >12**	305,0	212,5	92,50	264,3	10	8	0,4949	78
**4-8 vs. Nonvaccinated**	305,0	172,5	132,5	249,2	10	10	0,7520	78
**9-11 vs. >12**	290,6	212,5	78,13	278,6	8	8	0,3966	78

**9-11 vs. Nonvaccinated**	290,6	172,5	118,1	264,3	8	10	0,6320	78
**>12 vs. Nonvaccinated**	212,5	172,5	40,00	264,3	8	10	0,2140	78
Woman								
**1-3 vs. 4-8**	1500	1000	500,0	293,7	6	9	2,408	78
**1-3 vs. 9-11**	1500	121,4	1379	310,0	6	7	6,289	78
**1-3 vs. >12**	1500	400,0	1100	293,7	6	9	5,297	78
**1-3 vs. Nonvaccinated**	1500	52,27	1448	282,8	6	11	7,240	78
**4-8 vs. 9-11**	1000	121,4	878,6	280,8	9	7	4,425	78
**4-8 vs. >12**	1000	400,0	600,0	262,7	9	9	3,230	78
**4-8 vs. Nonvaccinated**	1000	52,27	947,7	250,4	9	11	5,352	78
**9-11 vs. >12**	121,4	400,0	-278,6	280,8	7	9	1,403	78
**9-11 vs. Nonvaccinated**	121,4	52,27	69,16	269,4	7	11	0,3630	78
**>12 vs. Nonvaccinated**	400,0	52,27	347,7	250,4	9	11	1,964	78

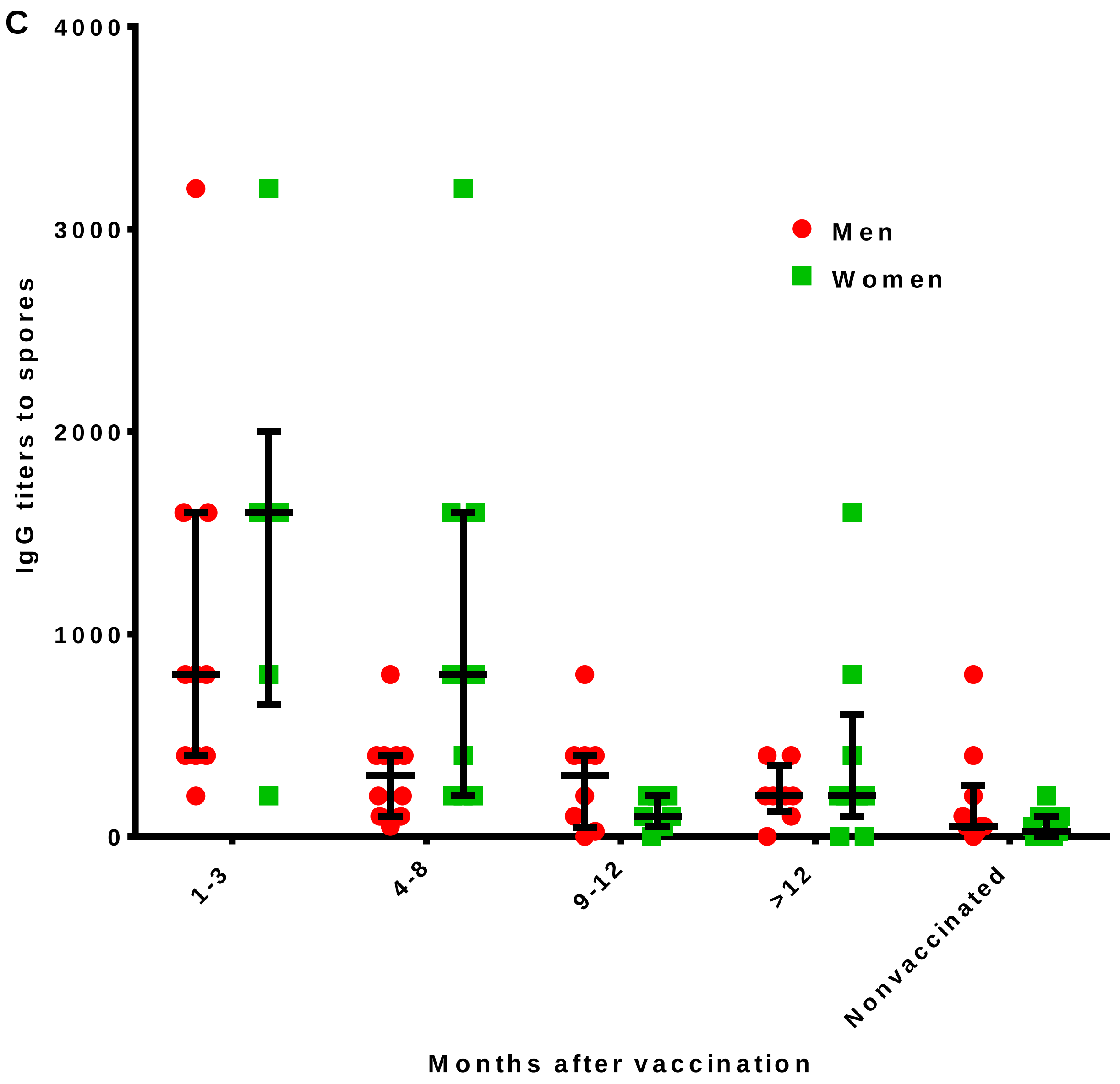

Supplement: S22 Dataset — (PDF) [file pone.0260202.s037.pdf]
