# Supplementary material for: Characterization of the adaptive immune response of donors receiving live anthrax vaccine
Source: PLoS One. 2021 Dec 20;16(12):e0260202. doi: 10.1371/journal.pone.0260202 (PMC8687594; doi:10.1371/journal.pone.0260202)

The effect of gender on the development of anti-anthrax post-vaccination immunity and TNA.

Statistical analysis was performed using a Two-way ANOVA with Tukey's multiple comparison (determination of significance and confidence intervals). The histograms show the mean and the confidence interval as an interval estimate of the general frame.

	**Months after Vaccination**				
	**1-3**	**4-8**	**9-11**	**>12**	**Nonvaccinated**
**TNA in the group of men Viability, %**	32	21	26	40	23
	41	27	79	31	12
	49	66	44	12	17
	51	20	14	48	14
	73	35	51	21	21
	70	37	41	5	12
	30	34	42	30	10
	23	19	57	26	16
	44	33			9
	54	43			17
**TNA in the group of women Viability, %**	39	39	12	28	14
	60	40	39	29	8
	51	45	40	25	12
	44	42	37	26	9
	28	30	17	58	7
	37	23	37	26	14
		53	29	14	7
		17		43	12
		51		70	12
					11
					9

Two-Way ANOVA						
**Table Analyzed**	**Gender vs. TNA**					
	**Ordinary**					
**Alpha**	0,05					
**Source of Variation**	**% of total variation**	**P value**	**P value summary**	**Significant?**		
**Interaction**	4,649	0,1461	ns	No		
**Row Factor**	41,52	< 0,0001	****	Yes		
**Column Factor**	0,2352	0,5528	ns	No		
**ANOVA table**	SS	DF	MS	F (DFn, DFd)	P value	
**Interaction**	1224	4	306,0	F (4, 78) = 1,757	P = 0,1461	
**Row Factor**	10931	4	2733	F (4, 78) = 15,69	P < 0,0001	
**Column Factor**	61,91	1	61,91	F (1, 78) = 0,3554	P = 0,5528	
**Residual**	13588	78	174,2			
**Number of missing values**	22					

ANOVA Multiple Comparison								
**Number of families**	1							
**Number of comparisons per family**	10							
**Alpha**	0,05							
**Tukey's multiple comparisons test**	**Mean Diff,**	**95% CI of diff,**	**Significant?**	**Summary**				
Men								
**1-3 vs. 4-8**	13,12	-3,359 to 29,61	No	ns				
**1-3 vs. 9-12**	2,533	-14,95 to 20,02	No	ns				
**1-3 vs. >12**	20,04	2,555 to 37,52	Yes	*				
**1-3 vs. Nonvaccinated**	31,67	15,19 to 48,16	Yes	****				
**4-8 vs. 9-12**	-10,59	-28,07 to 6,892	No	ns				
**4-8 vs. >12**	6,914	-10,57 to 24,40	No	ns				
**4-8 vs. Nonvaccinated**	18,55	2,065 to 35,03	Yes	*				
**9-12 vs. >12**	17,51	-0,9238 to 35,93	No	ns				
**9-12 vs. Nonvaccinated**	29,14	11,66 to 46,62	Yes	***				
**>12 vs. Nonvaccinated**	11,63	-5,849 to 29,12	No	ns				
Woman								
**1-3 vs. 4-8**	5,540	-13,89 to 24,97	No	ns				
**1-3 vs. 9-12**	12,95	-7,560 to 33,45	No	ns				
**1-3 vs. >12**	7,931	-11,49 to 27,36	No	ns				
**1-3 vs. Nonvaccinated**	32,87	14,16 to 51,57	Yes	****				
**4-8 vs. 9-12**	7,406	-11,17 to 25,98	No	ns				
**4-8 vs. >12**	2,391	-14,98 to 19,77	No	ns				
**4-8 vs. Nonvaccinated**	27,33	10,76 to 43,89	Yes	***				
**9-12 vs. >12**	-5,015	-23,59 to 13,56	No	ns				
**9-12 vs. Nonvaccinated**	19,92	2,101 to 37,74	Yes	*				
**>12 vs. Nonvaccinated**	24,94	8,370 to 41,50	Yes	***				
**Test details**	**Mean 1**	**Mean 2**	**Mean Diff,**	**SE of diff,**	**N1**	**N2**	**q**	**DF**
Men								
**1-3 vs. 4-8**	46,60	33,47	13,12	5,903	10	10	3,145	78
**1-3 vs. 9-11**	46,60	44,06	2,533	6,261	10	8	0,5723	78
**1-3 vs. >12**	46,60	26,56	20,04	6,261	10	8	4,527	78
**1-3 vs. Nonvaccinated**	46,60	14,92	31,67	5,903	10	10	7,589	78
**4-8 vs. 9-11**	33,47	44,06	-10,59	6,261	10	8	2,392	78
**4-8 vs. >12**	33,47	26,56	6,914	6,261	10	8	1,562	78
**4-8 vs. Nonvaccinated**	33,47	14,92	18,55	5,903	10	10	4,444	78
**9-11 vs. >12**	44,06	26,56	17,51	6,599	8	8	3,751	78

**9-11 vs. Nonvaccinated**	44,06	14,92	29,14	6,261	8	10	6,583	78
**>12 vs. Nonvaccinated**	26,56	14,92	11,63	6,261	8	10	2,628	78
Woman								
**1-3 vs. 4-8**	43,28	37,74	5,540	6,956	6	9	1,126	78
**1-3 vs. 9-11**	43,28	30,33	12,95	7,343	6	7	2,493	78
**1-3 vs. >12**	43,28	35,34	7,931	6,956	6	9	1,612	78
**1-3 vs. Nonvaccinated**	43,28	10,41	32,87	6,698	6	11	6,939	78
**4-8 vs. 9-11**	37,74	30,33	7,406	6,651	9	7	1,575	78
**4-8 vs. >12**	37,74	35,34	2,391	6,222	9	9	0,5435	78
**4-8 vs. Nonvaccinated**	37,74	10,41	27,33	5,932	9	11	6,515	78
**9-11 vs. >12**	30,33	35,34	-5,015	6,651	7	9	1,066	78
**9-11 vs. Nonvaccinated**	30,33	10,41	19,92	6,381	7	11	4,415	78
**>12 vs. Nonvaccinated**	35,34	10,41	24,94	5,932	9	11	5,945	78

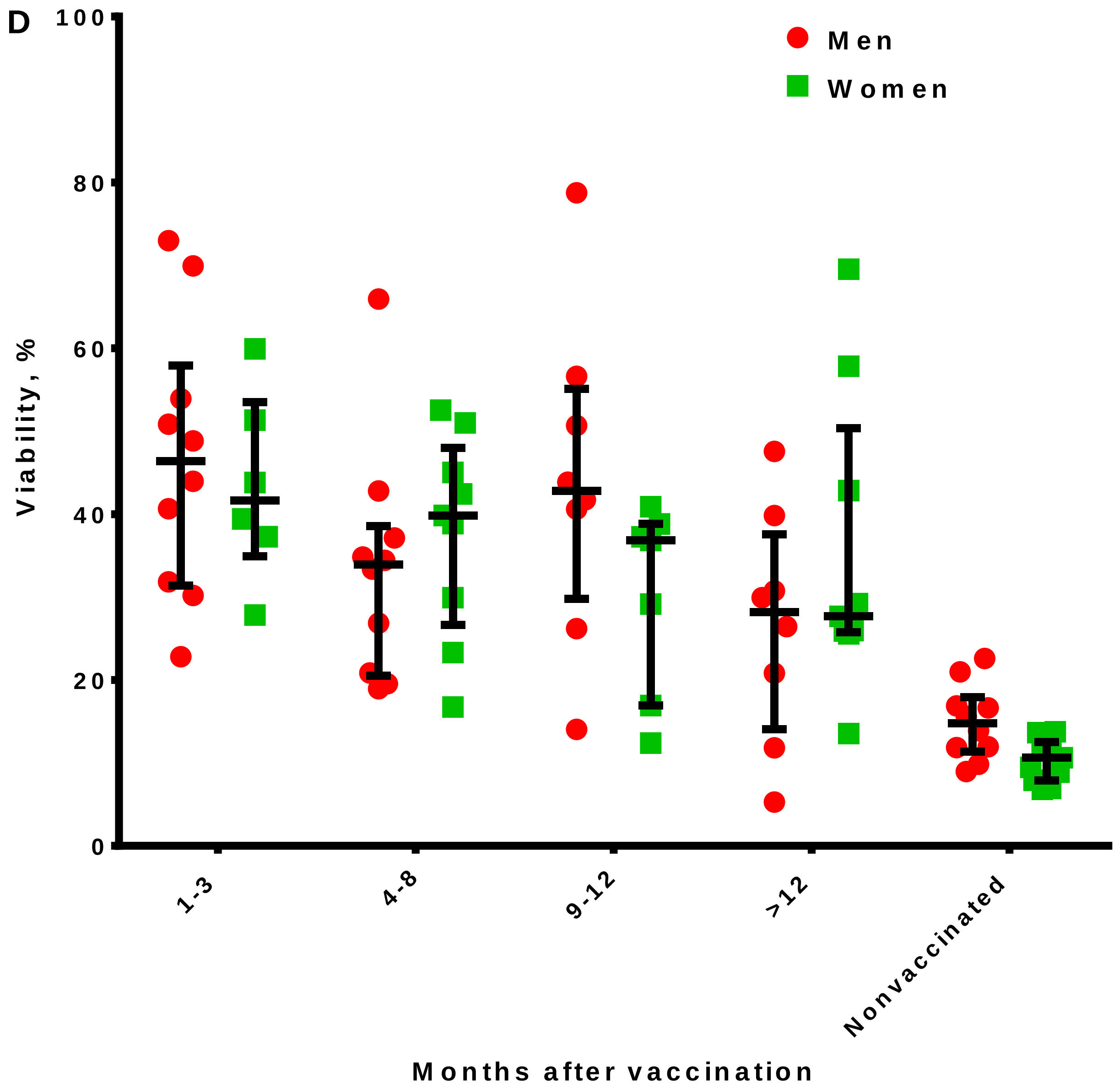

Supplement: S23 Dataset — (PDF) [file pone.0260202.s038.pdf]
