# Supplementary material for: Characterization of the adaptive immune response of donors receiving live anthrax vaccine
Source: PLoS One. 2021 Dec 20;16(12):e0260202. doi: 10.1371/journal.pone.0260202 (PMC8687594; doi:10.1371/journal.pone.0260202)
Supplement: S24 Dataset — (PDF) [file pone.0260202.s039.pdf]

Level of specific IgG PA, LF of *B. anthracis* and domains and levels of TNA in the samples of blood serum from the donors in the control group.

	IgG titers to										
Serum samples	PA fill-length	LF fill-length	Spores	PA-D1	PA-D2	PA-D3	PA-D4	LF-D1	LF-D2.3	LF-D4	TNA
1	100	50	200	100	100	25	25	0	50	0	22,5884
2	0	25	100	25	25	0	0	0	0	0	13,64473
3	0	25	0	25	0	50	0	0	0	0	7,89494
4	400	200	0	0	0	100	100	25	0	0	11,95774
5	200	50	50	500	0	50	50	0	0	25	16,8835
6	50	200	200	100	100	100	0	0	0	0	12,5392
7	200	0	50	50	50	50	50	0	0	100	9,47738
8	400	50	100	50	50	0	0	0	0	50	13,88594
9	0	100	800	50	25	25	0	50	25	0	20,9758
10	50	50	400	0	0	400	200	100	0	25	11,85338
11	400	25	0	0	0	0	50	100	0	0	6,9327
12	100	0	50	0	0	100	50	50	0	0	9,833217
13	0	0	0	25	25	25	0	50	0	0	13,74366
14	25	0	50	25	50	50	0	100	0	0	15,6362
15	400	0	100	0	0	0	25	50	0	0	6,8291
16	50	0	50	0	0	400	400	200	0	0	8,9672
17	0	0	25	25	25	100	0	0	0	0	16,6483
18	100	100	25	25	25	25	25	0	0	0	11,81109
19	50	200	0	0	0	0	0	50	0	50	12,082
20	25	100	100	0	0	25	0	0	0	0	10,6428
21	200	0	0	100	100	0	25	0	0	0	8,88675
% of positive samples	71%	62%	71%	62%	47%	71%	47%	33%	9%	19%	
